# Fungal-Bacterial Combinations in Plant Health under Stress: Physiological and Biochemical Characteristics of the Filamentous Fungus *Serendipita indica* and the Actinobacterium *Zhihengliuella* sp. ISTPL4 under In Vitro Arsenic Stress

**DOI:** 10.3390/microorganisms12020405

**Published:** 2024-02-17

**Authors:** Neha Sharma, Monika Koul, Naveen Chandra Joshi, Laurent Dufossé, Arti Mishra

**Affiliations:** 1 Amity Institute of Microbial Technology, Amity University, Noida 201313, India; nehasharma96500@gmail.com (N.S.); ncjoshi@amity.edu (N.C.J.); 2 Department of Botany, Hansraj College, University of Delhi, Delhi 110007, India; drmkoul@gmail.com; 3 Chemistry and Biotechnology of Natural Products, CHEMBIOPRO, Université de La Réunion, ESIROI Agroalimentaire, 15 Avenue René Cassin, CS 92003, CEDEX 9, F-97744 Saint-Denis, France; 4 Umeå Plant Science Center, Department of Plant Physiology, Umeå University, 90187 Umeå, Sweden

**Keywords:** *Serendipita indica*, arsenic, heavy metal stress, secondary metabolites, *Oryza sativa*

## Abstract

Fungal-bacterial combinations have a significant role in increasing and improving plant health under various stress conditions. Metabolites secreted by fungi and bacteria play an important role in this process. Our study emphasizes the significance of secondary metabolites secreted by the fungus *Serendipita indica* alone and by an actinobacterium *Zhihengliuella* sp. ISTPL4 under normal growth conditions and arsenic (As) stress condition. Here, we evaluated the arsenic tolerance ability of *S. indica* alone and in combination with *Z.* sp. ISTPL4 under in vitro conditions. The growth of *S. indica* and *Z.* sp. ISTPL4 was measured in varying concentrations of arsenic and the effect of arsenic on spore size and morphology of *S. indica* was determined using confocal microscopy and scanning electron microscopy. The metabolomics study indicated that *S. indica* alone in normal growth conditions and under As stress released pentadecanoic acid, glycerol tricaprylate, L-proline and cyclo(L-prolyl-L-valine). Similarly, d-Ribose, 2-deoxy-bis(thioheptyl)-dithioacetal were secreted by a combination of *S. indica* and *Z.* sp. ISTPL4. Confocal studies revealed that spore size of *S. indica* decreased by 18% at 1.9 mM and by 15% when in combination with *Z.* sp. ISTPL4 at a 2.4 mM concentration of As. Arsenic above this concentration resulted in spore degeneration and hyphae fragmentation. Scanning electron microscopy (SEM) results indicated an increased spore size of *S. indica* in the presence of *Z.* sp. ISTPL4 (18 ± 0.75 µm) compared to *S. indica* alone (14 ± 0.24 µm) under normal growth conditions. Our study concluded that the suggested combination of microbial consortium can be used to increase sustainable agriculture by combating biotic as well as abiotic stress. This is because the metabolites released by the microbial combination display antifungal and antibacterial properties. The metabolites, besides evading stress, also confer other survival strategies. Therefore, the choice of consortia and combination partners is important and can help in developing strategies for coping with As stress.

## 1. Introduction

Various anthropogenic activities such as mining, modern agricultural practices, and industrialization have had long-term harmful impacts on the environment [1]. These factors are responsible for increasing the concentration of heavy metals in soil and water, thus leading to several environmental issues. Arsenic (As) is a highly toxic heavy metal that poses risk to millions of people worldwide [2]. Arsenic contamination in drinking water and groundwater used for irrigation is a global problem that not only affects agricultural productivity but also the ecosystem, as As uptake occurs in plant roots and is further translocated to different plant tissues, including the edible parts, and therefore enters the ecosystem through the food chain [3]. Arsenite and arsenate are the two forms of arsenic, arsenite being more toxic. Roots are the prime site of arsenic exposure; thus root proliferation and extension are affected the most. The translocation of arsenic occurs from root to shoot, and the uptake in the aerial parts affects plant growth and reproduction by inhibiting cell division [4]. Therefore, it is essential to remove these heavy metals from the contaminated soil used for growing crop plants.

Microbes including fungal and bacterial species have the potential to cope with heavy metal stress. These microbes also aid in the growth and development of plants by increasing nutrient absorption and assimilation [5]. Microbes release various molecules including siderophores, extracellular polysaccharides, ammonia, and secondary metabolites that help plants to cope with biotic and abiotic stress [6]. Secondary metabolites are chemical substances that protect plants under stress conditions. They are not directly involved in plant growth and development but indirectly boost plant growth by acting as effective defense agents against various phytopathogens. They also help in scavenging free radicals generated during oxidative stress in plants [7].

Metabolites secreted by bacteria and fungi in the rhizosphere are not directly connected with plant growth and reproduction but they participate in rhizosphere ecological interactions [8]. Lipopeptides, phytohormones, their precursors, antimicrobial peptides, siderophores, metallothioneins, and volatile organic compounds are some examples of secondary metabolites released by the microbes [9]. Secondary metabolites, such as acyl-homoserine lactone (acyl-HSL), that act as autoinducer signaling molecules and help in cell-to-cell communication and crosstalk are also secreted by some microbes. Siderophores are some other secondary metabolites released by microbes that help in iron uptake from surroundings. Siderophores-releasing microbes help in iron uptake by the plants also [9]. Other metabolites including metallothioneins are also secreted by various microbes and plants. These are the chelating molecules that help in heavy metal detoxification [10]. These metallothioneins have tiny cysteine-rich proteins which bind with heavy metals thereby helping in metal storage and detoxification. These metallothioneins are mostly released in the presence of heavy metals such as Cd^2+^, Hg^2+^, Pb^2+^ and Ar.

In this study, we studied various secondary metabolites released by *S. indica* alone and with *Zhihengliuella* sp. ISTPL4 under normal growth conditions and under arsenic stress. *S. indica* is an endophytic fungus that boosts plant growth by colonizing the roots of various plants and confers resistance against abiotic and biotic stress conditions [11,12,13]. *Zhihengliuella* sp. ISTPL4 is an actinobacterium, isolated from Pangong Lake, Ladakh, Jammu and Kashmir, India. The interaction between *Zhihengliuella* sp. ISTPL4 and *S. indica* has been previously reported in a systemic study [14]. The role of this microbial combination has also been deciphered in plant growth promotion. In the present study, we checked the significance of secondary metabolites secreted by a combination of *S. indica* and *Z.* sp. ISTPL4 and found out which metabolites are released in response to combined microbial treatments.

## 2. Materials and Methods

### 2.1. Microbe Culture and Conditions

The fungal disc of *S. indica* was inoculated into Hill and Kaefer agar plates and incubated for 14 days at 28–30 °C (with an agitation rate of 120 rpm for broth). Similarly, *S. indica* growth was also assessed in combination with *Z.* sp. ISTPL4. Bacterial culture was streaked around the periphery after five days of fungal inoculation (5 dafi) [15].

### 2.2. Effect of Arsenic on Fungal Growth

The arsenic tolerance capability of *S. indica* was checked individually as well as in combination under in vitro conditions. The concentration of arsenic used was up to 2.4 mM [16]. Dry cell weight of *S. indica* alone and in combination was estimated (control and treated cultures) to check the effect of arsenic on the growth of *S. indica* [14].

### 2.3. Spore Morphology

To study spore morphology SEM studies were carried out for *S. indica* alone and in combination with *Z.* sp. ISTPL4. A section of fungal discs was fixed in 2.5% glutaraldehyde (control and interaction plates) followed by incubation at room temperature (RT) for 1 h. After this, samples were centrifuged at 5000 rpm for 5 min followed by washing with 0.1 M phosphate buffer (pH 7) followed by centrifugation. The resulting pellet was dissolved in 0.1% filter sterilized silver nitrate (AgNO_3_) solution and then incubated for an hour at RT. Samples were gradually dehydrated in a series of ethanol from 30–90% for 15 min. The final step was repeated three times in 100% ethanol for 5 min each. Resulting samples were dehydrated, air dried and placed in aluminum double adhesive carbon conductive tape gold-coated specimens in a Quorum Q150ES coater and then observed under a scanning electron microscope (model Zeiss EVO 18; Raipur, India) [14].

Confocal microscopy was also carried out to check spore size and spore morphology of *S. indica* alone and in combination with *Z.* sp. ISTPL4 under normal growth conditions and under As stress. Spore isolation was done by adding 1 mL of a 0.02% autoclaved Tween 20 on freshly grown cultures of *S. indica* alone and with *Z.* sp. ISTPL4 (control and As-exposed plates) followed by gentle scraping and centrifugation at 5000 rpm for 10–15 min. The pellet was mixed with autoclaved distilled water, a spore count was conducted, and their concentration was maintained at 4.8 × 10^5^ spores/mL. The resulting sample was analyzed under a Nikon confocal microscope (Model: Nikon A1) at 60× magnification by using NIS Elements software version 4.6 (Nikon, Tokyo, Japan) (Amity Institute of Microbial Technology, Amity University, Noida, India) [17].

### 2.4. Biotransformation of Arsenic

The arsenic oxido-reduction potential of *S. indica* and *Z.* sp. ISTPL4 was determined. For this, nutrient agar was supplemented with 500 ppm of As (V) and the fungal disc was inoculated followed by an incubation of 15 days at 28 ± 2 °C. Spot inoculation of *Z.* sp. ISTPL4 was conducted, and the bacterial culture was kept for incubation at 37 °C for 72 h. After fungal and bacterial growth, 100 µL AgNO_3_ (0.1 M) solution was flooded over the grown fungal and bacterial plates and a change in color was observed [18].

### 2.5. Gas Chromatography and Mass Spectroscopy Analysis (GC-MS)

GC-MS analysis was performed for the secondary metabolic profiling in *S. indica* alone and in combination with *Z.* sp. ISTPL4 under normal growth conditions and in the presence of arsenic stress. The fungal disc was inoculated in H and K broth and then kept for incubation for 2 weeks. A similar protocol was followed for the combined growth of *S. indica* and *Z.* sp. ISTPL4. The bacterial culture was inoculated in H and K broth after 5 days of fungal inoculation (5 dafi) followed by incubation (2 weeks). A 100 mL sample was taken and then centrifuged at 9000 rpm for 10 min. The supernatant was collected and mixed with an equal volume of ethyl acetate. The resulting solution was shaken for 3 h. The sample was then concentrated on a rotatory evaporator. The final volume was mixed with methanol. This concentrated sample was used for GC-MS analysis for secondary metabolite detection.

GC-MS analysis was carried out in a Perkin-Elmer GC Clarus 500 system, which included an Elite-5MS (5% diphenyl/95% dimethyl poly siloxane) fused capillary column (30 × 0.25 μm ID × 0.25 μm df) and an AOC-20i auto-sampler and Gas Chromatograph interfaced to a Mass Spectrometer (GC-MS). An electron ionization device with an ionization energy of 70 eV was used in electron impact mode for GC-MS detection. A split ratio of 10:1 was used with an injection volume of 2 μL and helium gas (99.999%) was used as the carrier gas at a steady flow rate of 1 mL/min. The temperature of the ion source was 200 °C, the injector was kept at 250 °C, and the oven was set at 110 °C (isothermal for two minutes) which was further increased by 10 °C/min to 200 °C, and 5 °C/min to 280 °C, and stopped with a 9-min isothermal at 280 °C. Mass spectra with fragments from 45 to 450 Da and a scan interval of 0.5 s were obtained at 70 eV. The GC-MS was run for 36 min, with a solvent delay of 0 to 2 min. By comparing the average peak area of each component to the total areas, the relative percentage amount was determined. The Turbo-Mass ver-5.2 software was utilized to handle mass spectra and chromatograms. Metabolite identification, retention time (R.T.) and mass to charge ratio (*m*/*z*) was analyzed as listed in the NIST library [19,20,21].

### 2.6. Clustered Heatmap of Metabolites

A heatmap of common metabolites secreted by the individual culture of *S. indica* as well as in combination with *Z.* sp. ISTPL4 under normal growth conditions and in the presence of As stress was prepared by versatile matrix visualization and analysis software (https://software.broadinstitute.org/morpheus/ (accessed on 1 June 2022)).

### 2.7. Statistical Method

The experiments were repeated five times. Statistical analysis was conducted utilizing two-way ANOVA and student’s *t* test (*p* < 0.05) using online OPSTAT software (version 6.8). All the variables were subjected to statistical analysis. 

## 3. Results

### 3.1. Microbial Conditions

**Figure 1:**
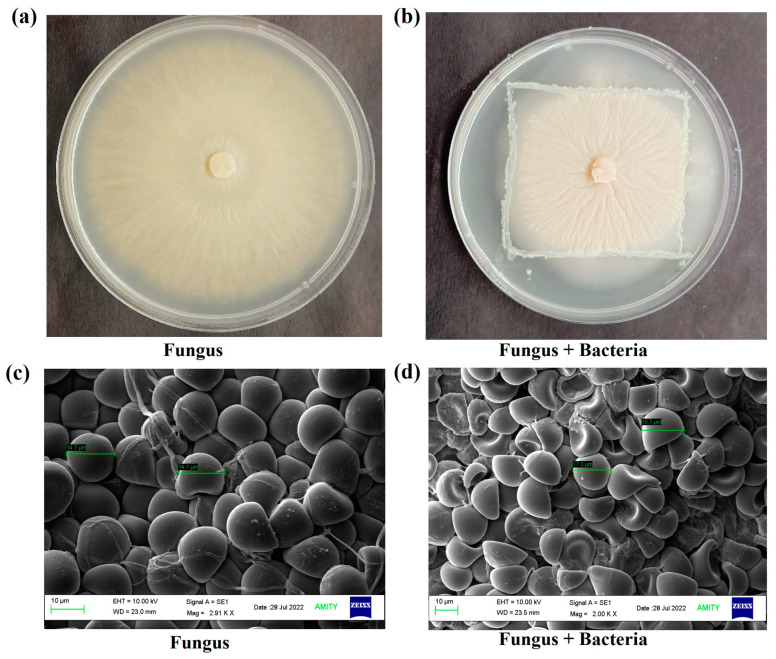
Growth of (**a**) *Serendipita indica* (Fungus) alone (**b**) in the presence of *Zhihengliuella* sp. ISTPL4 (Bacteria) on a Hill and Kaefer agar plate (**c**) morphology of *S. indica* spores visualized using scanning electron microscopy (SEM) at Magnification: 2910×; voltage: 10 Kv; scale: 10 µm. (**d**) Spore morphology of *S. indica* in presence of *Z.* sp. ISTPL4 at Magnification: 2000×; voltage: 10 Kv; scale: 10 µm.

**Figure 2:**
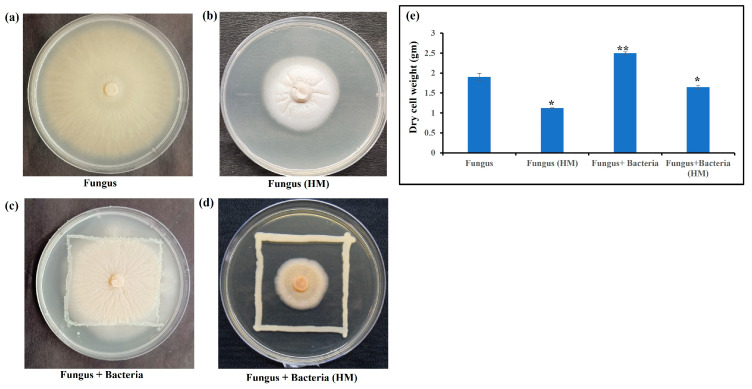
Growth of *S. indica* alone (**a**) under normal growth condition (2.9 ± 0.01 cm) (**b**) in As stress (1.6 ± 0.01 cm; 1.9 mM) (**c**) in presence of *Z.* sp. ISTPL4 under normal growth condition (2.5 ± 0.05 cm), (**d**) in presence of As stress (1.4 ± 0.03 cm; 2.4 mM) and (**e**) shows the difference in dry weight of *S. indica* alone, in the presence of arsenic stress, in combination with *Z.* sp. ISTPL4 and under arsenic stress after microbial inoculation. According to the student’s *t*-test, asterisks showed significant differences: ‘*’: *p* ≤ 0.05; ‘**’: *p* ≤ 0.01).

The growth of *S. indica* was checked alone and with *Z.* sp. ISTPL4 by measuring the hyphal radius and spore size was measured using scanning electron microscopy (Figure 1a–d). *S. indica* growth was assessed under varying concentrations of As and it was observed that *S. indica* was able to survive at 1.9 mM concentration (Figure 2). Growth of *S. indica* under normal conditions was (3.1 ± 0.025 cm) and it decreased up to (1.6 ± 0.01 cm) at 1.9 mM As concentration. *S. indica* growth was assessed in combination with *Z.* sp. ISTPL4. *S. indica* when grown in combination with *Z.* sp. ISTPL4 was able to grow at 2.4 mM concentration. The hyphal radius of *S. indica* was (2.9 ± 0.01 cm) when grown with *Z.* sp. ISTPL4 under normal growth conditions and it reduced (1.4 ± 0.03 cm) in the presence of As (Figure 2a–d).

The dry cell weight of *S. indica* alone as well as in combination with *Z.* sp. ISTPL4 was measured in the control as well as in the presence of As. The dry cell weight of *S. indica* was 69% higher than dry cell weight of *S. indica* under As stress. Similarly, dry cell weight of combination of *S. indica* and *Z.* sp. ISTPL4 was 51% more than the dry cell weight of the combination of *S. indica* and *Z.* sp. ISTPL4 under arsenic stress (Figure 2e).

### 3.2. Spore Morphology

**Figure 3:**
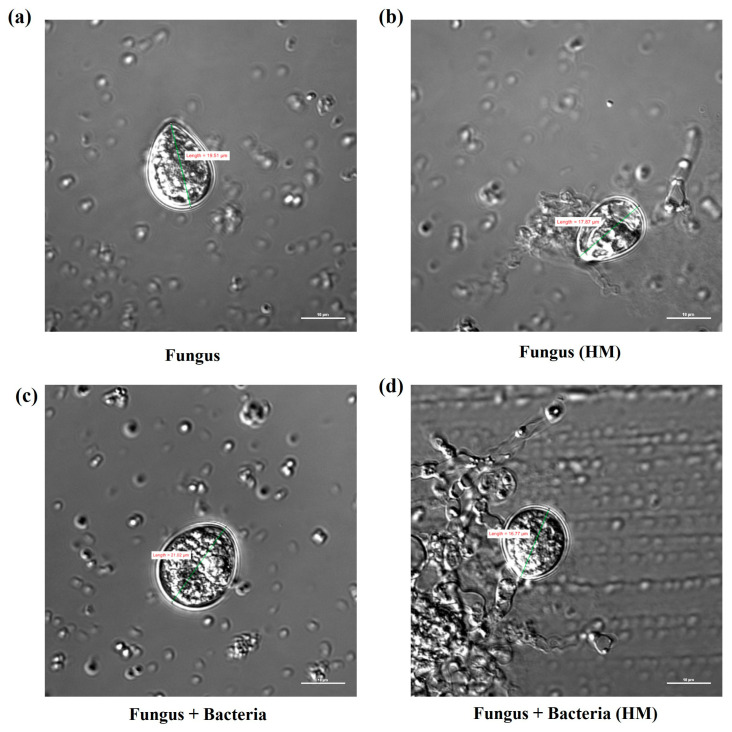
Confocal microscopy analysis shows the spore morphology and spore size of (**a**) *S. indica* alone (**b**) under arsenic stress (1.9 mM) (**c**) in combination with *Z.* sp. ISTPL4 and, (**d**) in combination with *Z.* sp. ISTPL4 under arsenic stress (2.4 mM).

Increased spore size of *S. indica* was observed in the presence of *Z*. sp. ISTPL4 (18 ± 0.75 µm) as compared to its individual culture (14 ± 0.24 µm) under normal growth conditions and confocal microscopy under normal growth conditions in scanning electron microscopy (Figure 1c–d). The spore size of *S. indica* decreased by 18% at 1.9 mM concentration and by 15% when used in combination with *Z.* sp. ISTPL4 at 2.4 mM concentration of As as observed under a confocal microscope. Increase in concentration beyond this resulted in spore degeneration and hyphae fragmentation (Figure 3a–d). The spore count was also decreased at higher concentration of arsenic. It was 4.59 × 10^5^ spores/mL in *S. indica* under normal growth conditions and it reduced to 3.68 × 10^3^ spores/mL at 1.9 mM concentration of arsenic. Similarly, the spore count of *S. indica* was slightly higher 4.65 × 10^5^ spores/mL, when it was grown in presence of *Z.* sp. ISTPL4 and at 2.4 mM concentration, it decreased to 4 × 10^3^ spores/mL.

### 3.3. Biotransformation of Arsenic

**Figure 4:**
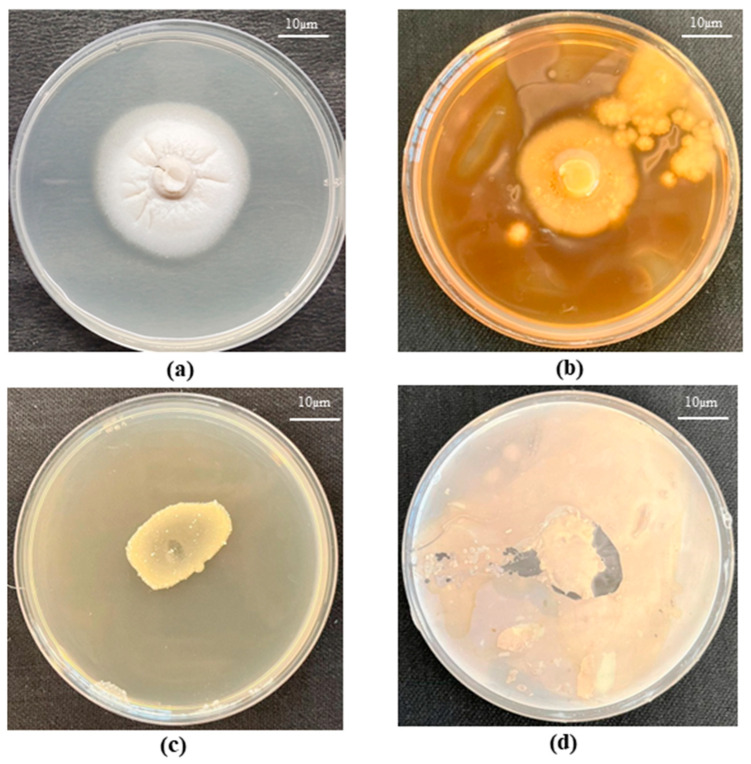
Screening of arsenate transformation ability of *S. indica* and *Z.* sp. ISTPL4: Change in color from white to yellow indicates the reduction of arsenate to arsenite (**a**) *S. indica* (control)*:* Arsenate plate without silver nitrate (**b**) *S. indica* (Test): Arsenate plate with silver nitrate (**c**) *Z.* sp. ISTPL4 (control): Arsenate plate without silver nitrate (**d**) *Z.* sp. ISTPL4 (test): Arsenate plate with silver nitrate.

The biotransformation potential of *S. indica* and *Z.* sp. ISTPL4 was checked on the basis of the color change of fungal and bacterial cultures. Color change to brown in fungal cultures indicated transformation ability of As (V) (arsenate) to As (III) (arsenite). The ability of the fungal strain to bring conversion was discovered after 72 h of incubating fungal and bacterial plates. Contact of silver nitrate with As (V) (already present in media) produced a brownish precipitate, while the association with As (III) produced a pale yellow precipitate. The results of the arsenic transformation ability of *S. indica* indicated brown color formation which denotes that this fungus has certain genes which are involved in arsenic transformation. *Z.* sp. ISTPL4 revealed no change in color which indicated no conversion of arsenic to other forms (Figure 4a–d).

### 3.4. Production of Secondary Metabolites

**Figure 5:**
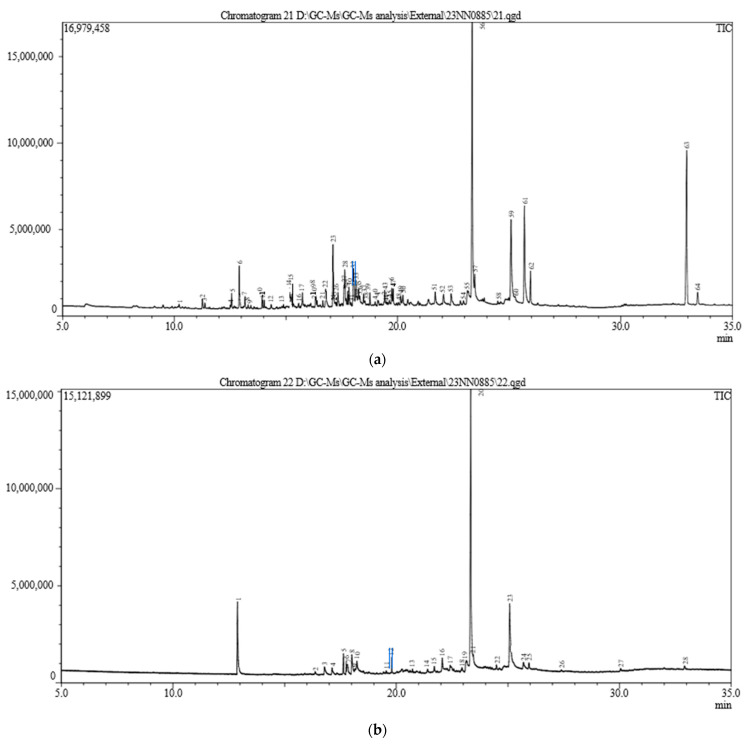
GC-MS chromatogram of secondary metabolites secreted by (**a**) *S. indica* under normal growth conditions and, (**b**) in the presence of arsenic.

**Figure 6:**
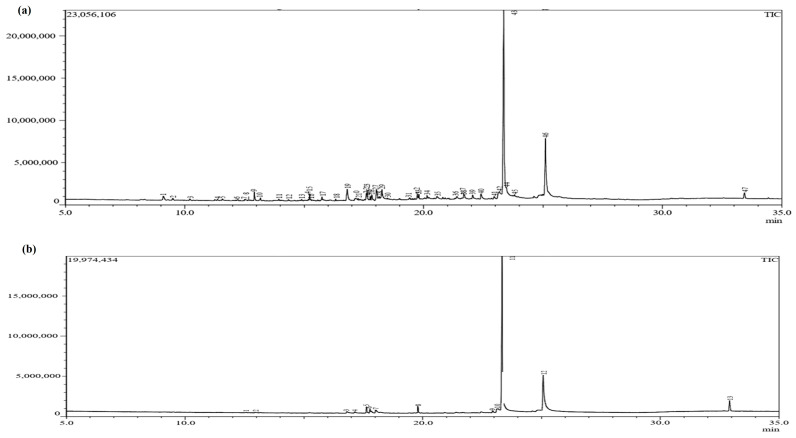
GC-MS chromatogram of secondary metabolites secreted by (**a**) a combination of *S. indica and Z*. sp. ISTPL4 under normal growth conditions and, (**b**) in the presence of arsenic.

**Figure 7:**
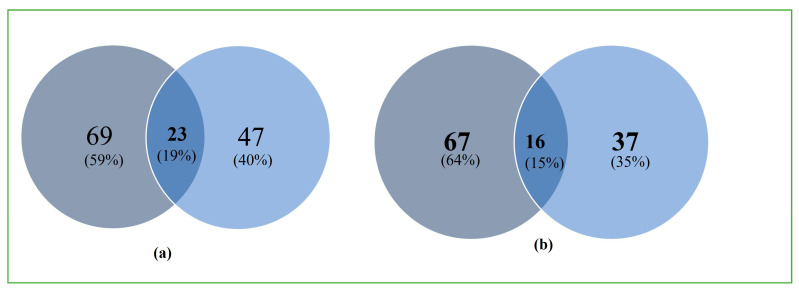
(**a**) Venn diagram representing different and common metabolites released by *S. indica* under normal growth conditions and in the presence of arsenic with common metabolites mentioned in box. (**b**) Venn diagram representing different and common metabolites released by combination of *S. indica* and *Z.* sp. ISTPL4 under normal growth conditions and in the presence of arsenic with common metabolites mentioned in box.

**Figure 8:**
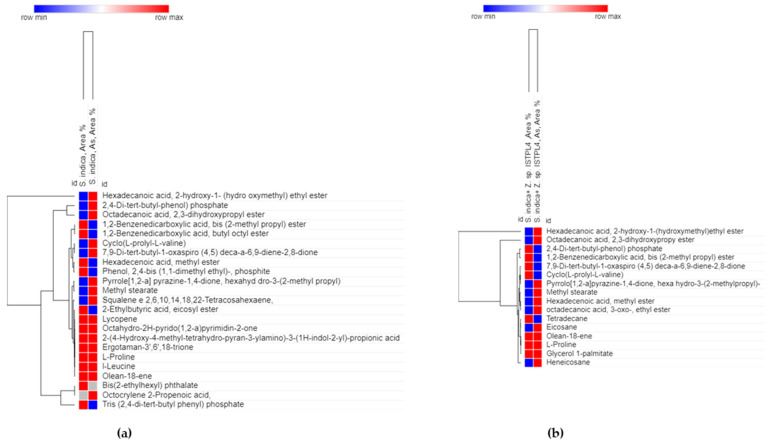
Gas chromatography-mass spectrometry (GC-MS) spectrum comparison (**a**) clustered heatmap of common metabolites secreted by *S. indica* alone under normal growth conditions and in presence of As stress (**b**) heatmap showing common metabolites secreted by combination of *S. indica* and *Z.* sp. ISTPL4 under normal growth conditions and in presence of As stress; heatmap ranging from red to blue shows high to low abundance of metabolites detected.

Secondary metabolite production in *S. indica* alone as well as in combination with *Z.* sp. ISTPL4 was checked under normal growth conditions and under As stress (Figure 5a,b). GC-MS studies revealed the presence of 69 metabolites secreted by *S. indica* under normal conditions and 47 metabolites under As stress. Among these metabolites, 23 metabolites are common including 2,4-Di-tert-butylphenol having a retention time of 12.910 and molecular weight 206, 1,2-Benzenedicarboxylic acid with a retention time of 17.125 and molecular weight of 278, respectively (Table 1). 2-Ethylbutyric acid, eicosyl ester, 2-hydroxy-1(hydroxtmethyl)ethyl ester, Octocrylene 2-propenoic acid and Squalene e 2,6,10,14,18,22- Tetracosahaxaene are some of the metabolites secreted by culture of *S. indica* alone and under arsenic stress. A total of 67 metabolites were secreted by the combination of *S. indica* and *Z.* sp. ISTPL4 under normal conditions, and 37 metabolites were secreted under arsenic stress (Figure 6a,b). Similarly, 16 metabolites were common including Cyclo(L-prolyl-L-valine), hexahydro-3-(2-methylpropyl), Eicosane, 2,4-Di-tert-butylphenol, L-Proline and Heneicosane (Table 1). All these metabolites are involved in antibacterial, antifungal, and antioxidant activities while some of them have important roles in plant growth promotion under various environmental conditions. These metabolites are also represented in the form of venn diagram (Figure 7a,b). Figure 8 shows the clustered heatmap of the secondary metabolites. These metabolites include volatile organic compounds (VOCs), hydrocarbons, amino acids, fatty acids and vitamins. Hierarchical clustering analysis (HCA) shows the comparison of common metabolites based on the area percentage detected. A heatmap ranging from red (high) to blue (low) denoted the concentration of each metabolite detected. The numerical data of metabolites were normalized to allow for clustering and color scaling based on the concentration of compounds. In Figure 8a, compounds that appeared as red were considered highly abundant, and Figure 8b showed that an equal ratio of red and blue colored compounds appeared.

## 4. Discussion

Microorganisms release various secondary metabolites to cope with abiotic stress resulting in better plant growth performance, increased photosynthesis, and the production of antioxidants [21]. Besides bacteria, fungal species also confer abiotic stress tolerance in plants by helping them to adapt to a variety of conditions, such as heat, salinity, cold, drought, and toxic metals [22]. *Fusarium culmorum*, *Azotobacter*, *Pseudomonas*, *Curvularia protuberata*, *Piriformospora indica*, *Neotyphodium lolii*, and *Trichoderma* have all been documented to confer abiotic stress tolerance [23]. Among these, *S. indica* is a plant-growth-promoting root endophytic fungus. It plays an important role in defending plants from adverse biotic and abiotic factors [24,25]. The role of various secondary metabolites secreted by *S. indica* alone and in combination with *Z.* sp. ISTPL4 was studied in the present study. *S. indica* growth was also checked in the presence of arsenic individually and in combination with *Z.* sp. ISTPL4. It was reported that *S. indica* was able to grow up to 1.9 mM and up to 2.4 mM in combination with *Z.* sp. ISTPL4 in the culture conditions. Mohd et al. (2017) studied *P. indica* growth under arsenite (III) and arsenate (V) stress and observed a reduction in the growth of fungus at higher As concentrations [16]. The spore morphology and size of *S. indica* alone and in combination was also assessed under control conditions and in As stress. It was observed that the hyphae and spores were intact at 1.9 mM As concentration and hyphae started fragmenting and spores degenerated beyond 1.9 mM concentration of As in *S. indica* alone. Similarly, spore size and morphology of *S. indica* in combination with *Z.* sp. ISTPL4 was observed in up to 2.4 mM concentration of arsenic. The morphology of *S. indica* spores was also checked in the presence of Cd by Dabral et al. 2019. It was observed that spore germination inhibited beyond a 0.1 mM concentration of Cd [26].

An Increase of 69% in dry cell weight was observed in *S. indica* when compared with dry cell weight of *S. indica* under arsenic stress. Similarly, the dry cell weight of the combined culture of *S. indica* and *Z.* sp. ISTPL4 was 51% more than a combined culture of *S. indica* and *Z.* sp. ISTPL4 under arsenic stress. The spore morphology of *S. indica* was also checked under normal conditions and in arsenic stress by confocal microscopy. It was observed that the spore degenerated beyond a 1.9 mM concentration of arsenic and beyond 2.4 mM in the microbial combination of *S. indica* and *Z.* sp. ISTPL4. The results of the spore count of *S. indica* under arsenic stress was measured which indicated a reduction in spore count to 3.68 × 10^3^ spores/mL at 1.9 mM concentration compared to the control (4.59 × 10^5^ spores/mL). The spore count of *S. indica* in the presence of *Z.* sp. ISTPL4 was also measured, which indicated an increased spore count (4.65 × 10^5^ spores/mL) under normal growth conditions, and it was reduced to 4 × 10^3^ spores/mL. The spore germination and spore size of *S. indica* alone as well as in the presence of *Z.* sp. ISTPL4 was also determined under normal growth conditions which indicated an increased spore size and germination of *S. indica* in the presence of *Z.* sp. ISTPL4 than its respective control (*S. indica*) [14].

The arsenic biotransformation ability of *S. indica* and *Z.* sp. ISTPL4 was confirmed. The results of arsenic biotransformation in *S. indica* indicated the formation of a brown-colored precipitate (Figure 4), which indicated the conversion of arsenate (v) to arsenite (III). Mohd et al. (2017) also reported the bioaccumulation and biotransformation ability of *S. indica* under arsenic stress [16]. In the case of *Z.* sp. ISTPL4, there was no change in color observed but the findings of whole genome sequencing indicated the presence of arsenic-resistant genes in *Z.* sp. ISTPL4 [18,27].

In this study, we reported the significance of various metabolites secreted by *S. indica* alone and in combination with *Z.* sp. ISTPL4 under normal and in arsenic stress. These metabolites regulate plant growth and development under stress conditions by acting as antioxidants which scavenge free radicals generated in plants during oxidative stress [28,29]. A total of 69 metabolites were produced in *S. indica* under normal conditions and 47 metabolites were produced in As stress (Supplementary File S3). Among all the metabolites analyzed, 23 metabolites were common (Table 1). These metabolites include 1,2-Benzenedicarboxylic acid, 2-ethylbutyric acid, eicosyl ester, Octocrylene 2-propenoic acid, squalene 2,6,10,14,18,22-Tetracosahaxaene, phenol and, 2,4-bis(1,1-dimwthylethyl)-phosphite. The chromatogram of each metabolite has been shown in Supplementary Files S1 and S4. Similarly, 67 metabolites were secreted by a combination of *S. indica* and *Z.* sp. ISTPL4 under normal conditions and 37 metabolites were secreted by a combination of *S. indica* and *Z.* sp. ISTPL4 under arsenic stress (Supplementary File S4). Among them, 16 metabolites were common including Cyclo(L-prolyl-L-valine), hexa hydro-3-(2-methylpropyl), Eicosane, 2,4-Di-tert-butylphenol, methyl ester, Methyl stearate and L-Proline (Table 1). These metabolites have different functions such as antifungal activities, antimicrobial activities, and antioxidant activities [28,29,30]. Some of these metabolite such as glycine, Quinoline-4-carboxamide 2-phenyl-N-n.-octyl are antimicrobial and reduces stress in plants [31,32]. Other metabolites including Dodecane, 4,6-dimethyl, Tetradecane, I-Hexadecanol, Nonadecane and, Heneicosane were also identified in the individual culture of *S. indica*. Similarly, 5-Azacytosine, *N*,*N*,*O*-trimethyl, 5-Nitroso-2,4,6-triaminopyrimidine, d-Ribose, 2-deoxy-bis(thioheptyl)-dithioacetal, 5-Nitroso-2,4,6-triaminopyrimidine were secreted by combination of *S. indica* and *Z.* sp. ISTPL4. These metabolites have antimicrobial, antioxidant and nematicidal activities. I-hexadecanol is a major volatile compound produced by molds and acts as an antifeedant in different species of aphids [23,25,32,33]. Heneicosane has antimicrobial activities against *Streptococcus pneumonia* and *Aspergillus fumigatus* and Cyclo(L-propyl-L-valine) is a class of diketopiperazines (DKPs). These metabolites are mainly involved in interactions between mixed microbial communities [33,34]. The secretion of this metabolite by microbes can be a possible reason for the positive interaction of *S. indica* and *Z.* sp. ISTPL4. Other metabolites including l-(+)-Ascorbic acid 2,6-dihexadecanoate, Docosanoic acid, ethyl ester, Isopropyl palmitate, 2-Methyltetracosane, n-Hexane, 13-Docosenamide (Z) have been reported. These metabolites play a crucial role in antiallergic, antibacterial, antioxidant, termiticide and antiviral activity, antimicrobial and free radical scavenging activity [35,36]. Tetratriacontyl heptafluorobutyrate, myristic acid, glycidyl ester, tetradecanoic acid, 2-Propenoic acid, 3-(4-methoxyphenyl) are the metabolites released by *S. indica* in the presence of As. These metabolites have antifungal and antimicrobial activity [6,34,37]. Metabolites including 2,3-dihydroxypropyl ester Stearin, Eicosane, 3-Indol-1-yl-propionic acid, methyl ester and, Glycerol tricaprylate, have antimicrobial, antifungal, probiotics and antioxidant activities [38,39,40]. L-Proline, glycine, squalene, heptadecane, 7,9 di-tert butyl-1-oxaspiro(4,5) deca-a-6,9-diene-2,8-dione, Dodecane,4,6-dimethyl, Tridecanoic acid, 12-methyl-, methyl ester, 2-methyl tetracosane and quercetin play an essential role in plant growth and development under abiotic stress [41,42,43,44,45]. These metabolites protect plants under stress by acting as antioxidants, promoting signaling cascade in plants for enhancing plant growth and scavenging free radicals generated during oxidative stress in plants. Quercetin is a polyphenolic compound with antioxidant properties secreted by microbial combinations.

**Figure 9:**
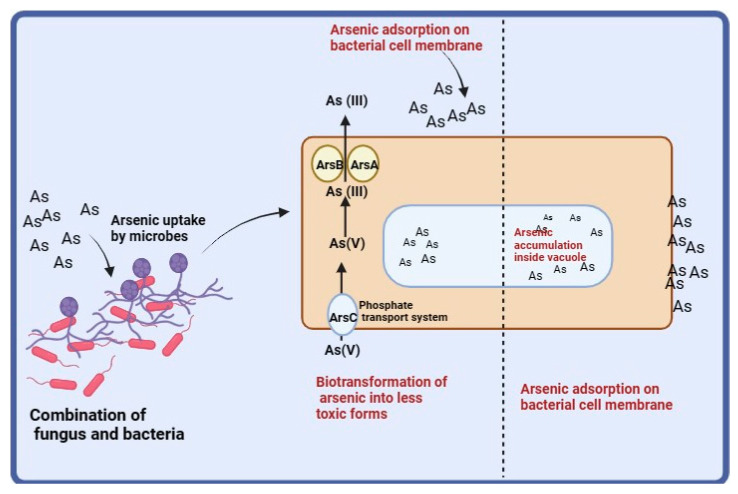
Schematic representation of arsenic uptake and efflux by *S. indica* and *Z.* sp. ISTPL4.

The study suggests that an active efflux system operates in the removal of arsenic by *S. indica* and *Z.* sp. ISTPL4. *S. indica* and *Z.* sp. ISTPL4 uptakes arsenate through phosphate transporters (Ars C) and converts it into arsenite by arsenate reductase. The converted form of arsenite (III) is then shunted out. The presence of the ArsC gene is also reported in *Z.* sp. ISTPL4 which also confers arsenic tolerance ability of this actinobacterium. This combination of microbes can be used to mitigate the toxic effects of arsenic in the contaminated environments and increase sustainable agriculture in the soils that are contaminated (Figure 9).

## 5. Conclusions

Our study concluded the significance of various metabolites secreted by *S. indica* alone and in combination with *Z.* sp. ISTPL4 under normal environmental conditions and in the presence of arsenic stress. The secretion of Cyclo(L-propyl-L-valine) by both *S. indica* and *Z.* sp. ISTPL4 indicated the possible reason for their interaction. Also, the arsenic tolerance ability of *S. indica* was checked individually and in combination with *Z.* sp. ISTPL4 in this study. Results of the biotransformation experiment also indicated the arsenic tolerance ability of *S. indica* and *Z.* sp. ISTPL4. However, these microbes can be used to mitigate arsenic stress. The presence of various metabolites also indicated the capability of *S. indica* and *Z.* sp. ISTPL4 in plant growth and stress alleviation under various biotic and abiotic factors. Our data are largely confirmed by other scientific literature, which has shown that these microbial strains can efficiently remove arsenic from contaminated environments.

## Figures and Tables

**Table 1:** List of common metabolites secreted by *S. indica* alone and in combination with *Z.* sp. ISTPL4 under normal growth conditions and in presence of As stress.

S. No.	Metabolites (*S. indica*/*S. indica*; As)	Metabolites (*S. indica* + *Z.* sp. ISTPL4/*S. indica* + *Z.* sp. ISTPL4; As)
1	2,4-Di-tert-butyl-phenol) phosphate	2,4-Di-tert-butyl-phenol) phosphate
2	Cyclo(L-prolyl-L-valine)	Cyclo(L-prolyl-L-valine)
3	1,2-Benzenedicarboxylic acid, bis (2-methyl propyl) ester	1,2-Benzenedicarboxylic acid, bis (2-methyl propyl) ester
4	7,9-Di-tert-butyl-1-oxaspiro (4,5) deca-a-6,9-diene-2,8-dione	7,9-Di-tert-butyl-1-oxaspiro (4,5) deca-a-6,9-diene-2,8-dione
5	Hexadecenoic acid, methyl ester	Hexadecenoic acid, methyl ester
6	Pyrrole[1,2-a]pyrazine-1,4-dione, hexahyd dro-3-(2-methyl propyl)	octadecanoic acid, 3-oxo-, ethyl ester
7	1,2-Benzenedicarboxylic acid, butyl octyl ester	Olean-18-ene
8	Methyl stearate	L-Proline
9	2-Ethylbutyric acid, eicosyl ester	Glycerol 1-palmitate
10	2-(4-Hydroxy-4-methyl-tetrahydro-pyran-3-ylamino)-3-(1H-indol-2-yl)-propionic acid	Hexadecanoic acid, 2-hydroxy-1-(hydroxymethyl)ethyl ester
11	Lycopene	Octadecanoic acid, 2,3-dihydroxypropy ester
12	Hexadecanoic acid, 2-hydroxy-1-(hydro oxymethyl) ethyl ester	Methyl stearate
13	Bis(2-ethylhexyl) phthalate	Pyrrolo[1,2-a]pyrazine-1,4-dione, hexa hydro-3-(2-methylpropyl)-
14	Octocrylene 2-Propenoic acid,	Tetradecane
15	Octadecanoic acid, 2,3-dihydroxypropyl ester	Eicosane
16	Squalene e 2,6,10,14,18,22-Tetracosahexaene,	Heneicosane
17	Phenol, 2,4-bis (1,1-dimethyl ethyl)-, phosphite	
18	Tris (2,4-di-tert-butyl phenyl) phosphate	
19	Octahydro-2H-pyrido(1,2-a)pyrimidin-2-one	
20	Olean-18-ene	
21	l-Leucine	
22	L-Proline	
23	Ergotaman-3′,6′,18-trione	

## Data Availability

Data are contained within the article.

## Supplementary Materials

The following supporting information can be downloaded at: https://www.mdpi.com/article/10.3390/microorganisms12020405/s1, Supplementary File S1: Mass fragmentation pattern and structural elucidations of common metabolites secreted by *Serendipita indica* under normal growth conditions and in presence of arsenic stress; Supplementary File S2: Mass fragmentation patterns and structural elucidations of common secondary metabolites secreted by *Serendipita indica* and *Zhihengliuella* sp. ISTPL4 under normal growth conditions and in the presence of arsenic stress; Supplementary File S3: Secondary metabolites production by *Serendipita indica* and *Zhihengliuella* sp. ISTPL4 under normal growth conditions and in presence of As stress; Supplementary file S4: Secondary metabolites production by *Serendipita indica* and *Zhihengliuella* sp. ISTPL4 under normal growth conditions and in presence of As stress. Refs. [40,41,42,43,44,45,46,47,48,49,50,51,52,53,54,55,56,57,58,59,60,61,62,63,64,65,66,67,68,69,70,71,72,73,74,75,76,77,78,79,80,81,82,83,84,85,86,87,88,89,90,91,92,93,94,95,96,97,98,99,100,101,102,103,104,105,106,107,108,109,110,111,112,113,114,115,116,117,118,119,120,121,122,123,124,125,126,127,128,129,130,131,132,133,134,135,136,137,138,139,140,141,142,143,144,145,146,147,148,149,150,151,152,153,154] can be found in Supplementary Materials.
