# Supplementary material for: Fungal-Bacterial Combinations in Plant Health under Stress: Physiological and Biochemical Characteristics of the Filamentous Fungus Serendipita indica and the Actinobacterium Zhihengliuella sp. ISTPL4 under In Vitro Arsenic Stress
Source: Microorganisms. 2024 Feb 17;12(2):405. doi: 10.3390/microorganisms12020405 (PMC10892705; doi:10.3390/microorganisms12020405)
Supplement: Supplementary file 1 [file microorganisms-12-00405-s001.zip › microorganisms-2846470-supplementary.pdf]

Supplementary Material

A total of 67 metabolites were produced by a combination of *S. indica* and *Z.* sp. ISTP4 under normal conditions and 37 metabolites were produced in As stress (out of which 16 metabolites were common)

1. Compound name: 2,4-Di-tert-butyl-phenol) phosphate

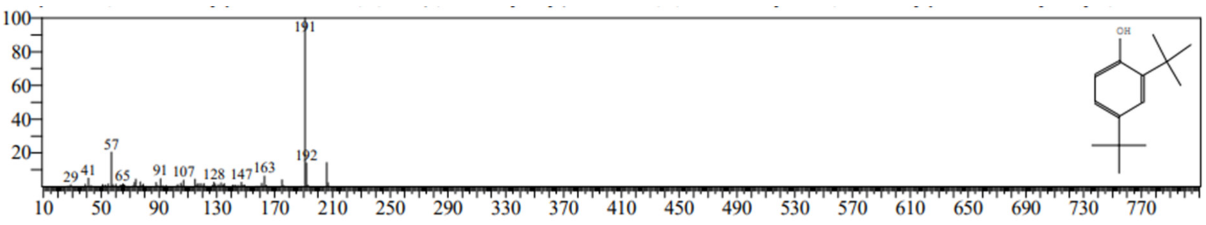

2. Compound name: Cyclo(L-prolyl-L-valine)

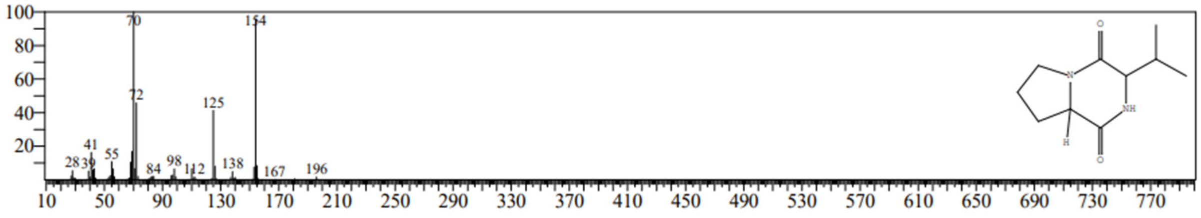

3. Compound name: 1,2-Benzenedicarboxylic acid, bis (2-methyl propyl) ester

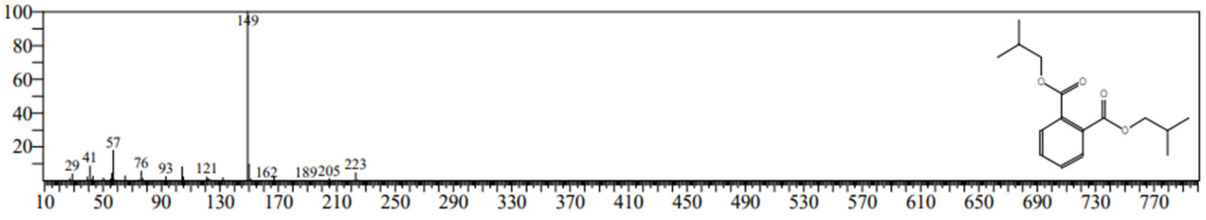

4. Compound name: Hexadecenoic acid, methyl ester

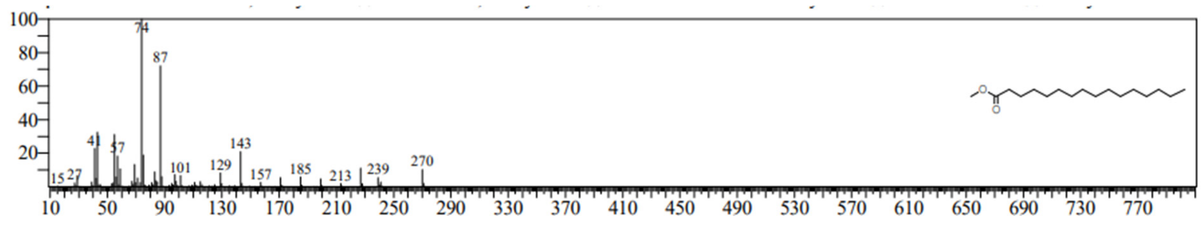

5. Compound name: Pyrrole[1,2-a] pyrazine-1,4-dione, hexahyd dro-3-(2-methyl propyl)

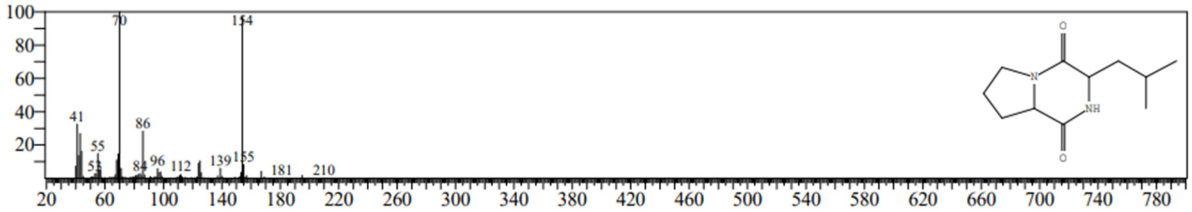

6. Compound name: Methyl stearate

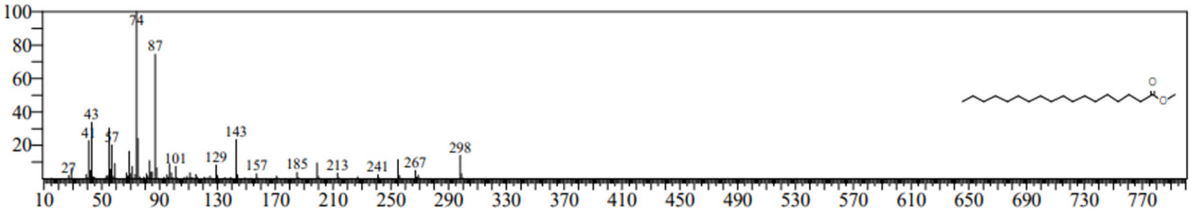

7. Compound name: 2-Ethylbutyric acid, eicosyl ester

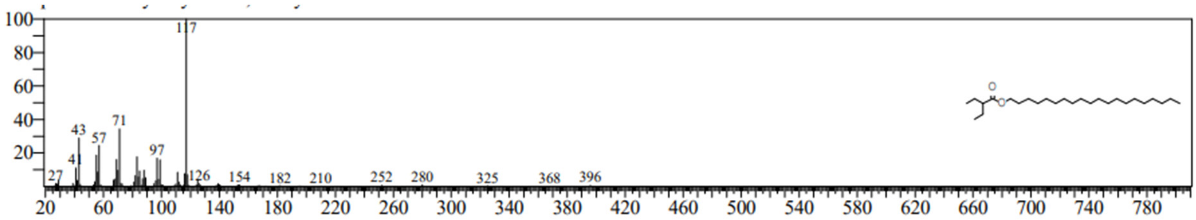

8. Compound name: 2-(4-Hydroxy-4-methyl-tetrahydro-pyran-3-ylamino)-3-(1H-indol-2-yl)-propionic acid

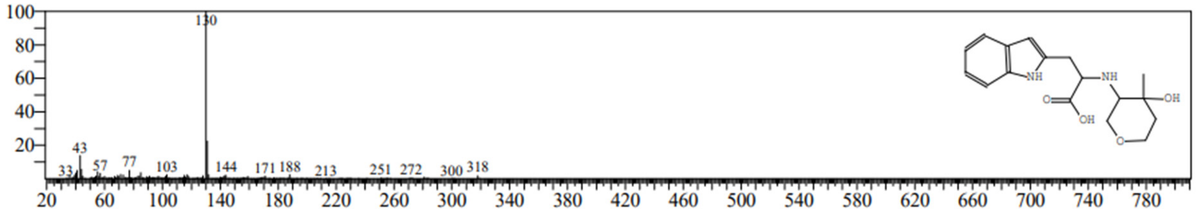

9. Compound name: Lycopene

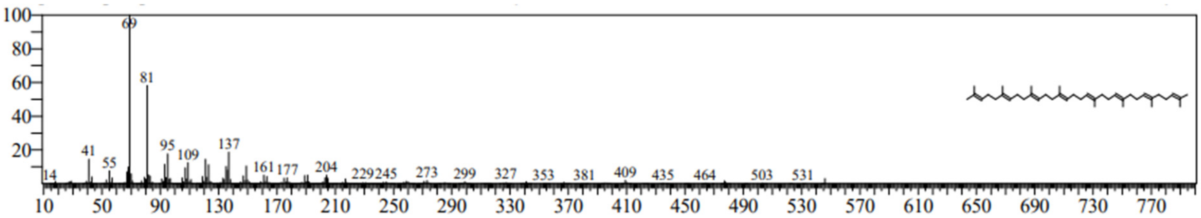

10. Compound name: Hexadecanoic acid, 2-hydroxy-1- (hydro oxymethyl) ethyl ester

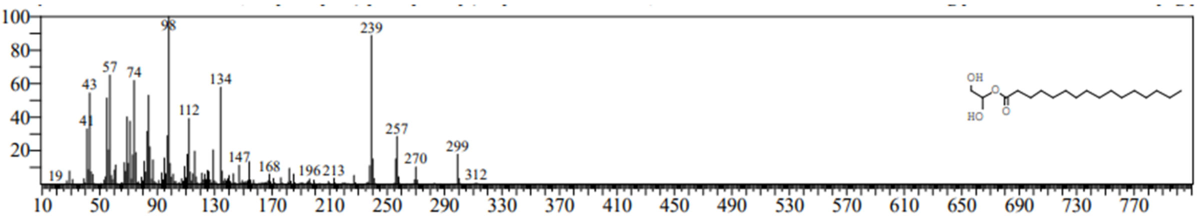

11. Compound name: Octocrylene 2-Propenoic acid

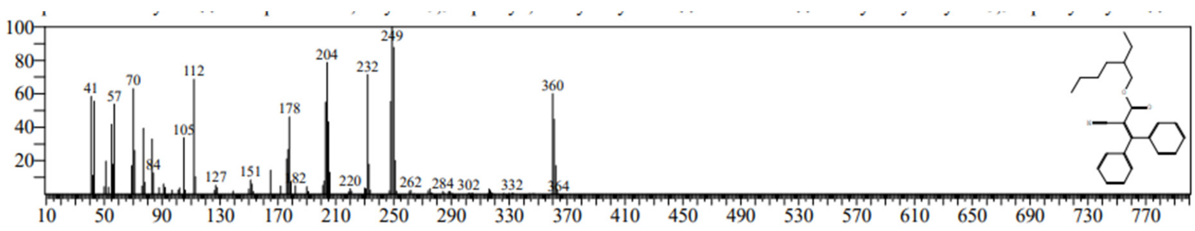

12. Compound name: Octadecanoic acid, 2,3-dihydroxypropyl ester

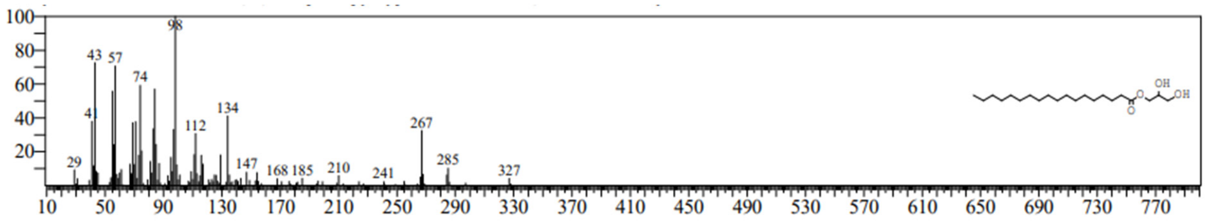

13. Compound name: Squalene e 2,6,10,14,18,22-Tetracosahexaene

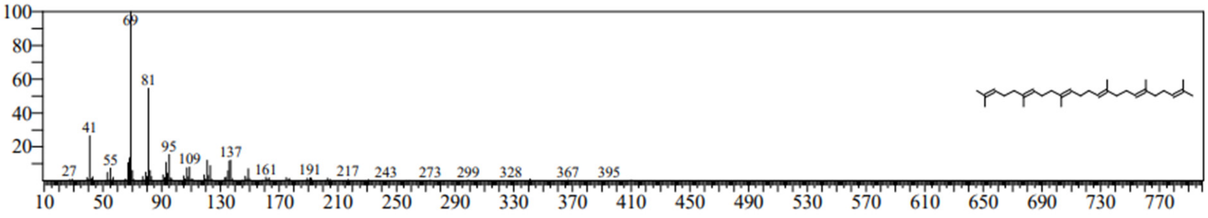

14. Compound name: Phenol, 2,4-bis (1,1-dimethyl ethyl)-, phosphite

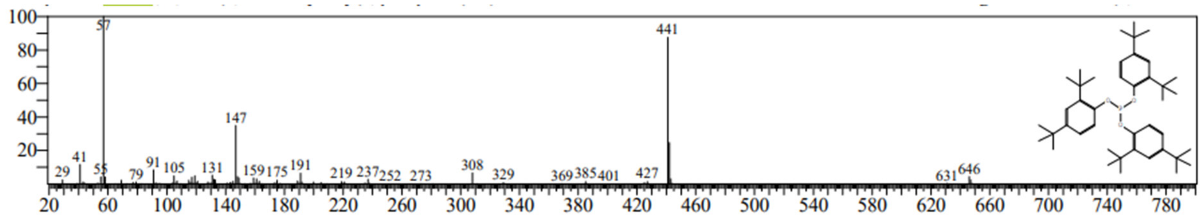

15. Compound name: Tris (2,4-di-tert-butyl phenyl) phosphate

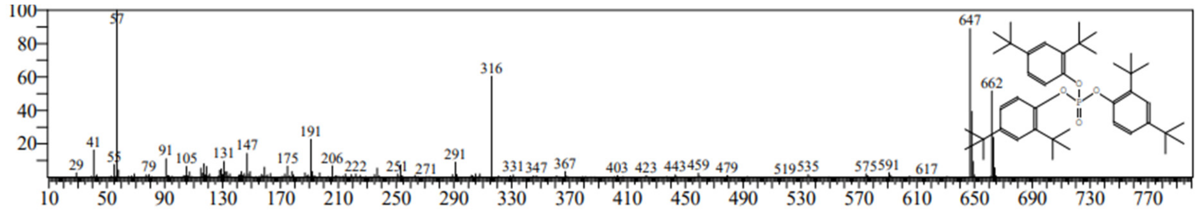

16. Compound name: Octahydro-2H-pyrido(1,2-a)pyrimidin-2-one

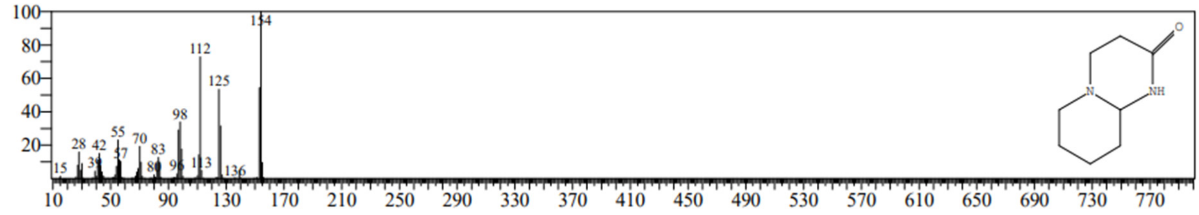

17. Compound name: Olean-18-ene

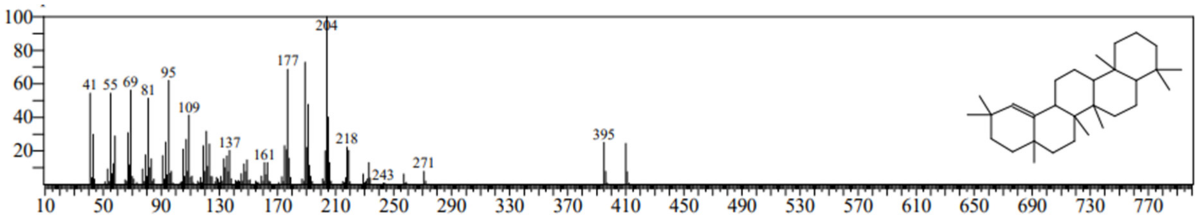

18. Compound name: l-Leucine

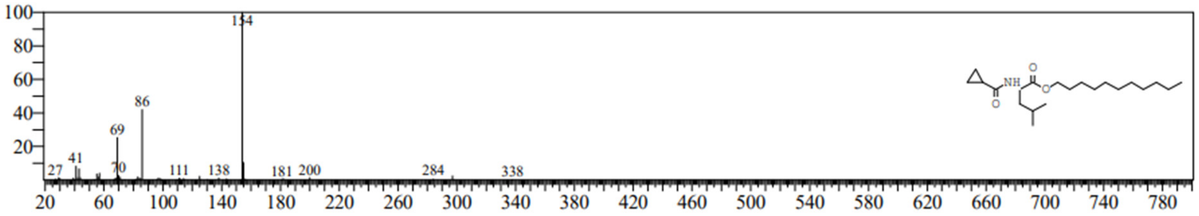

19. Compound name: L-Proline

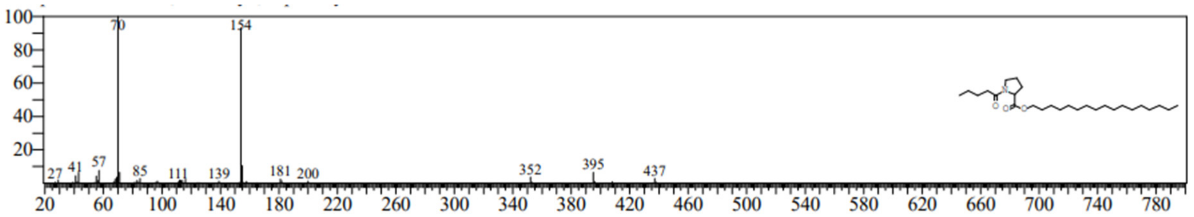

20. Compound name:Ergotaman-3',6',18-trione

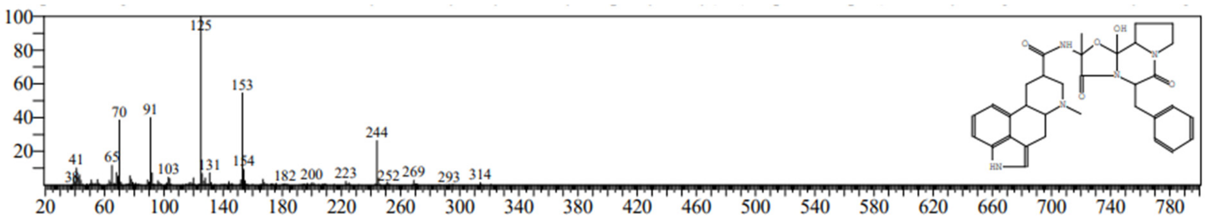

21. Compound name: Bis(2-ethylhexyl) phthalate

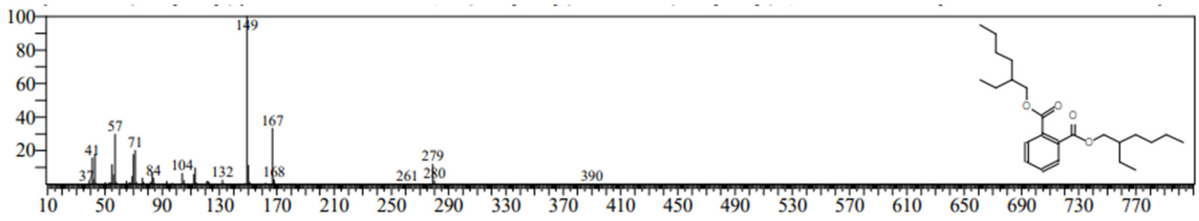

22. Compound name: 7,9-Di-tert-butyl-1-oxaspiro (4,5) deca-a-6,9-diene-2,8-dione

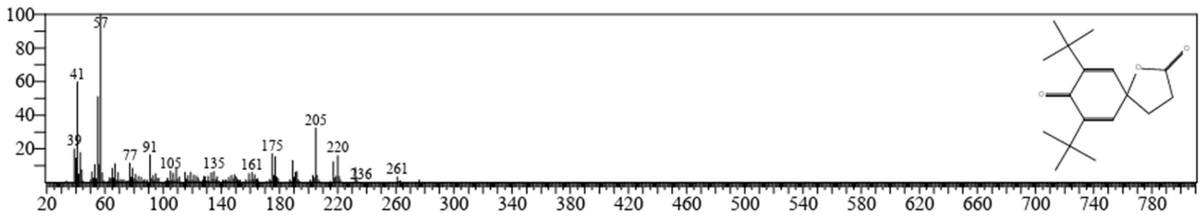

.

23. Compound name: 1,2-Benzenedicarboxylic acid, butyl octyl ester

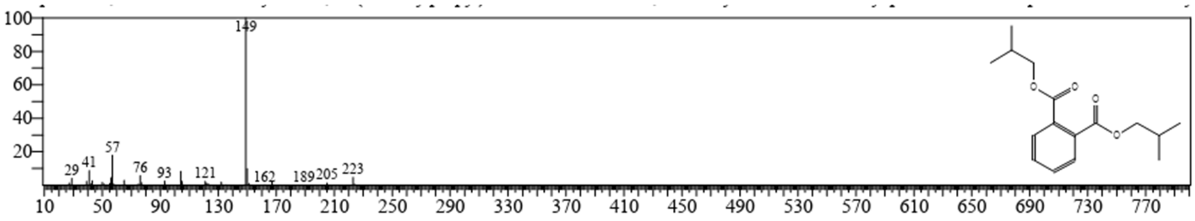

Mass fragmentation pattern and structural elucidations of unique metabolites secreted by *Serendipita indica* under normal growth conditions

1. Compound name: Dodecane,4,6-dimethyl

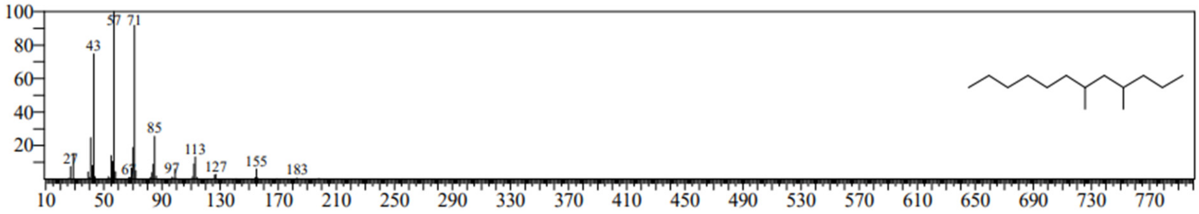

2. Compound name: Tetradecane

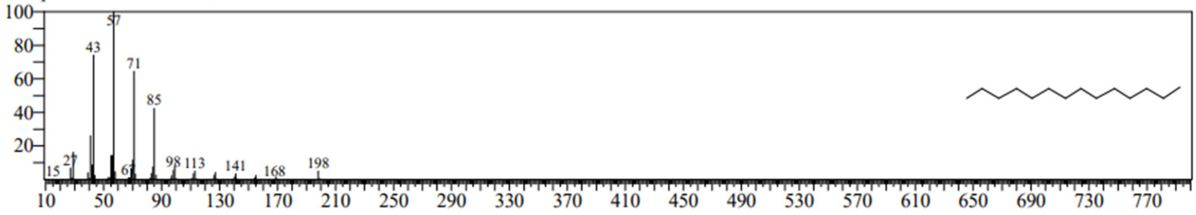

3. Compound name: Eicosane

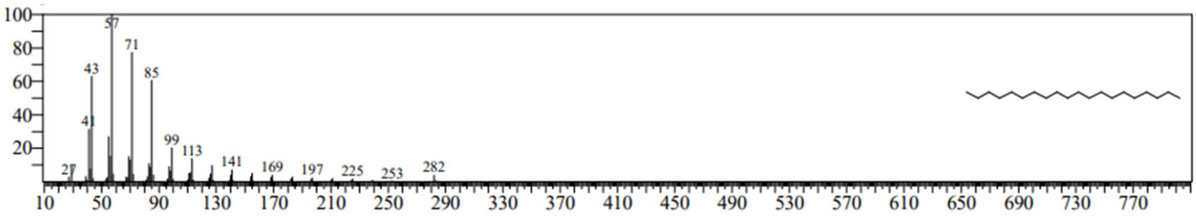

4. Compound name: 1-Hexadecanol

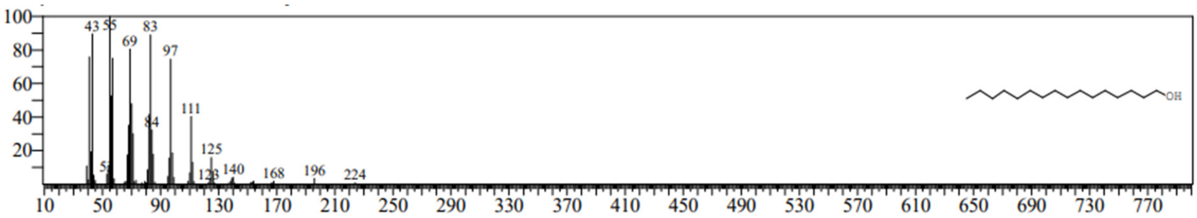

5. Compound name: Nonadecane

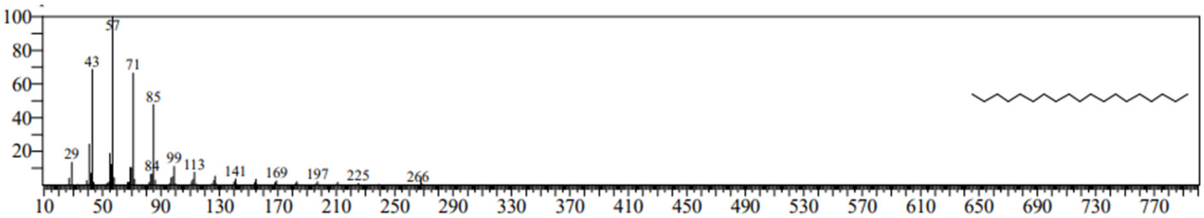

6. Compound name: Heneicosane

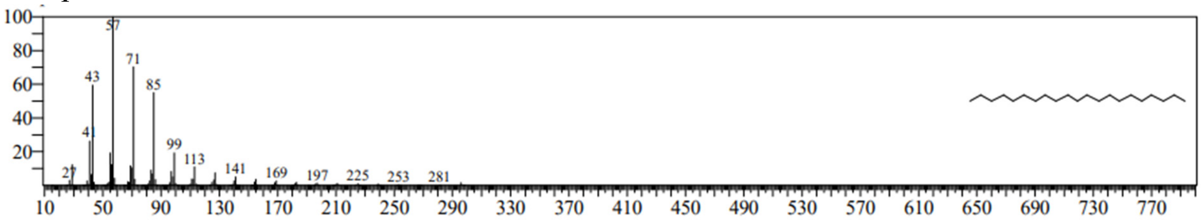

7. Compound name: 2-Propenoic acid, pentadecyl ester

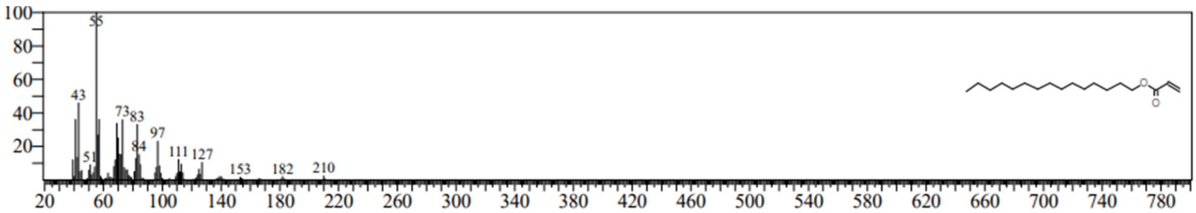

8. Compound name: Octadecane

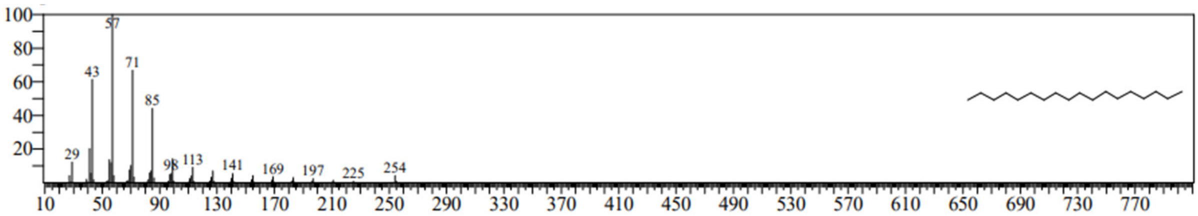

9. Compound name: Isopropyl myristate

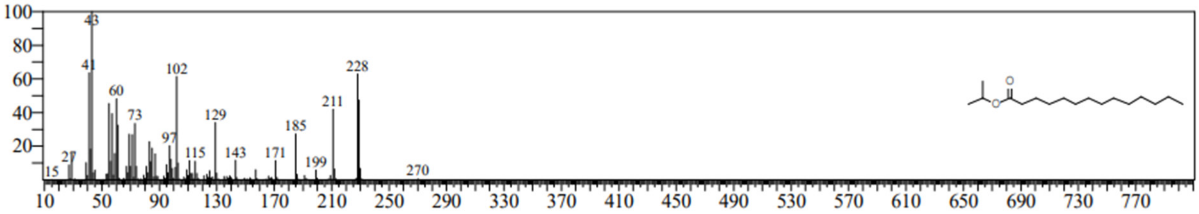

10. Compound name: Trifluoro acetoxy hexadecane

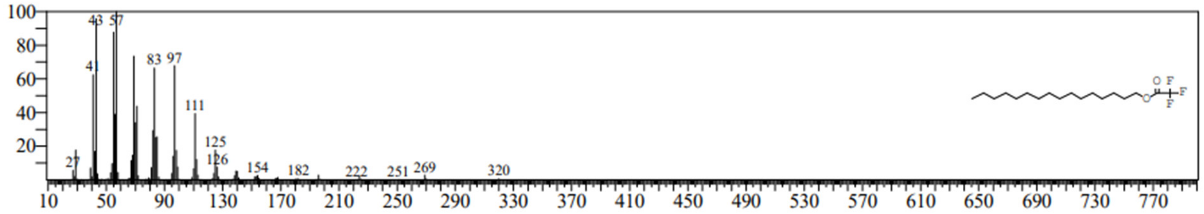

11. Compound name: Tetracosane

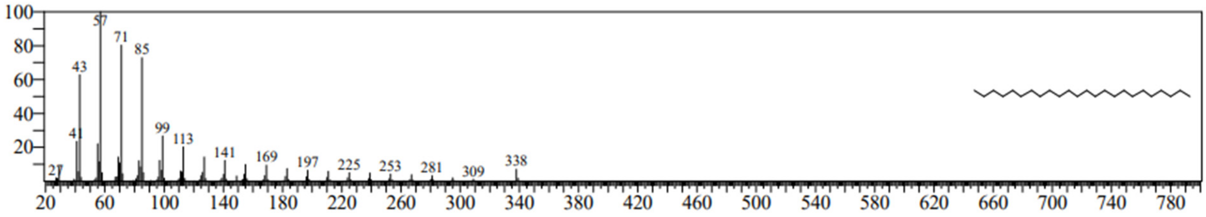

12. Compound name: Hexacosyl nonyl ether

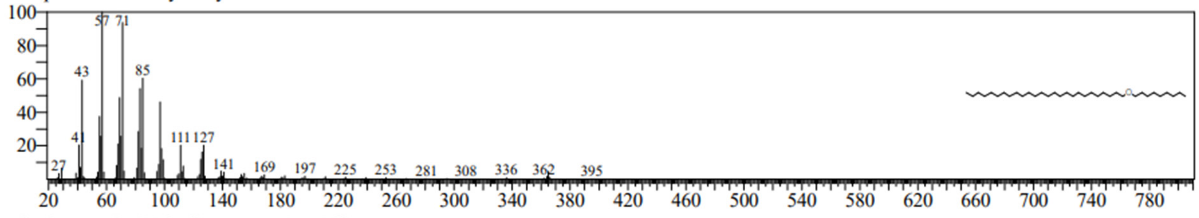

13. Compound name: Dibutyl phthalate

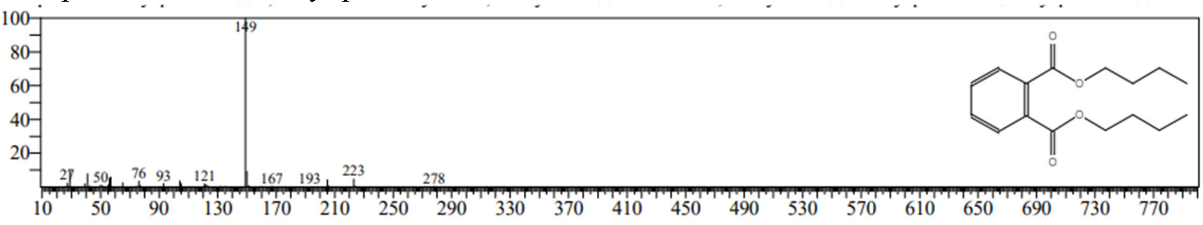

14. Compound name: l-(+)-Ascorbic acid 2,6-dihexadecanoate

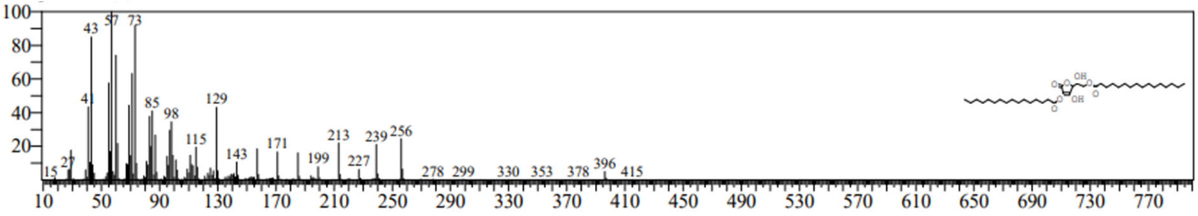

15. Compound name: Phthalic acid, 5-methylhex-yl butyl ester

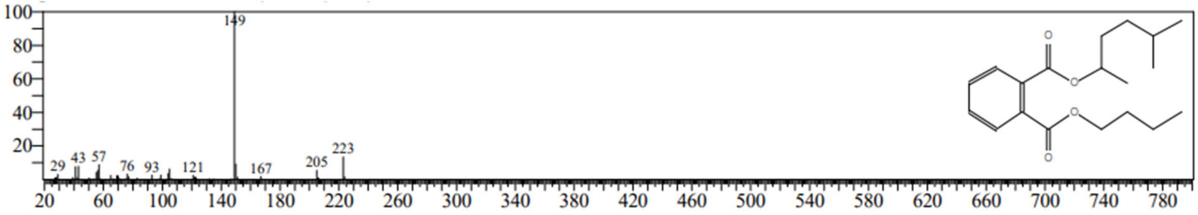

16. Compound name: Docosanoic acid, ethyl ester

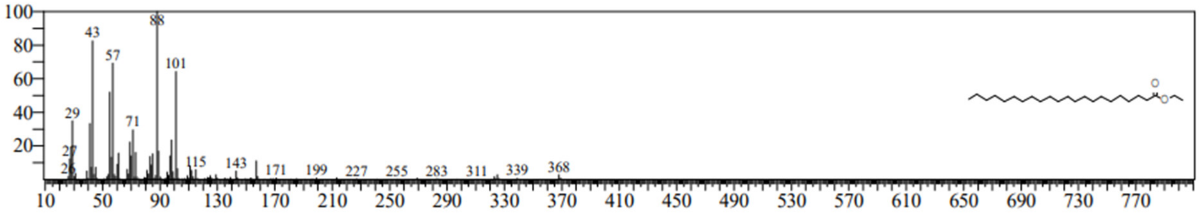

17. Compound name: Isopropyl palmitate

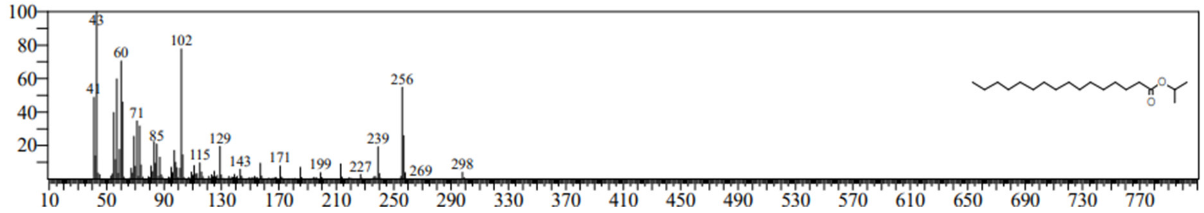

18 Compound name: n-Tetracosanol-1

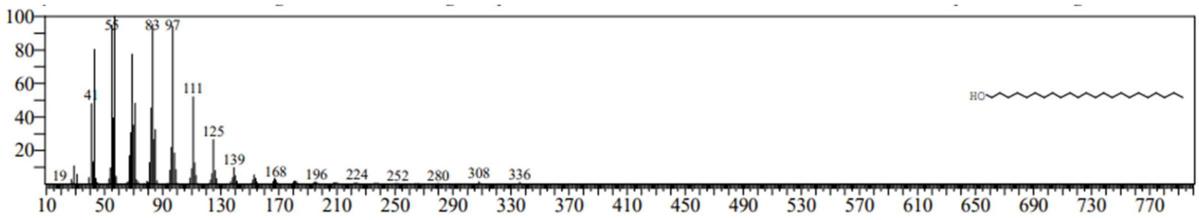

19. Compound name: 2-Methyltetracosane

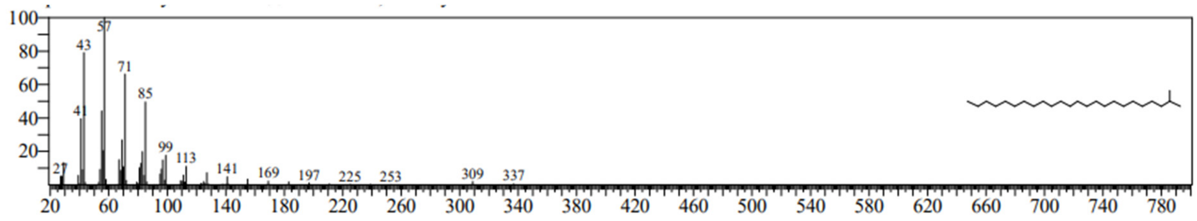

20 Compound name: Nonadecane nitrile

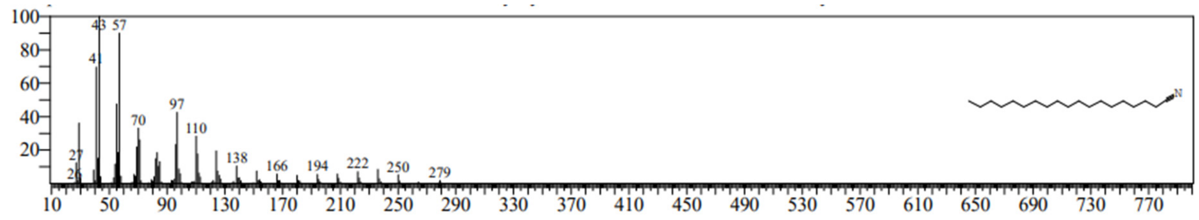

21 Compound name: Dodecane, 1,1-dimethoxy- Lauraldehyde

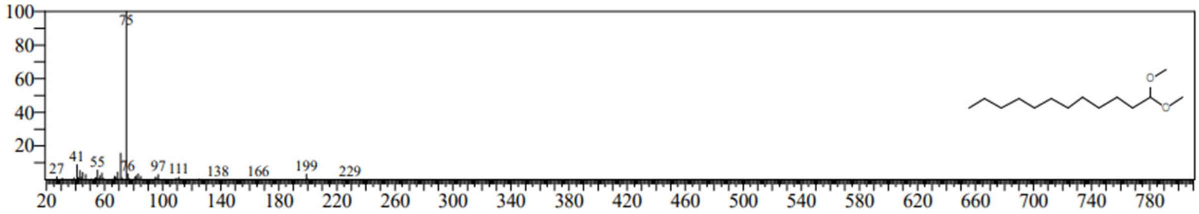

22 Compound name: Octadecanoic acid, 3-oxo-, ethyl ester

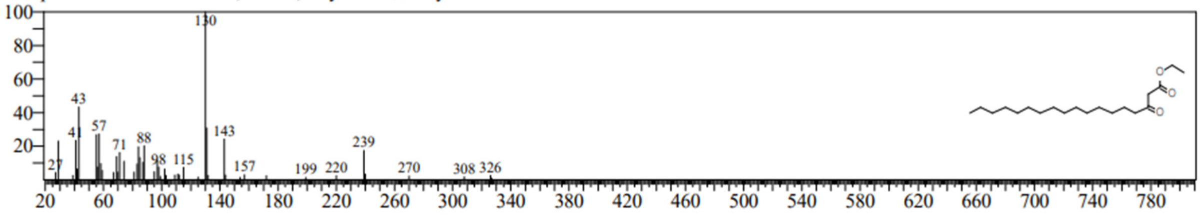

23. Compound name: 1,3-Benzenedicarboxylic acid, bis(2-ethylhexyl) ester

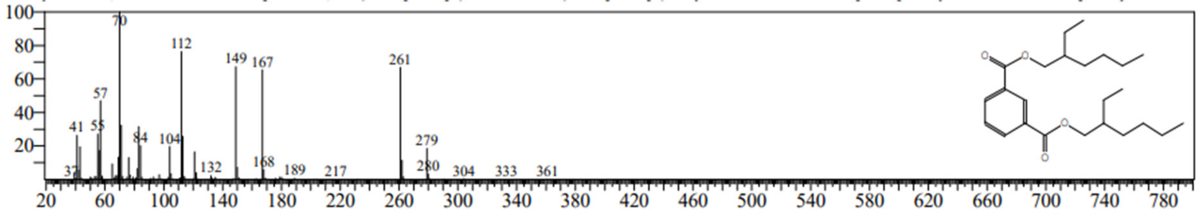

24. Compound name: 2-Methylhexacosane

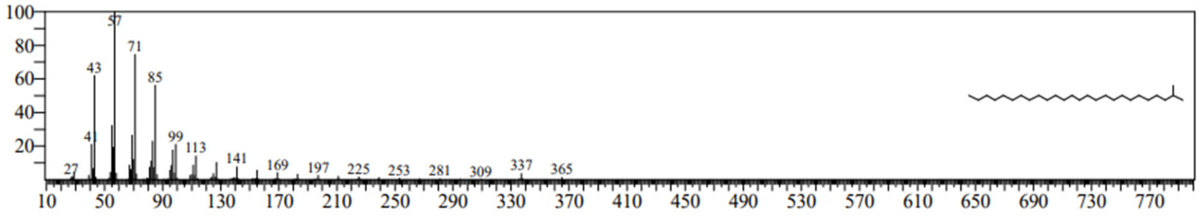

25. Compound name: 2-Isopropyl-5-methyl-1-heptanol

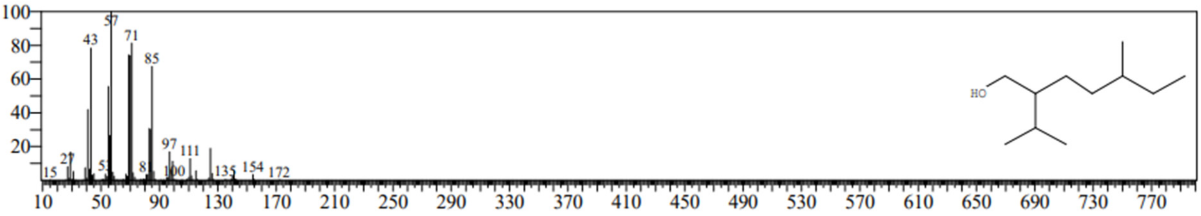

26. Compound name: Dodecane, 2,6,11-trimethyl

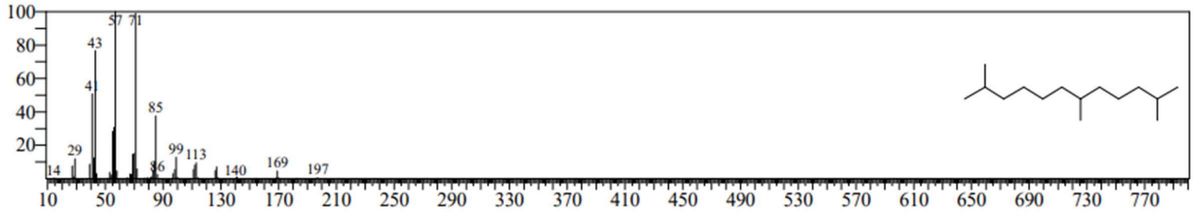

27. Compound name: 2,6,10-Trimethyltridecane

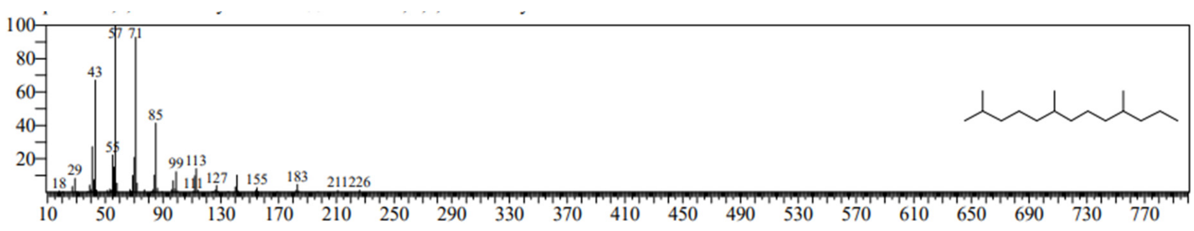

28. Compound name: Behenic alcohol

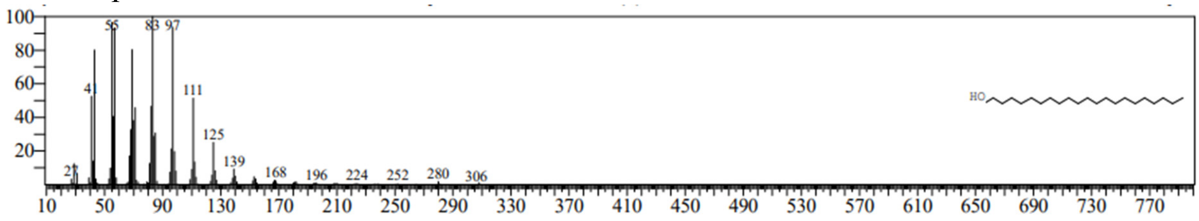

29. Compound name: N-2-ethylhexyl acetone imine

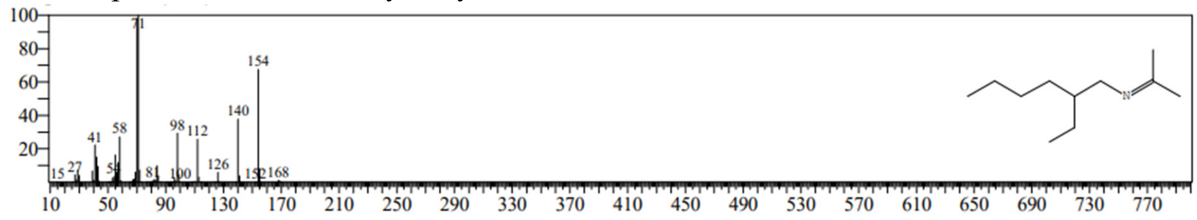

30. Compound name: N(1)-(3-Methyl-1,2,4-oxadiazol-5-yl)-1-pyrrolidinecarboxamidine

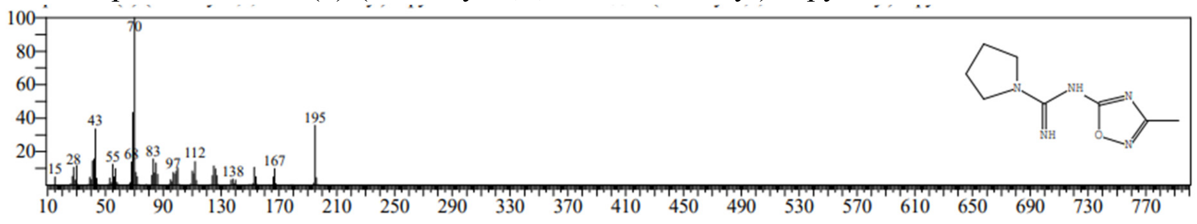

31. Compound name: l-(+)-Ascorbic acid 2,6-dihexadecanoate

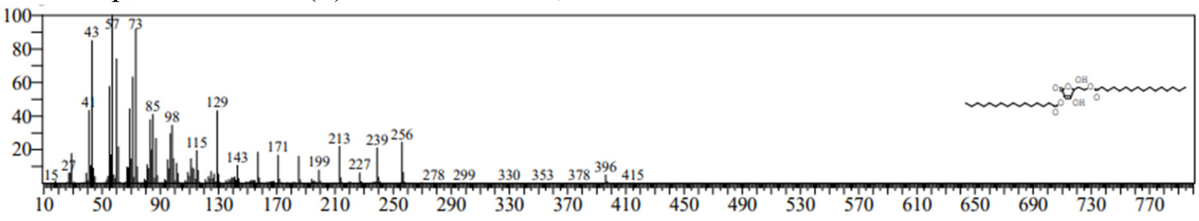

32. Compound name: Heptadecyl trifluoroacetate

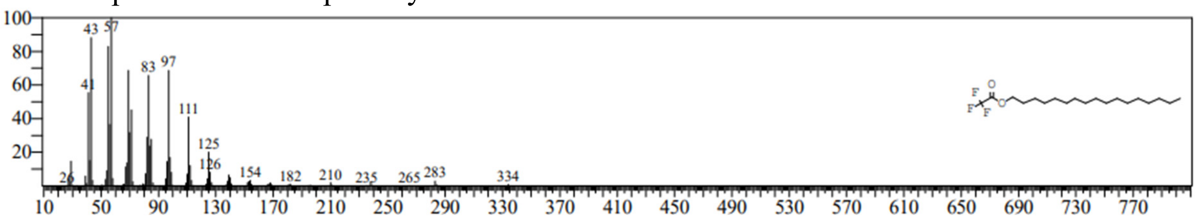

33. Compound name: 5,5-Diethylpentadecane

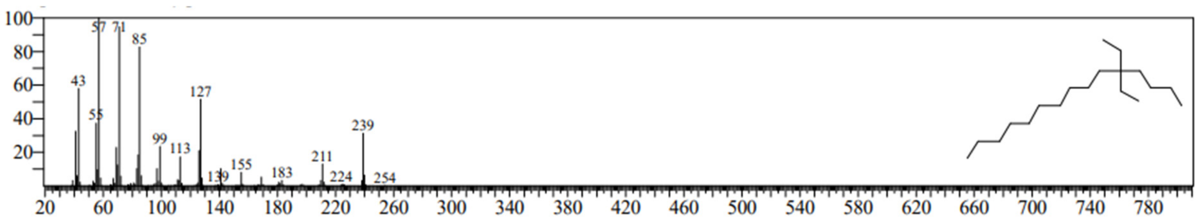

34. Compound name: Heptacos-1-ene

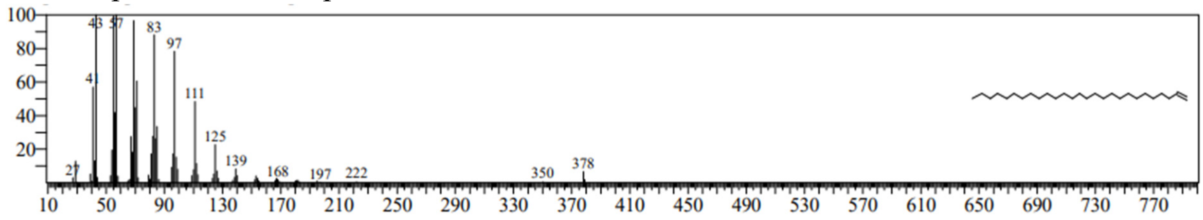

35. Compound name: 12,12-Dimethoxydodecanoic acid, methyl ester

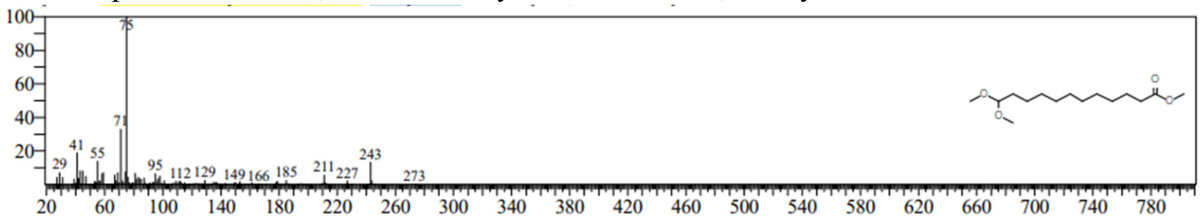

36. Compound name: Glycine

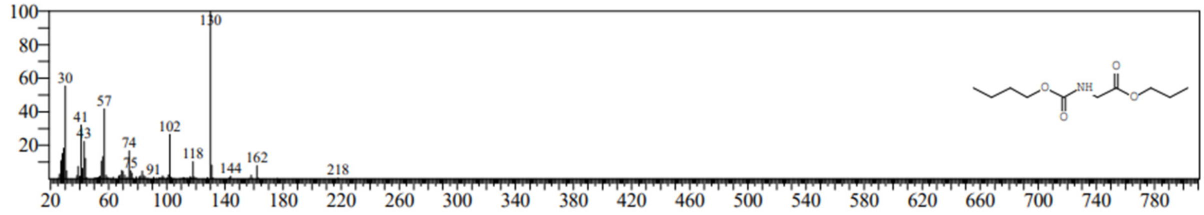

37. Compound name: Formamide, N-(4-[2-(1,1-dimethylethyl)-5-oxo-1,3-dioxolan-4-yl]butyl)

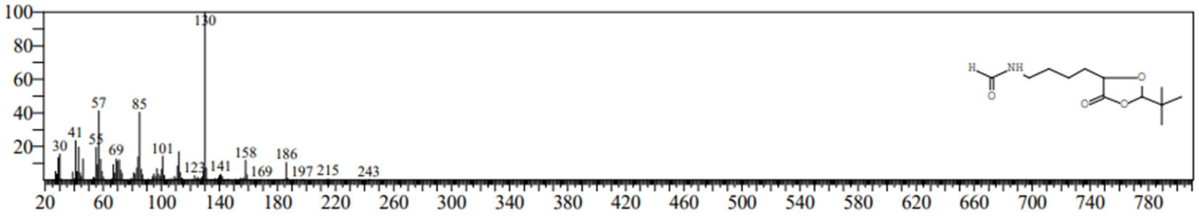

38. Compound name: Quinoline-4-carboxamide 2-phenyl-N-n.-octyl

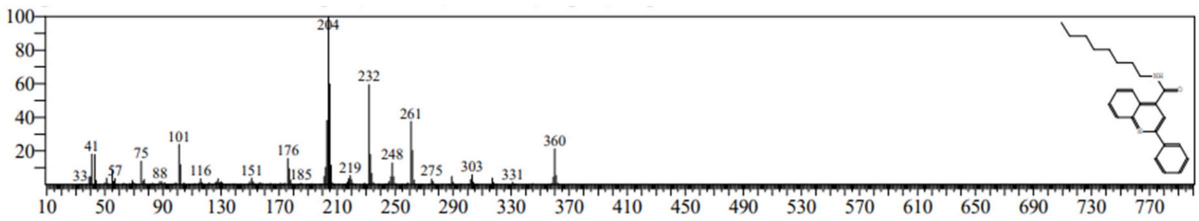

39. Compound name: Methanone, (3,5-dimethyl-1-piperidyl)(2-phenyl-4-quinolinyl)-

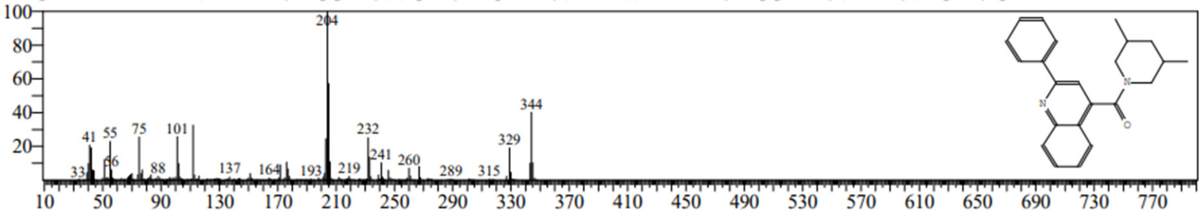

40. Compound name: Silane

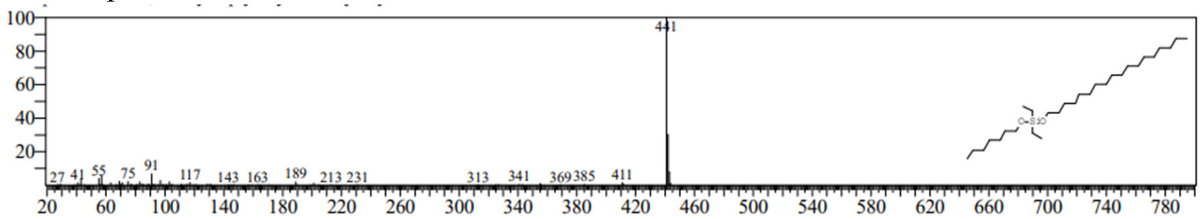

41. Compound name: Propionitrile 3-(3,5-di-tert-butyl-4-hydroxyphenyl)thio-

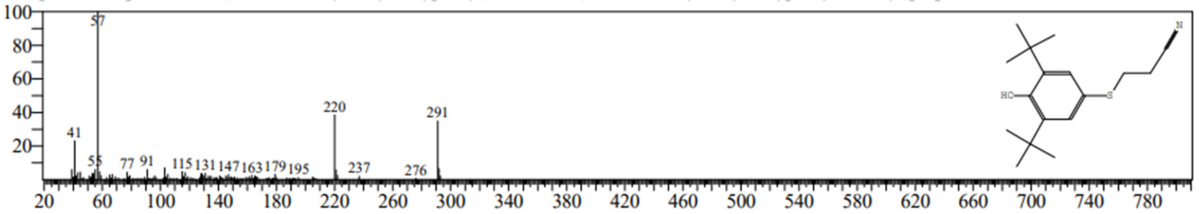

Mass fragmentation pattern and structural elucidations of unique metabolites secreted by *Serendipita indica* in presence of arsenic stress

1. Compound name: Tridecanoic acid, 12-methyl-, methyl ester

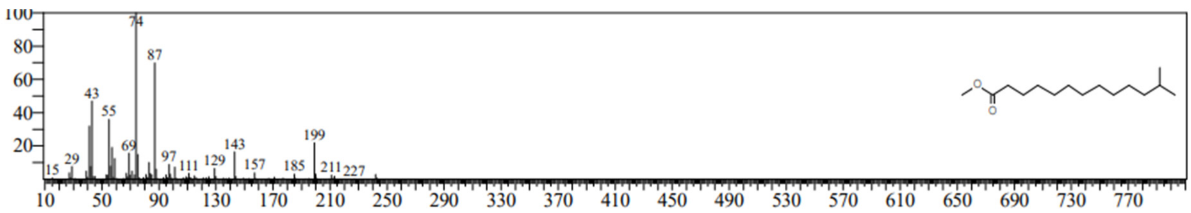

2. Compound name: n-Hexadecanoic acid

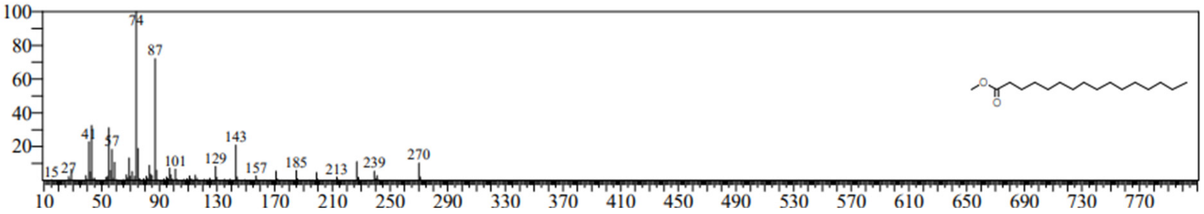

3. Compound name: 9-Octadecenoic acid, methyl ester, (E)- Elaidic acid

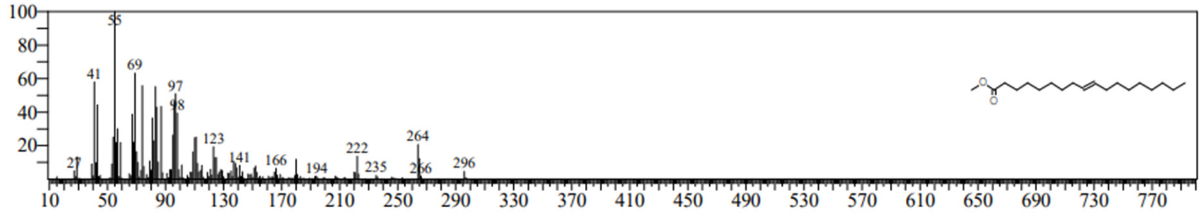

4. Compound name: Tetratriacontyl heptafluorobutyrate

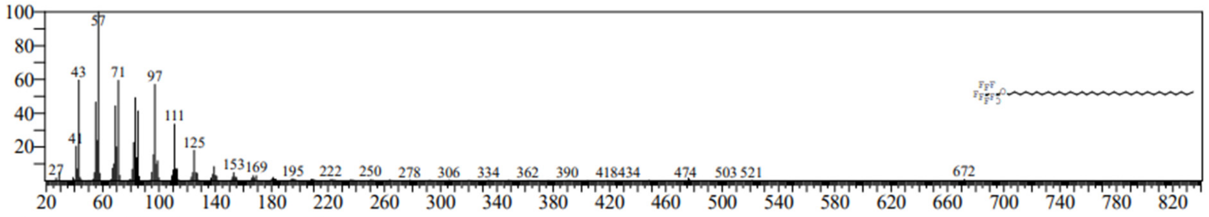

5. Compound name: Myristic acid, glycidyl ester Tetradecanoic acid

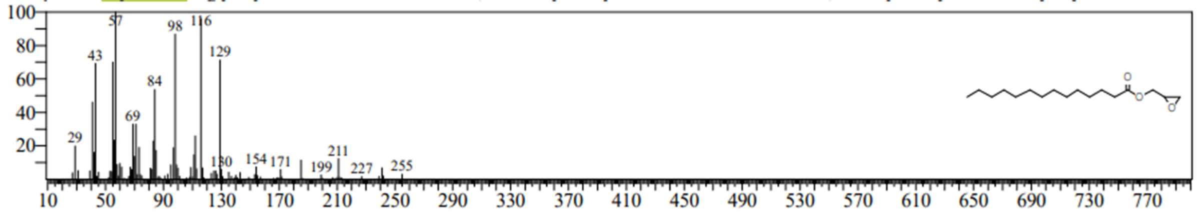

6. Compound name: 2-Propenoic acid, 3-(4-methoxyphenyl)

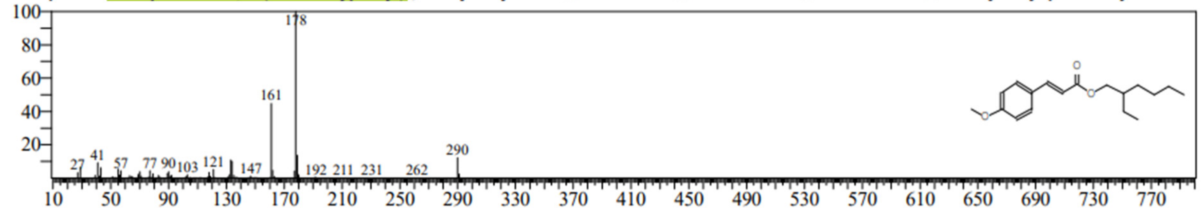

7. Compound name: 1,2,3,4-Tetrahydro-3- (phenyl acetamido) quinoline

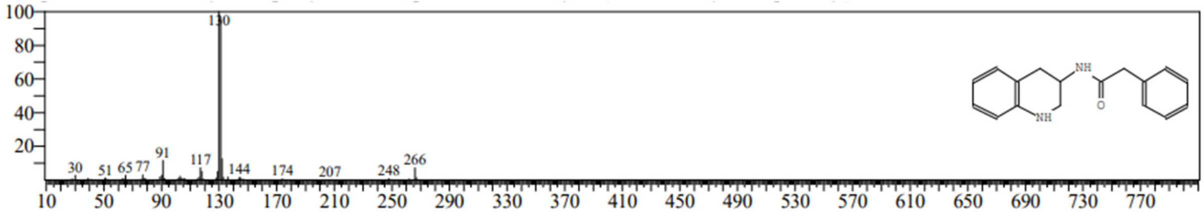

8. Compound name: 13-Docosenamide, (Z)- Erucylamide

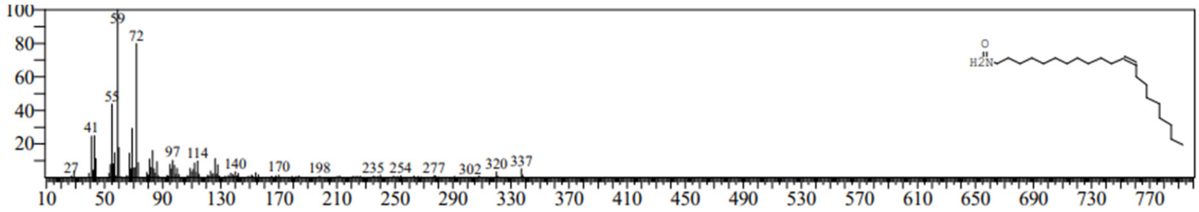

9. Compound name: Glycerol tri caprylate

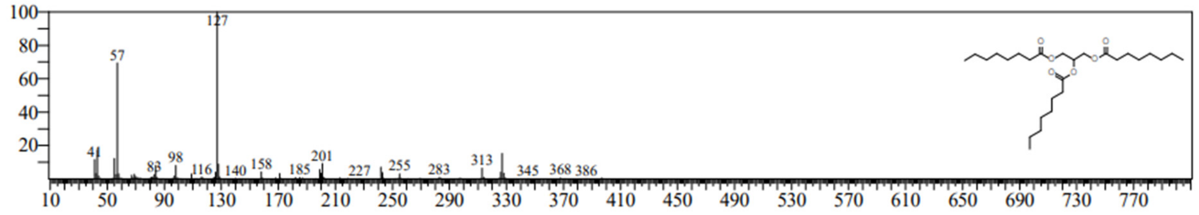

10. Compound name: 2-(Decanoyloxy) propane-1,3-diyl dioctanoate

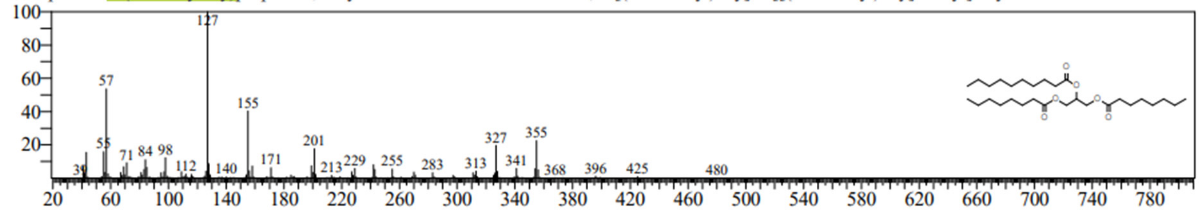

11. Compound name: Heptadecanoic acid, 16-methyl-, methyl ester

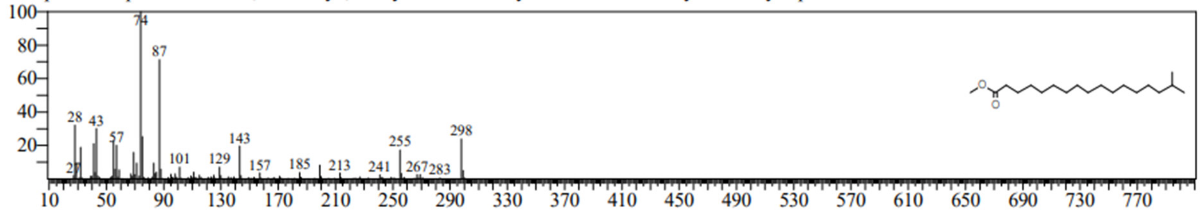

12. Compound name: Tetrapentacontane, 1,54-dibromo-

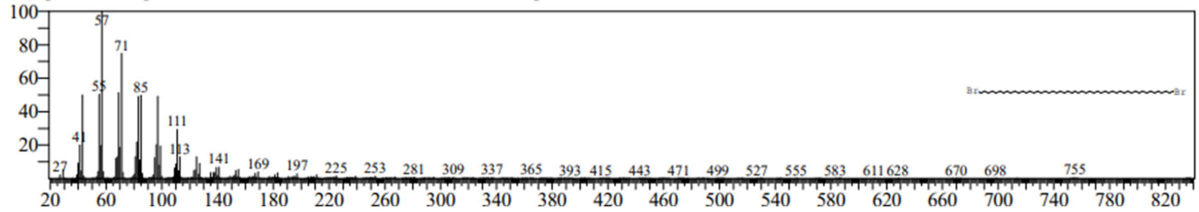

13. Compound name: Triacontyl heptafluorobutyrate

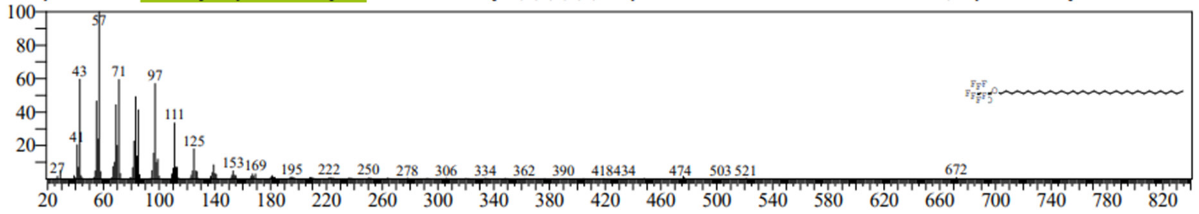

14. Compound name: Hexatriacontyl trifluoroacetate

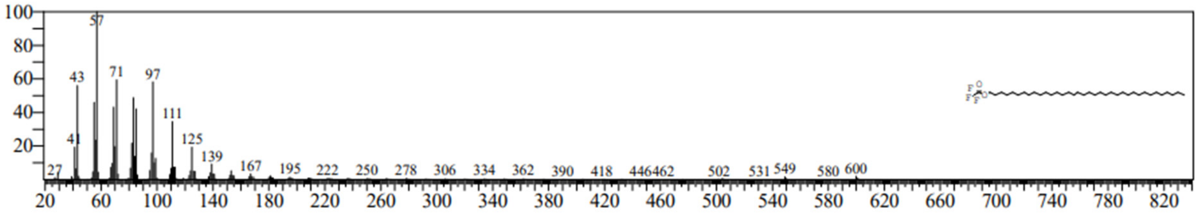

15. Compound name: 2-Ethylhexyl trans-4-methoxycinnamate

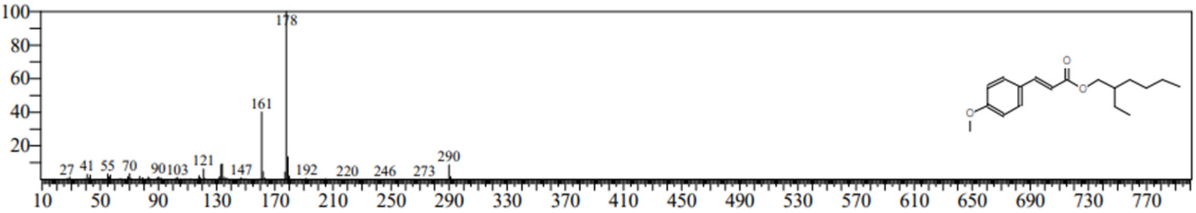

16. Compound name: 9-Octadecenamide

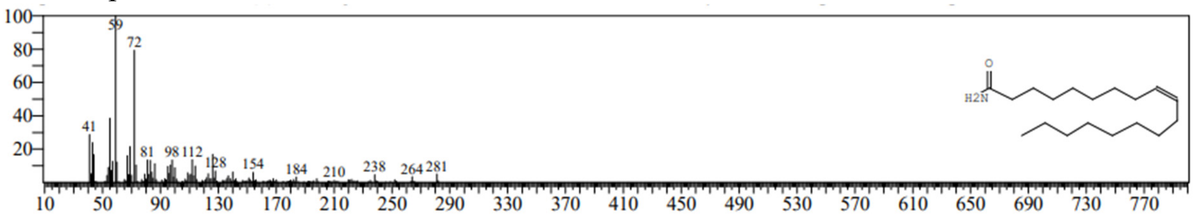

17. Compound name: 3-Indol-1-yl-propionic acid, methyl ester

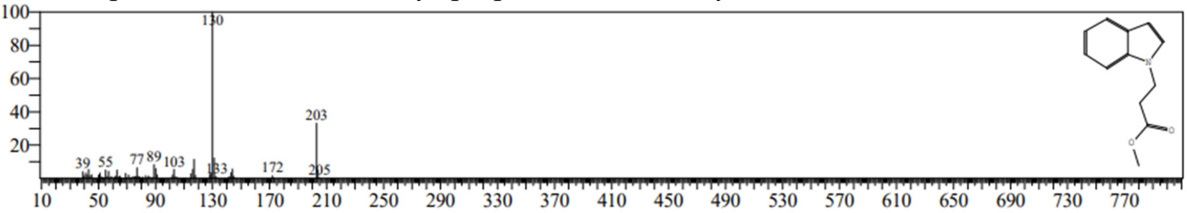

18. Compound name: 1,2,3,4-Tetrahydronaphthalen-1-yl 2,2,3,3,3-pentafluoropropanoate

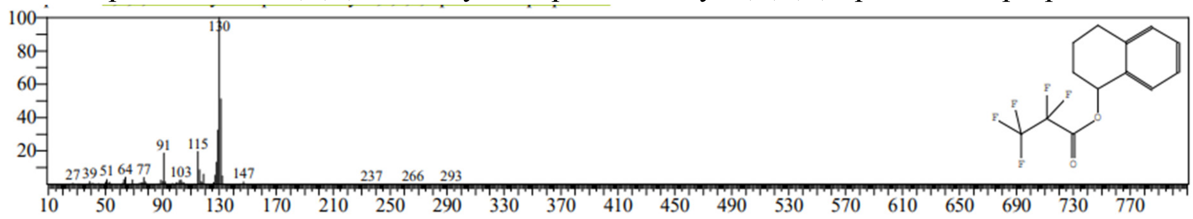

19. Compound name: L-Tryptophan,N-methyl-, methyl ester

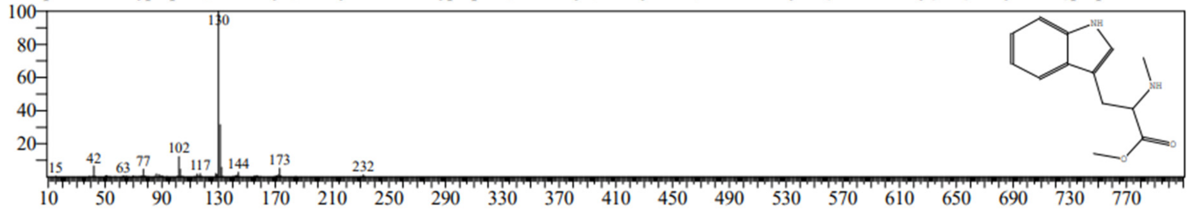

20. Compound name: 2-Ethylbutyric acid, eicosyl ester

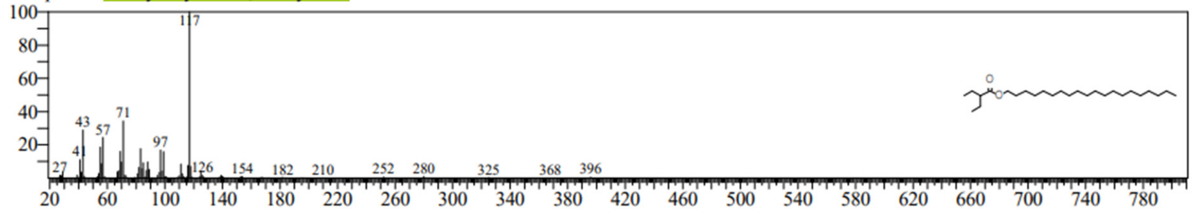

21. Compound name: Fumaric acid, 2,2,2-trichloroethyl tridecyl ester

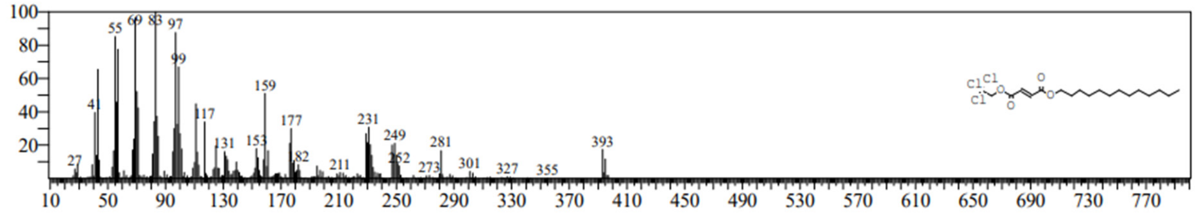

22. Compound name: Cyclooctadecane

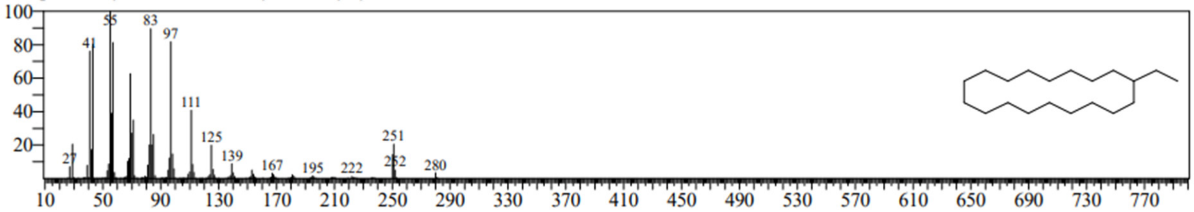

23. Compound name: 2,6,10,14,18-Pentamethyl-2,6,10,14,18-eicosapentaene

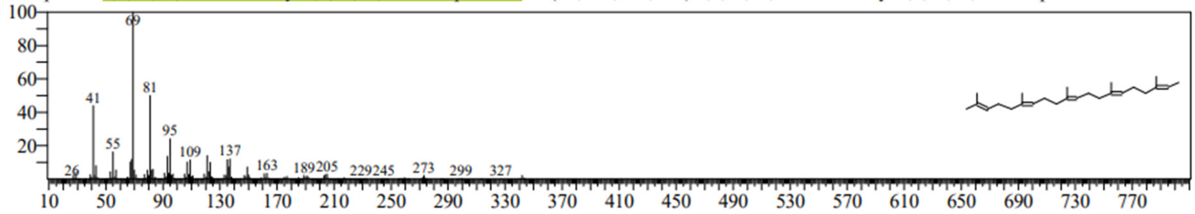

24. Compound name: 5,9,13,17-Tetramethyl 4,8,12,16-octadecatetraenoic acid

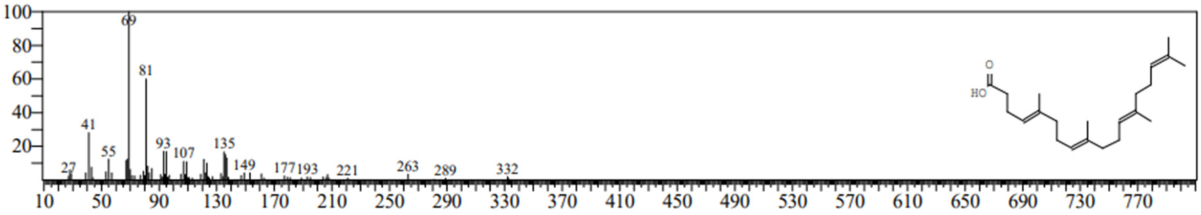

25. Compound name: 3-(Octanoyloxy)propane-1,2-diyl bis(decanoate)

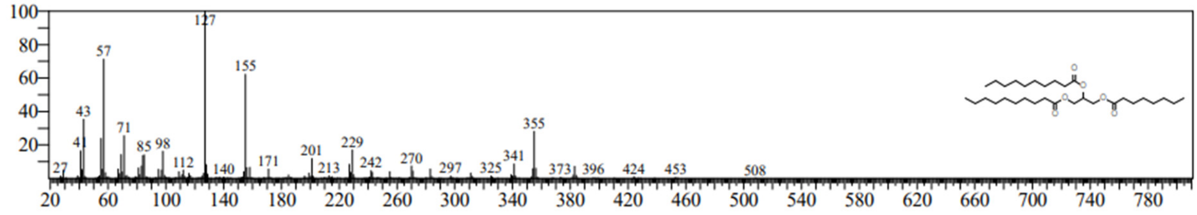

26. Compound name: Pentacyclo[19.3.1.1(3,7).1(9,13).1(15,19)]octacosa-1(25),3,5,7(28),9,11,13(27),15,17,19(26),21,23-dodecaene-25,26,27,28-tetrol, 5,11,17,23-

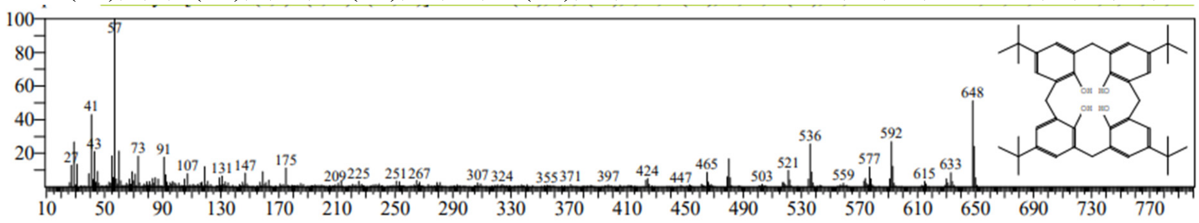

Supplementary Material S2

Mass fragmentation patterns and structural elucidations of common secondary metabolites secreted by *Serendipita indica* and *Zhihengliuella* sp. ISTPL4 under normal growth conditions and in the presence of arsenic stress

A total of 67 metabolites were produced by a combination of *S. indica* and *Z.* sp. ISTP4 under normal conditions and 37 metabolites were produced in As stress (out of which 16 metabolites were common)

1. Compound name: 2,4-Di-tert-butyl-phenol) phosphate

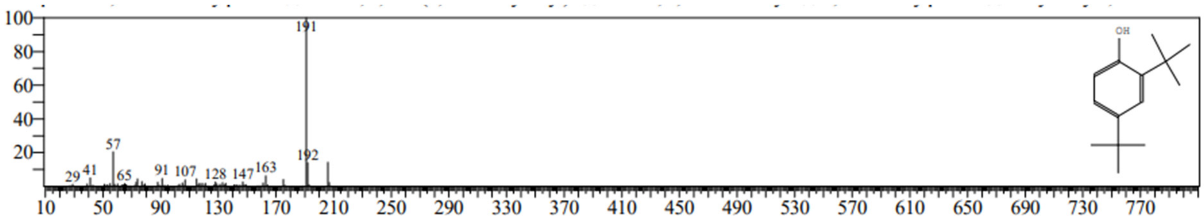

2. Compound name: Cyclo(L-prolyl-L-valine)

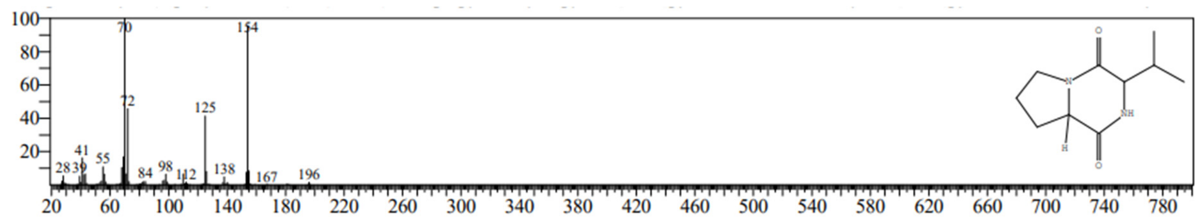

3. Compound name: 1,2-Benzenedicarboxylic acid, bis (2-methyl propyl) ester

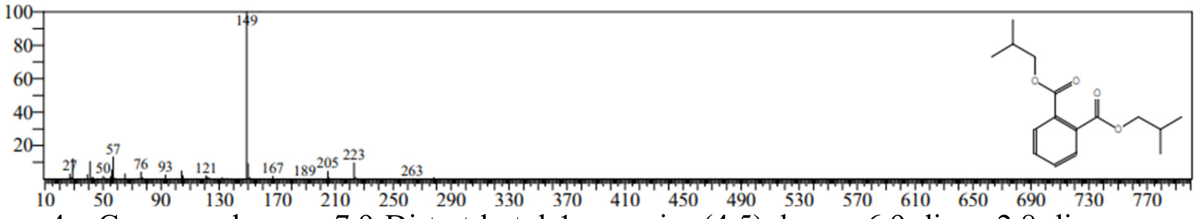

4. Compound name: 7,9-Di-tert-butyl-1-oxaspiro (4,5) deca-a-6,9-diene-2,8-dione

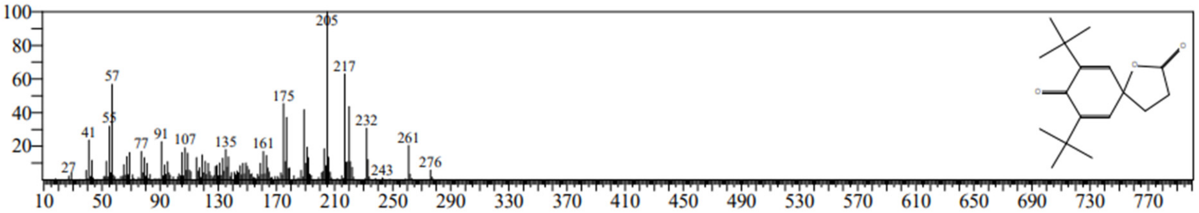

5. Compound name: Hexadecenoic acid, methyl ester

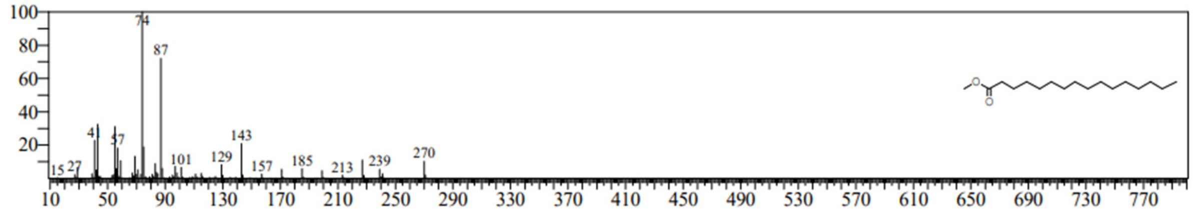

6. Compound name: octadecanoic acid, 3-oxo-, ethyl ester

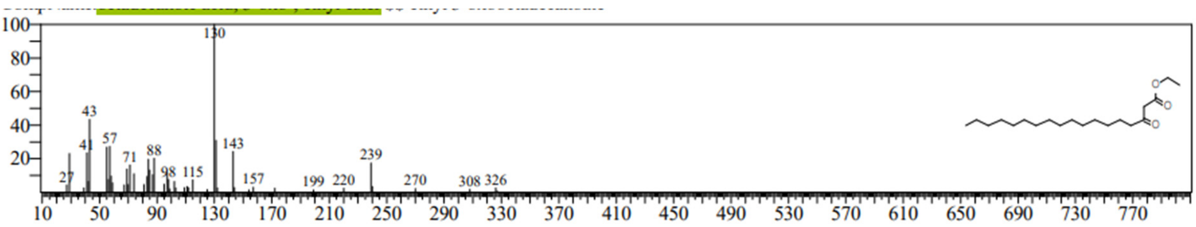

7. Compound name: Olean-18-ene

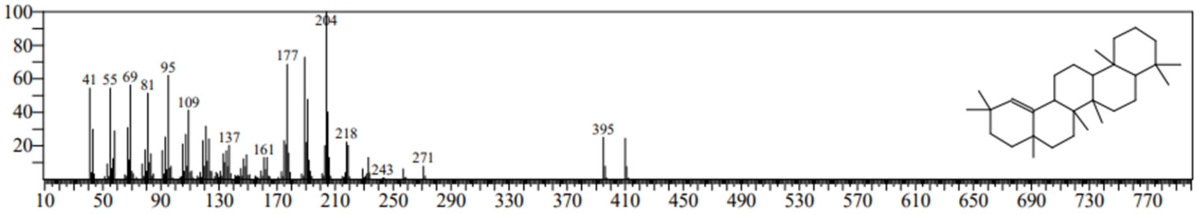

8. Compound name: L-Proline

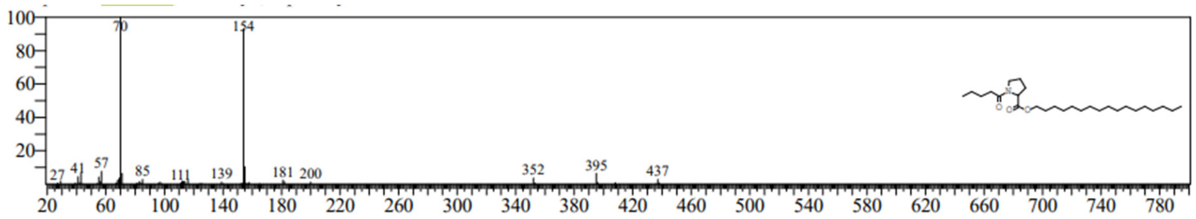

9. Compound name: Glycerol 1-palmitate

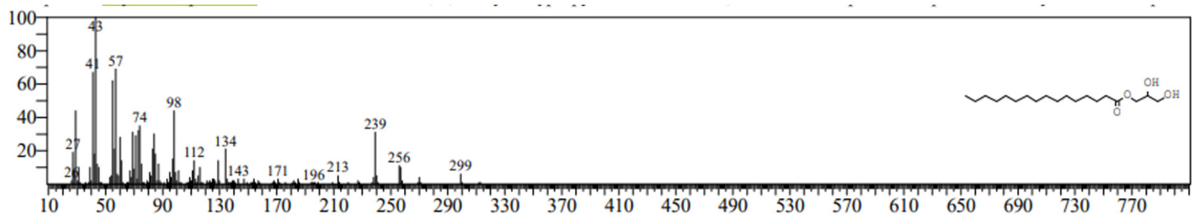

10. Compound name: Hexadecanoic acid, 2-hydroxy-1-(hydroxymethyl)ethyl ester

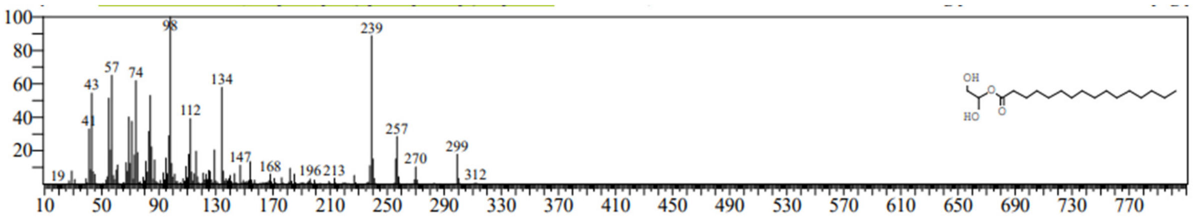

11. Compound name: Octadecanoic acid, 2,3-dihydroxypropy ester

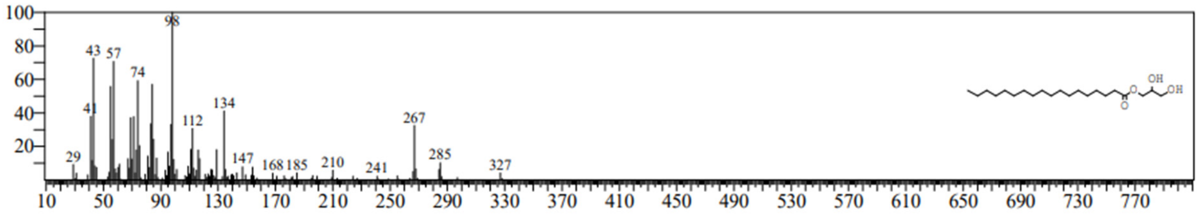

12. Compound name: Methyl stearate

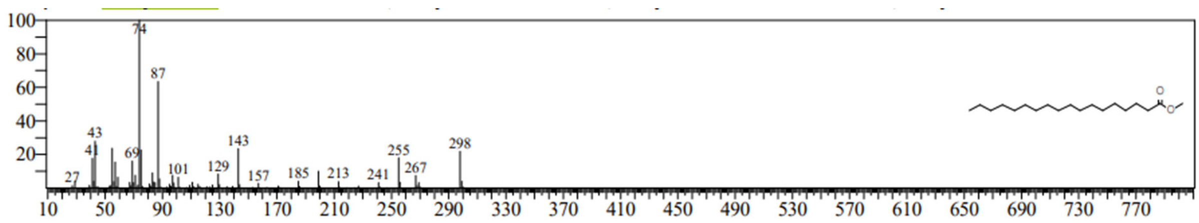

13. Compound name: Pyrrole[1,2-a] pyrazine-1,4-dione, hexahyd dro-3-(2-methyl propyl)

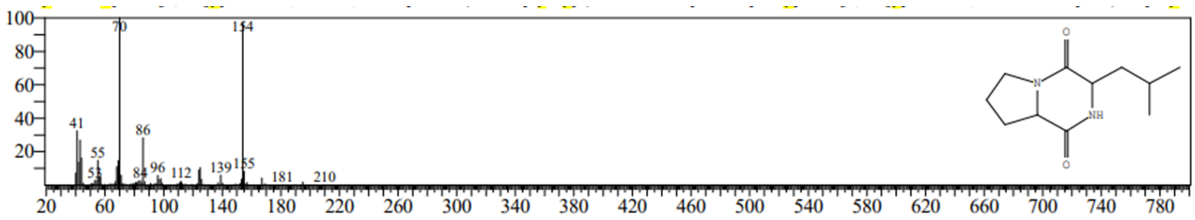

14. Compound name: Eicosane

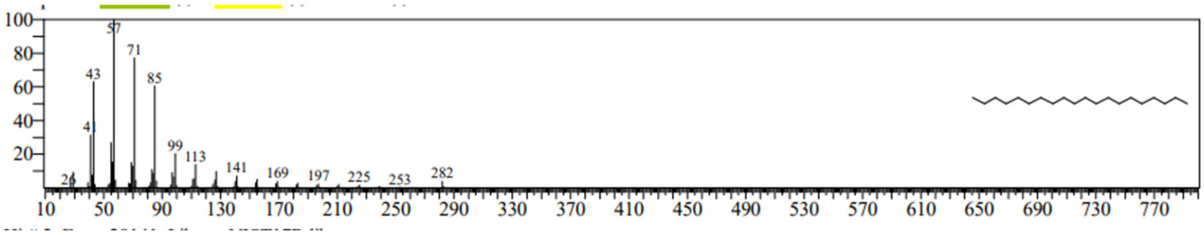

15. Compound name: Heneicosane

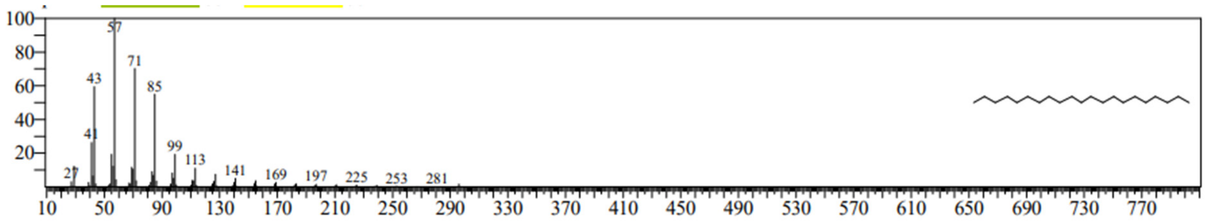

16. Compound name: Tetradecane

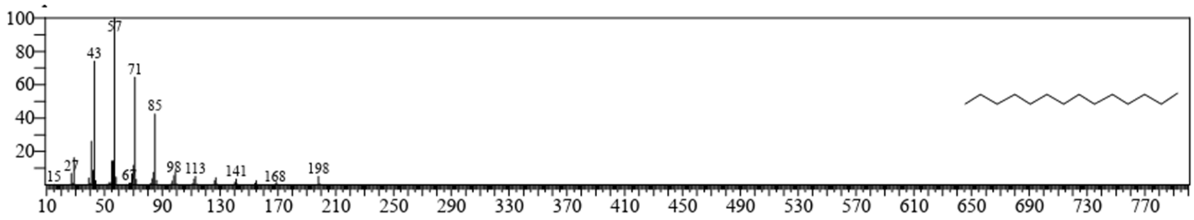

Mass fragmentation patterns and structural elucidations of unique secondary metabolites secreted by *Serendipita indica* and *Zhihengliuella* sp. ISTPL4 under normal growth conditions

1. Compound name: Benzene, 1,3-bis(1,1-dimethylethyl)-

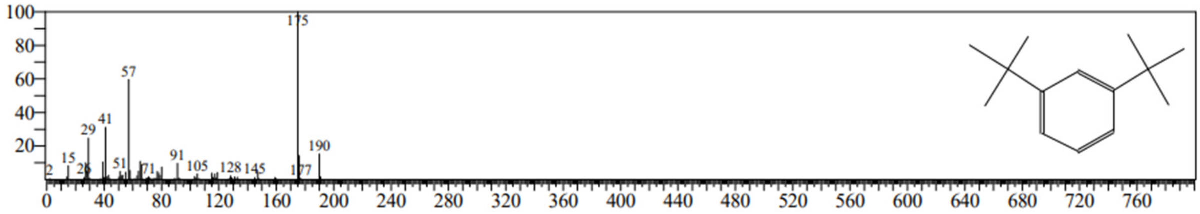

2. Compound name: Dodecane,4,6-dimethyl

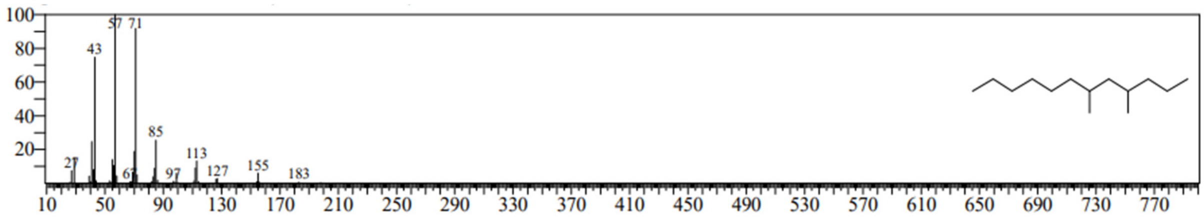

3. Compound name: Hexadecane

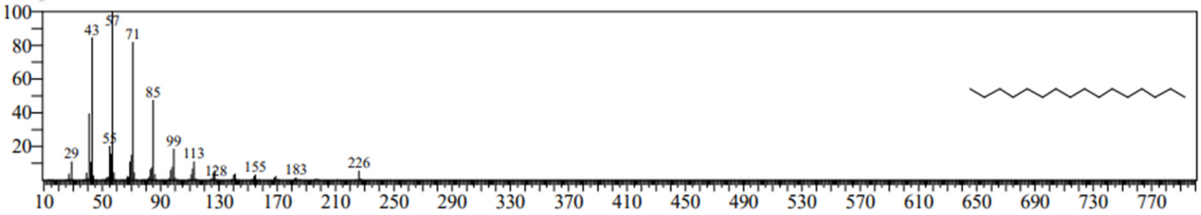

4. Compound name: Tetradecane

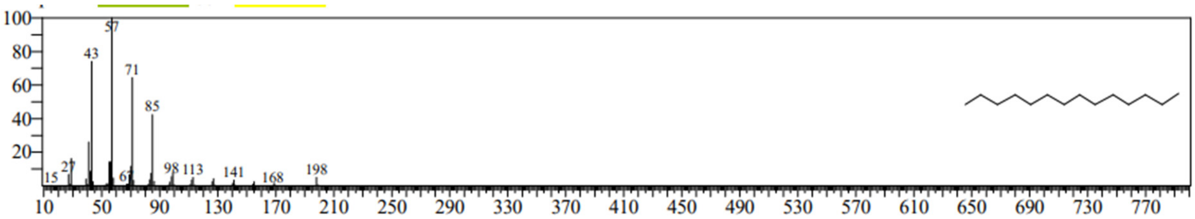

5. Compound name: Heptadecane

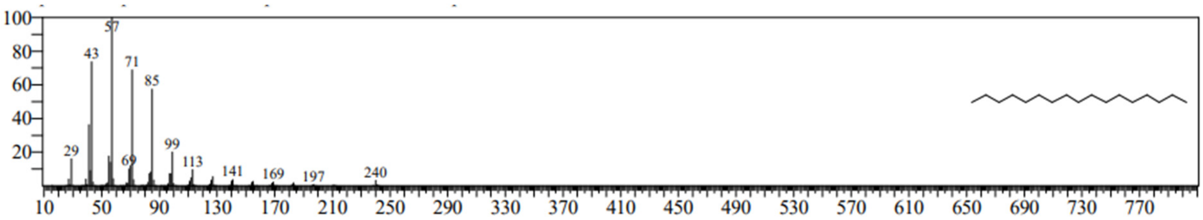

6. Compound name: I-Nonadecene

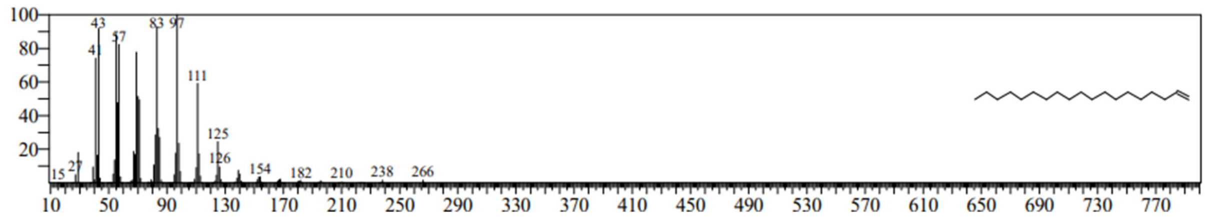

7. Compound name: Diethylpent-4-enylamine

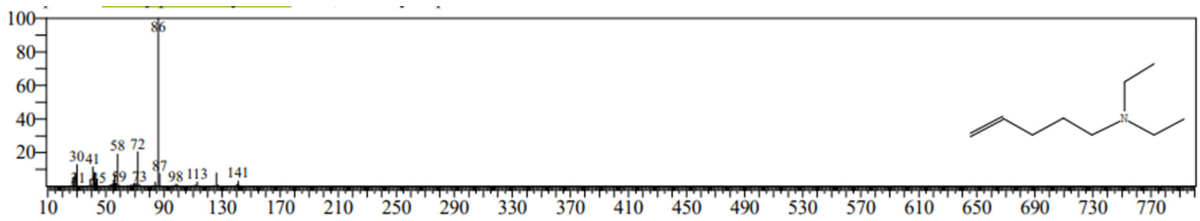

8. Compound name: 3,6-Diisopropylpiperazin-2,5-dione

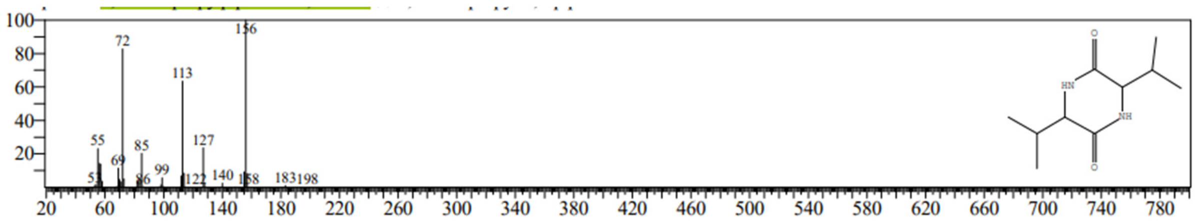

9. Compound name: Tetracosane

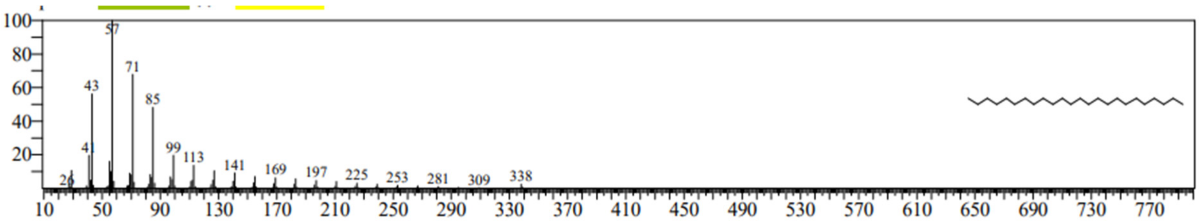

10. Compound name: 6,6-Diethylhoctadecane

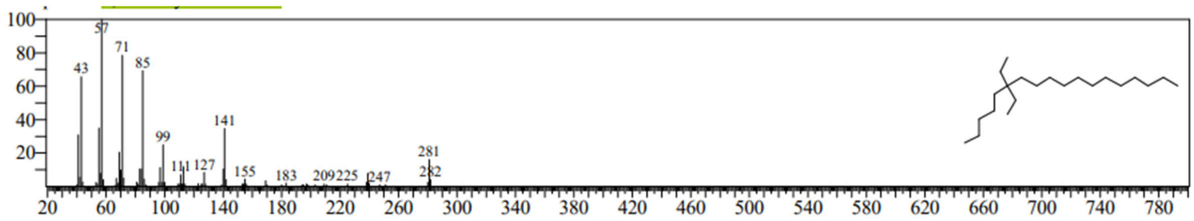

11. Compound name: n-Hexadecanoic acid methyl ester

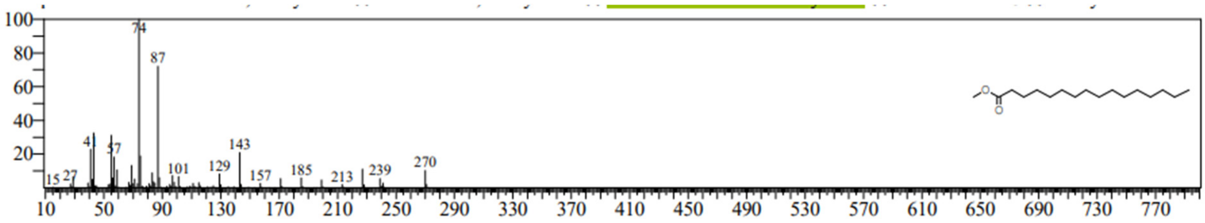

12. Compound name: 2-Methylhexacosane

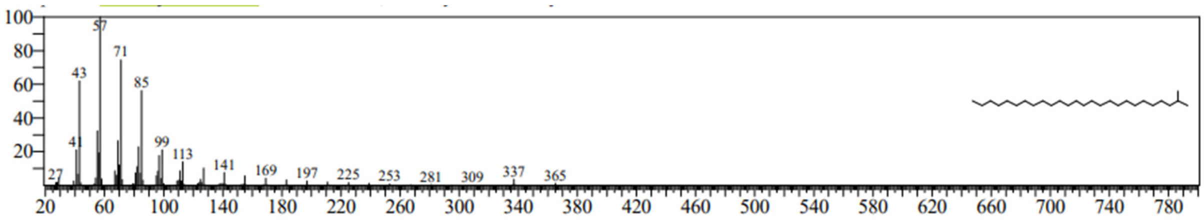

13. Compound name: Propionamide, N-propyl-N-decyl

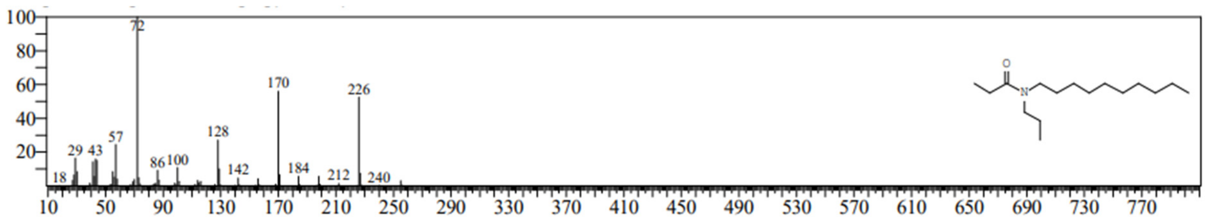

14. Compound name: Heneicosane, 10-methyl-

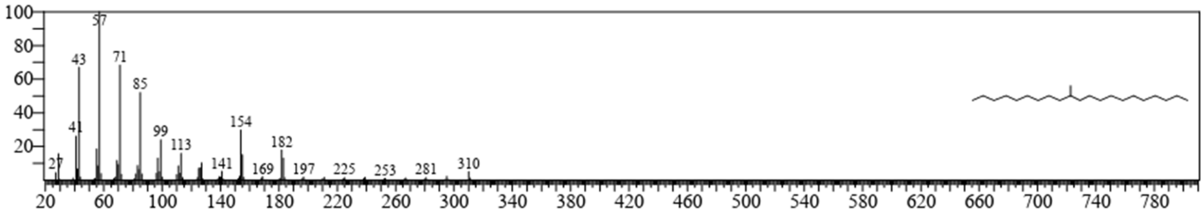

15. Compound name: Hexanoic acid, heptadecyl ester

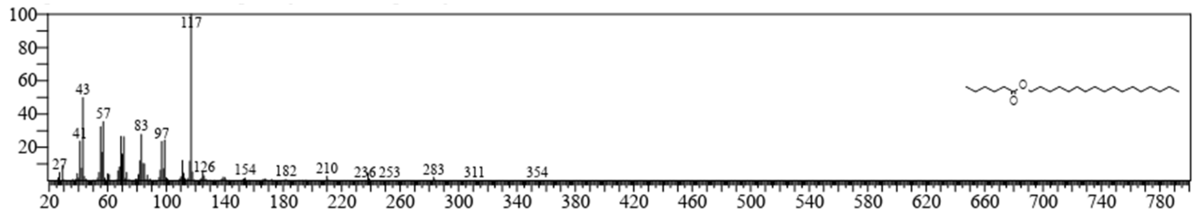

16. Compound name: Tetrapentacontane

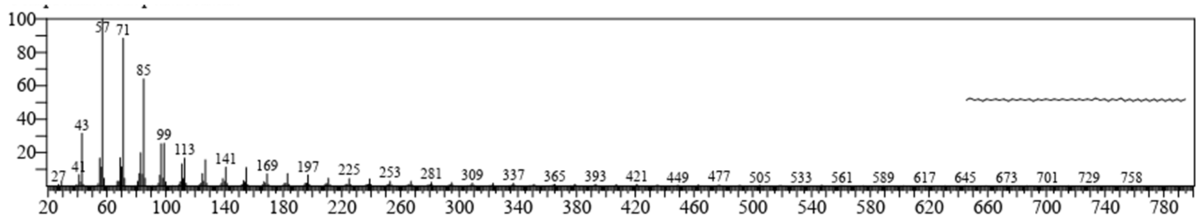

17. Compound name: 2-Methyltetracosane

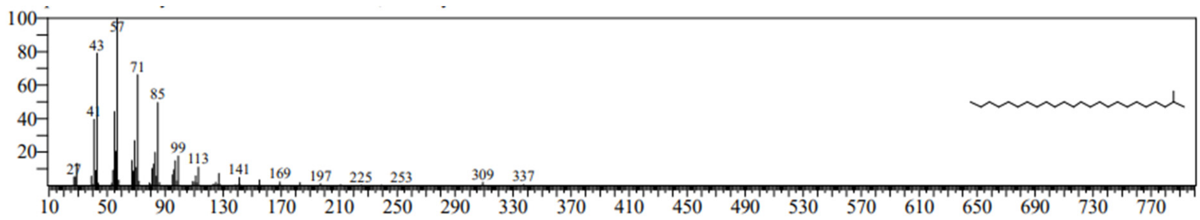

18. Compound name: Octadecane

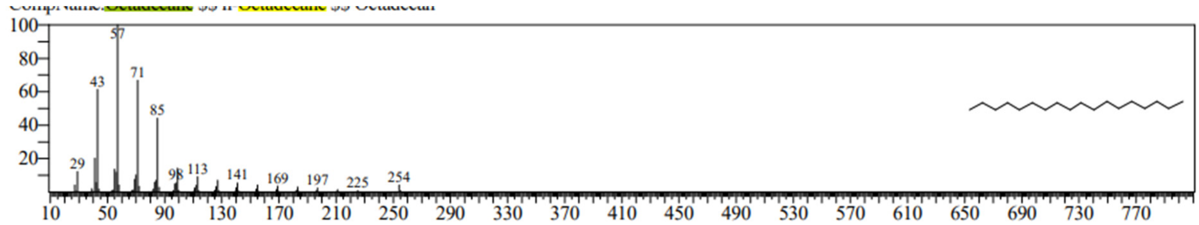

19. Compound name: 3-Eicosene, (E)-

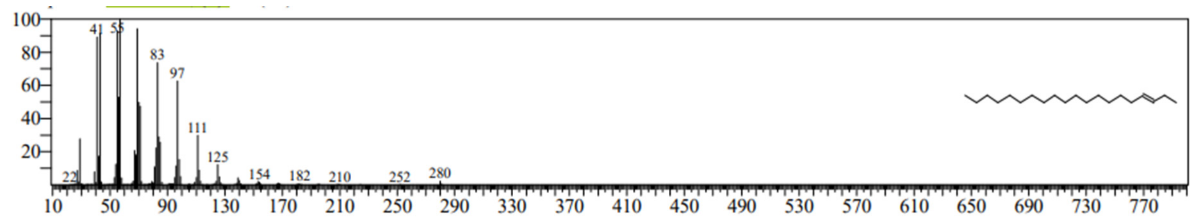

20. Compound name: Tetradecyl trifluoroacetate

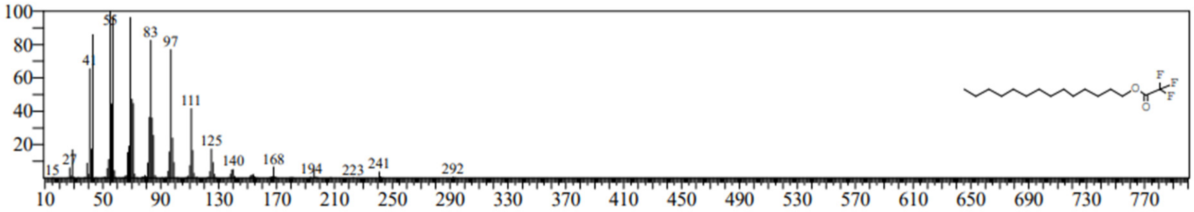

21. Compound name: Eicosane, 1-iodo

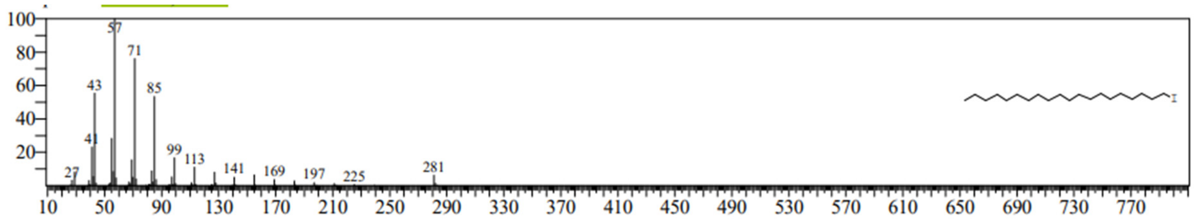

22. Compound name: Docosane

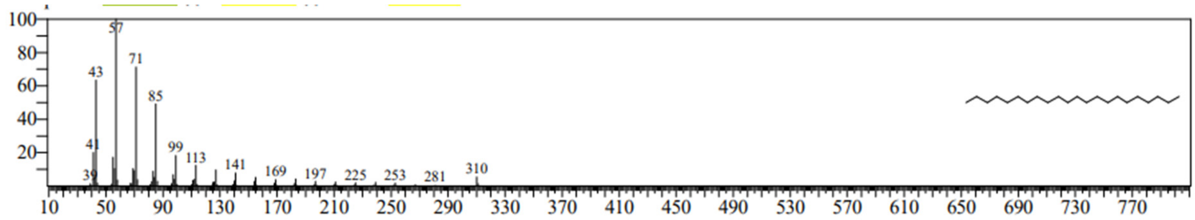

23. Compound name: Hexadecane, 2,6,10,14-tetramethyl

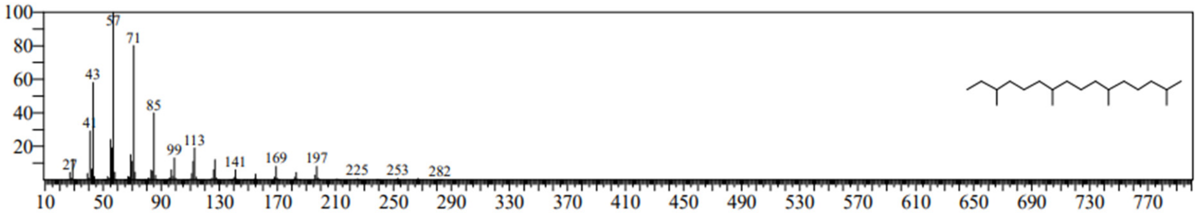

24. Compound name: 5,5-Diethylheptadecane

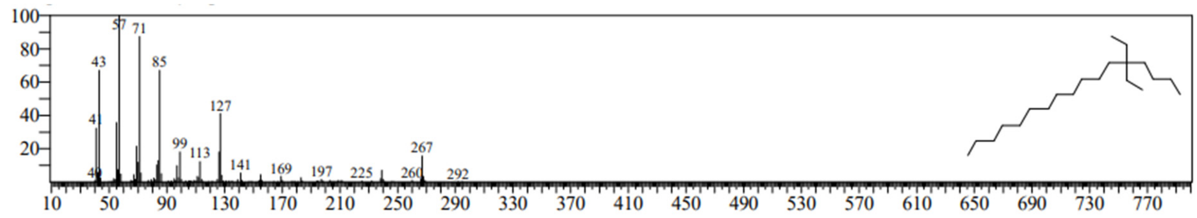

25. Compound name: Cyclopropanecarboxamide, N-(2-butyl)-N-propyl

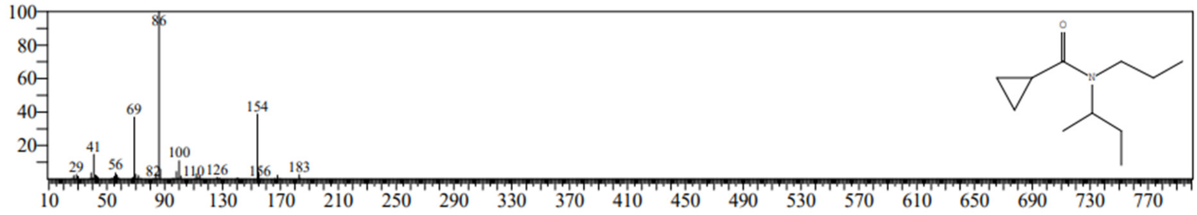

26. Compound name: Fumaric acid, 2-(diethylamino)ethyl tetradecyl ester

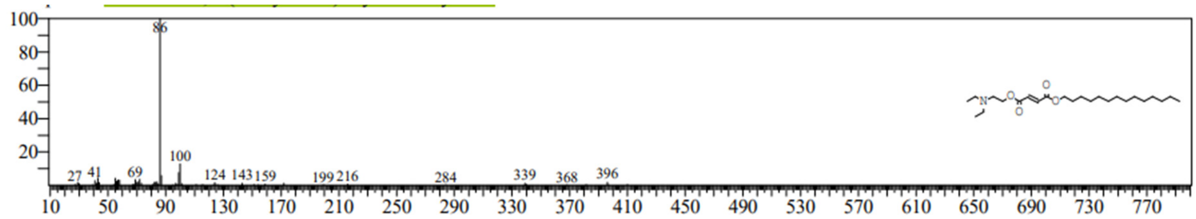

27. Compound name: 3,6-Diisopropylpiperazin-2,5-dione

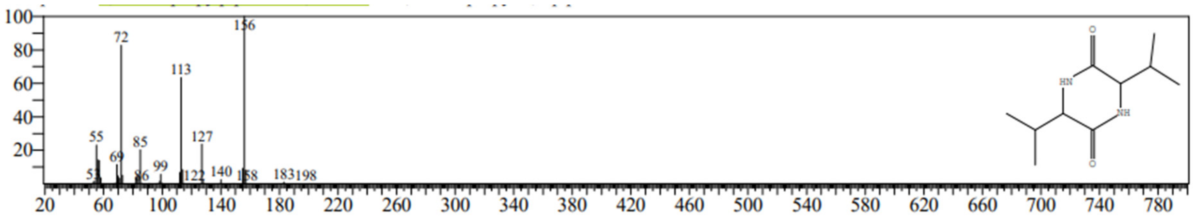

28. Compound name: Butylamine, N,N-dipentyl

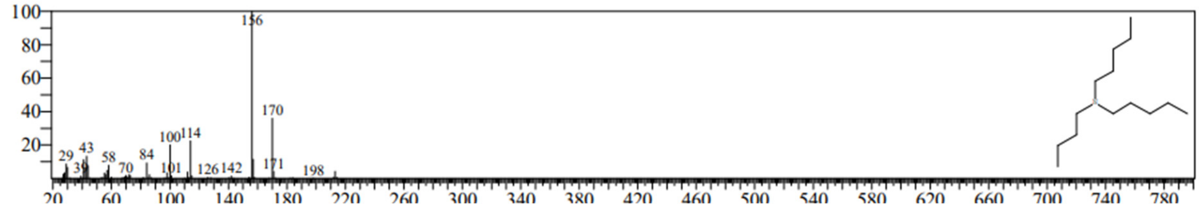

29. Compound name: 7-Ethyl-4,6-heptadecandione

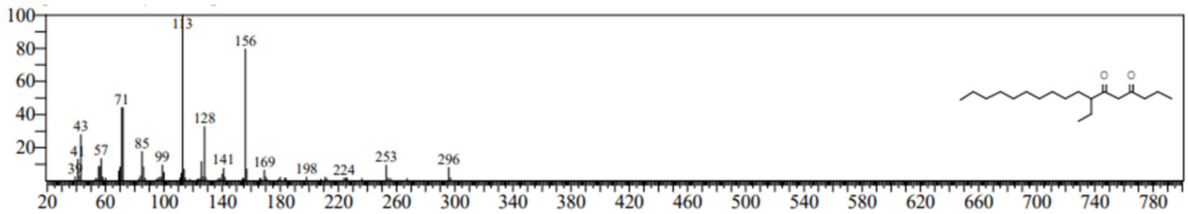

30. Compound name: 2-Diethylaminomethyl-3-hydroxy-6-hydroxymethyl-pyran-4-one

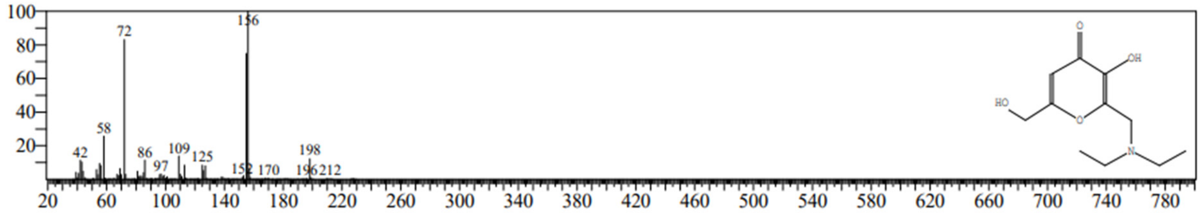

31. Compound name: Butylated Hydroxytoluene

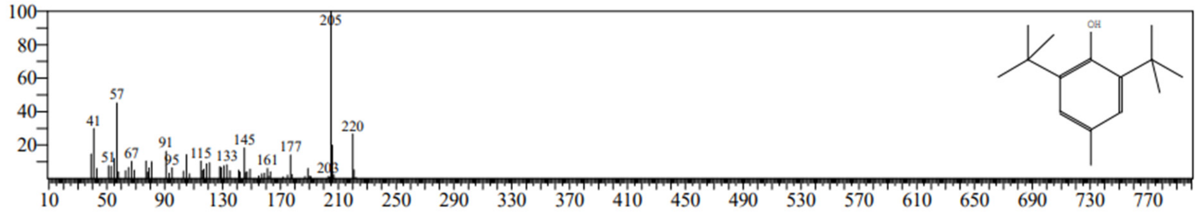

32. Common name: 6,6 Diethylhoctadecane

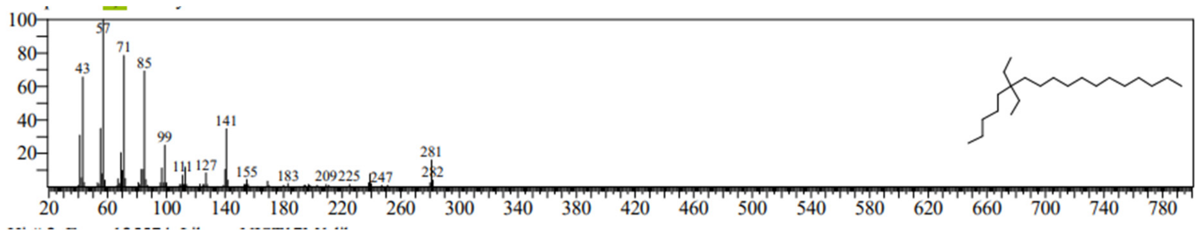

33. Compound name: l-Leucine

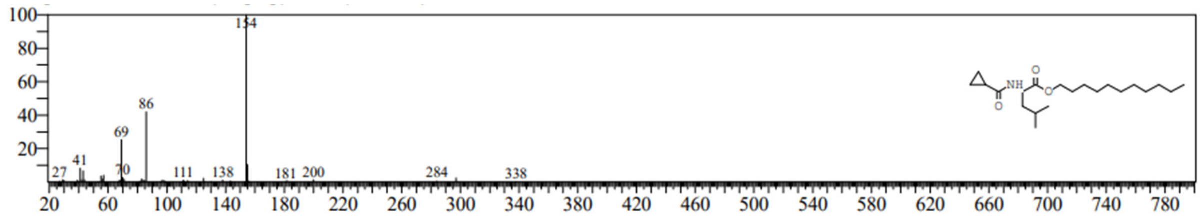

34. Compound name: l-(+)-Ascorbic acid 2,6-dihexadecanoate

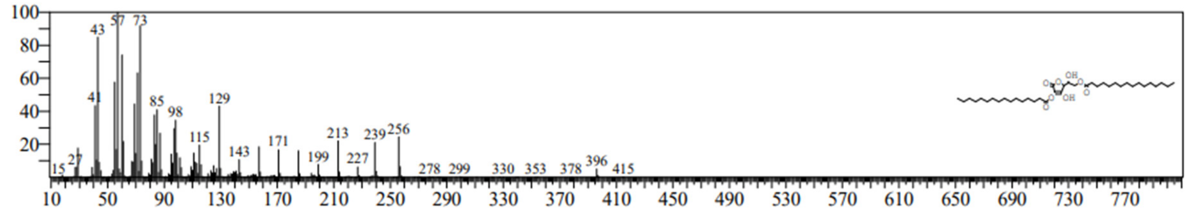

35. Compound name: 1-Heptacosanol

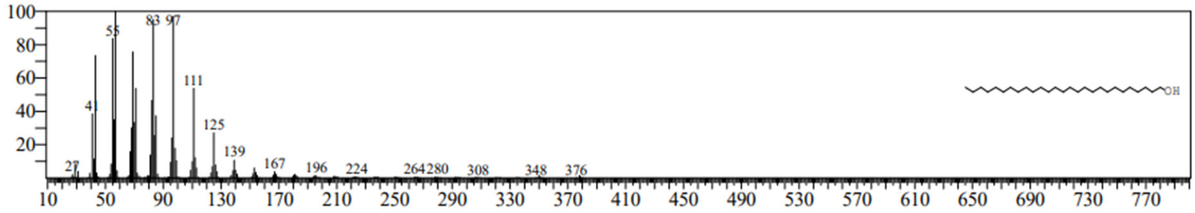

36. Compound name: 1-Heneicosanol

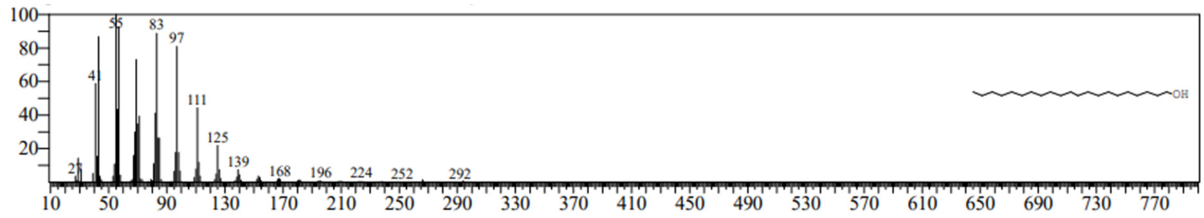

37. Compound name: Pentatriacontane

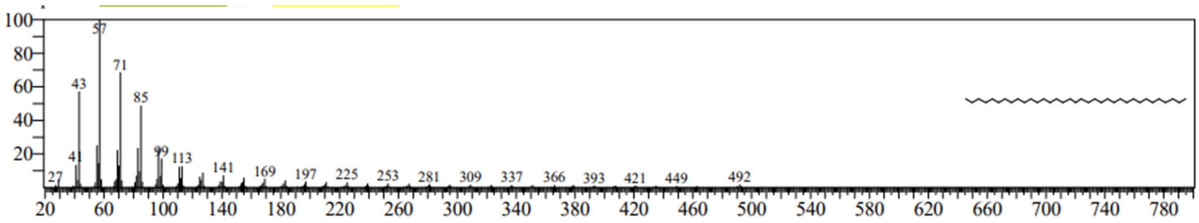

38. Compound name: Hexanamide, N-propyl-N-decyl

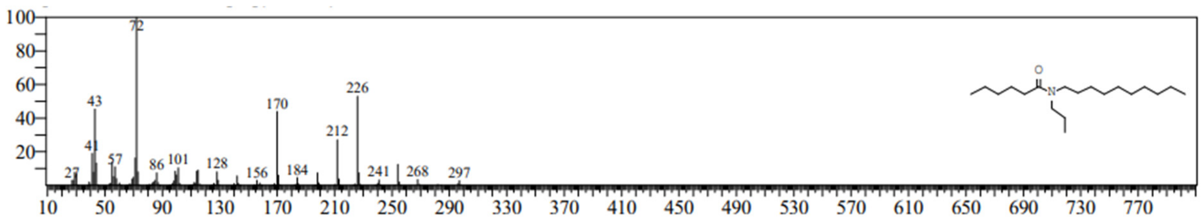

39. Compound name: Acetamide, N-propyl-N-decyl

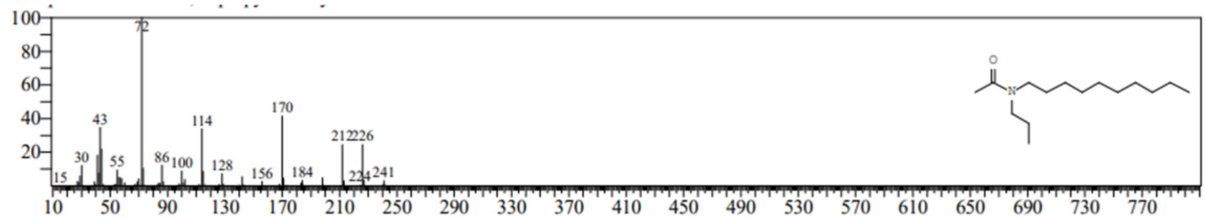

40. Compound name: Octanamide, N-propyl-N-decyl

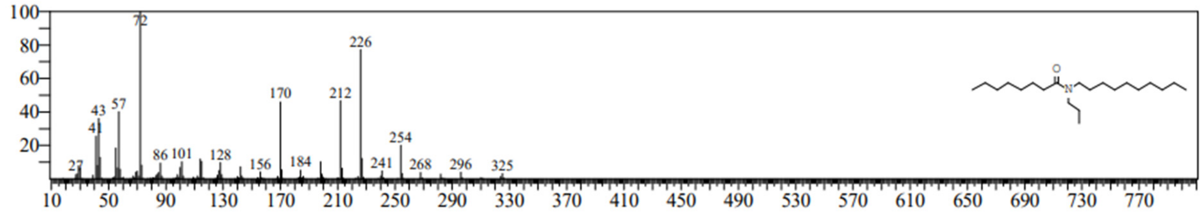

41. Compound name: Benzene, (2,3-dimethyldecyl)-

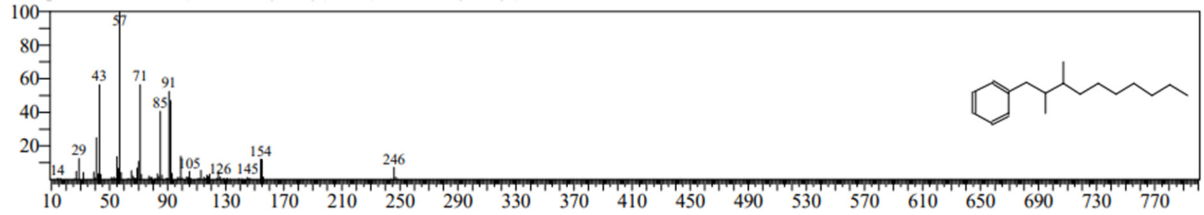

42. Compound name: Acridin-9-yl-[1,2,4]triazol-4-yl-amine

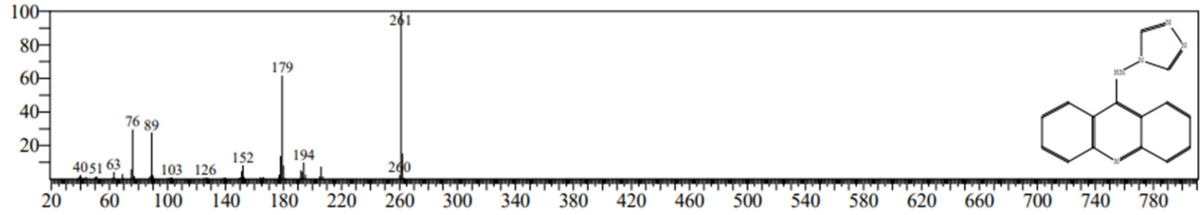

43. Compound name: 1,1'-Biphenyl, 4,4'-bis(bromomethyl)-

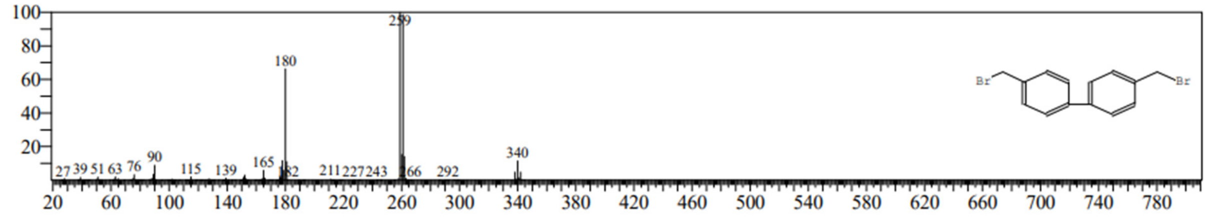

44. Compound name: Silane, trimethyl(3,5-xylyloxy)-

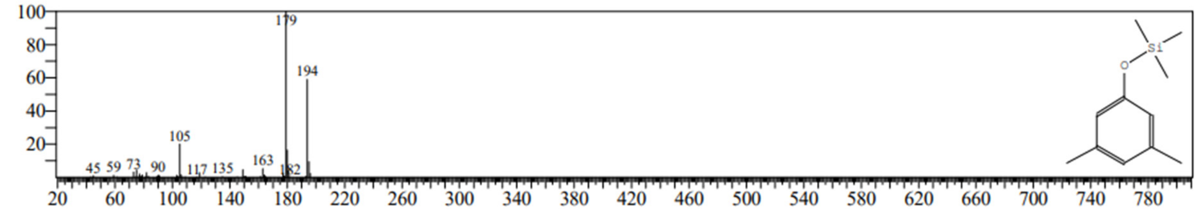

45. Compound name: Eicosyl nonyl ether

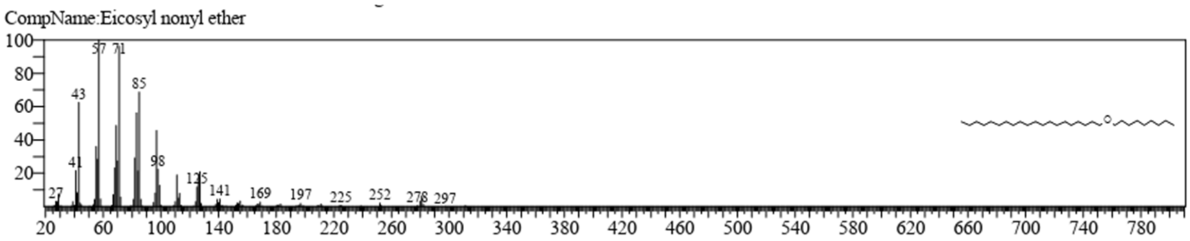

46. Compound name: Ergotaman-3',6',18-trione, 9,10-dihydro-12'-hydroxy-2'-methyl-5'-(phenylmethyl)-, (5'.alpha.,10.alpha.)-

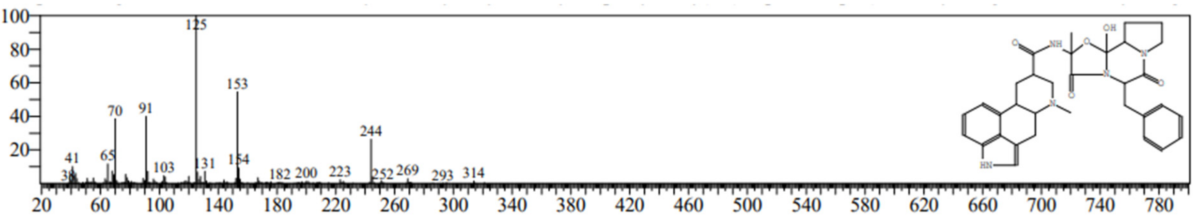

47. Compound name: 1,3-Dipalmitin, TMS derivative

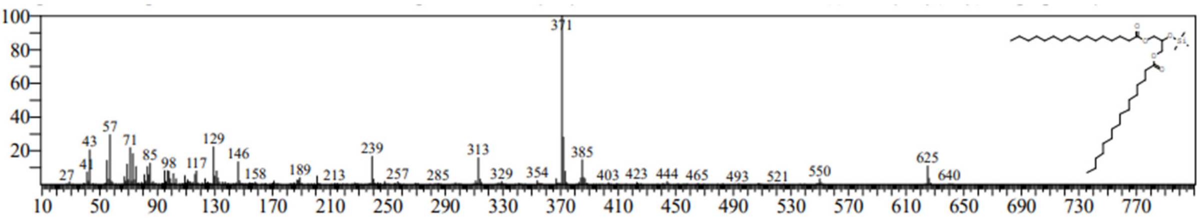

48. Compound name: 11-Methylpentacosane

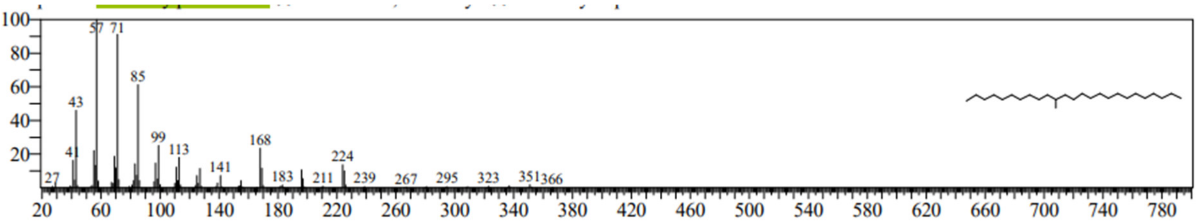

Mass fragmentation patterns and structural elucidations of unique secondary metabolites secreted by *Serendipita indica* and *Zhihengliuella* sp. ISTPL4 in presence of arsenic stress

1. Compound name: d-Ribose, 2-deoxy-bis(thioheptyl)-dithioacetal

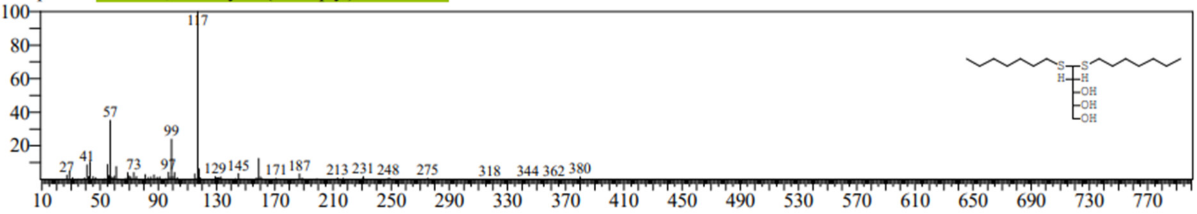

2. Compound name: Phenol, 2,4-bis(1,1-dimethylethyl)-, phosphite

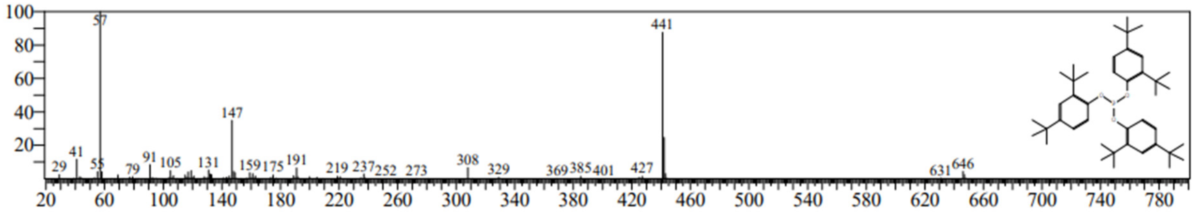

3. Compound name: Heptadecane, 8-methyl

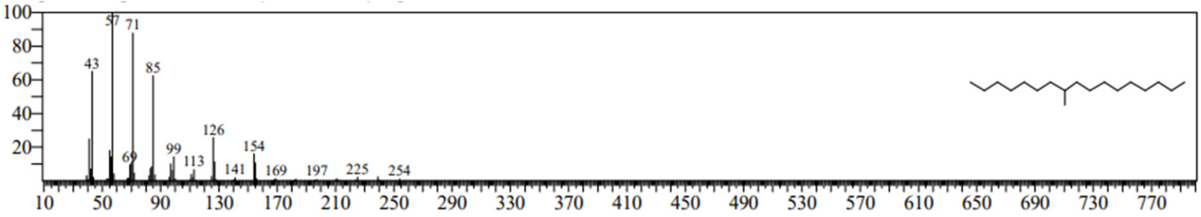

4. Compound name: 2,6,10-Trimethyltridecane

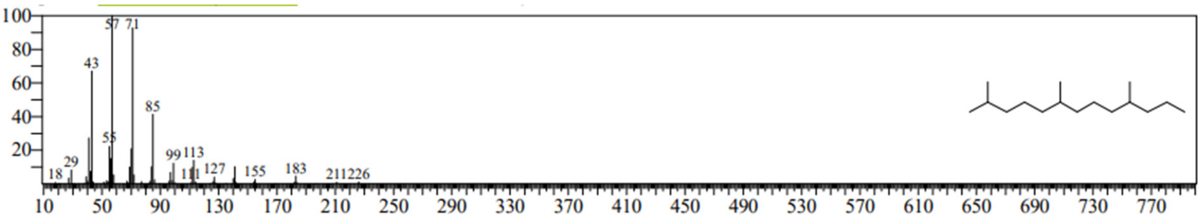

5. Compound name: Phenol, 2,5-bis(1,1-dimethylethyl)

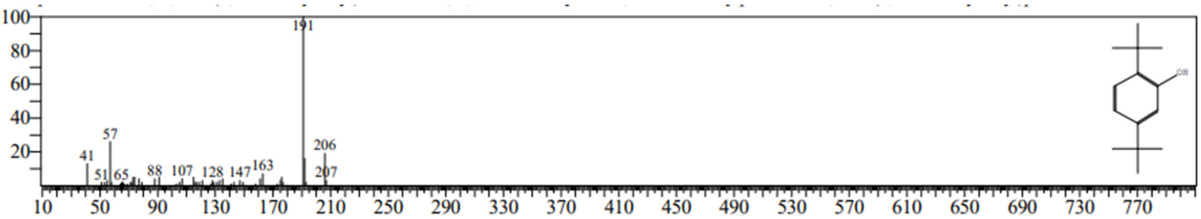

6. Compound name: 5-Azacytosine, N,N,O-trimethyl

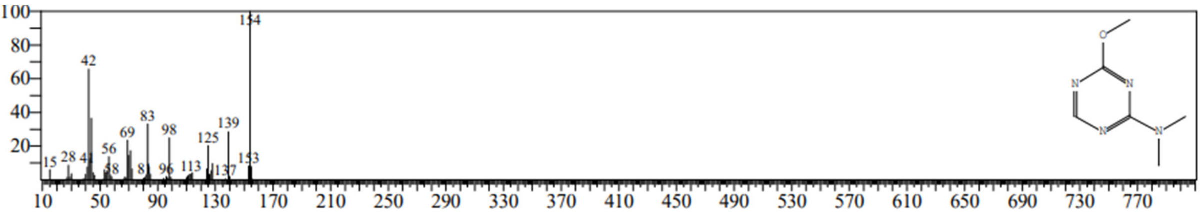

7. Compound name: Butylated Hydroxytoluene

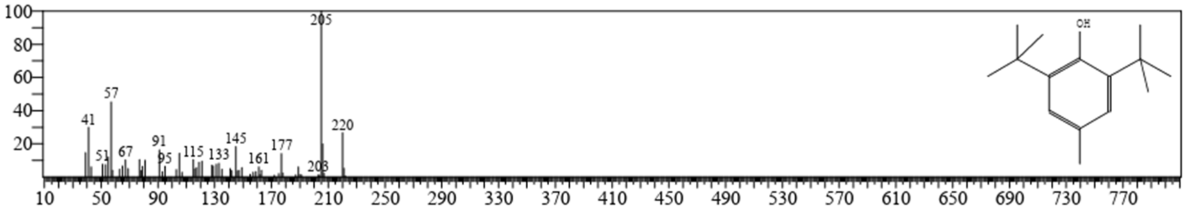

8. Compound name: 5-Nitroso-2,4,6-triaminopyrimidine

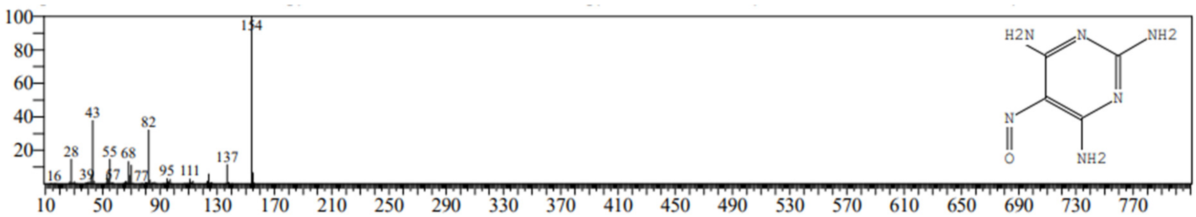

9. Compound name: Heptadecanoic acid, 16-methyl-, methyl ester

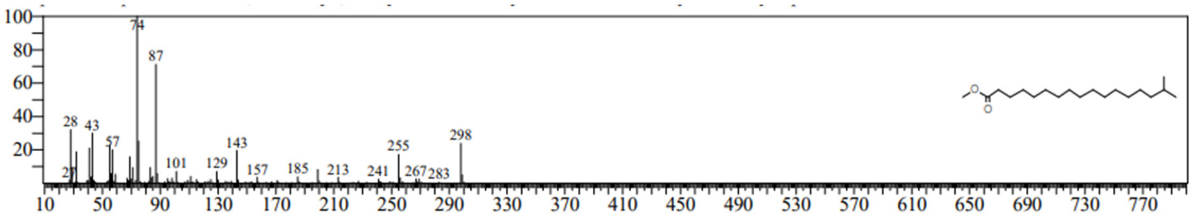

10. Compound name: 1,3,5-Trisilacyclohexane

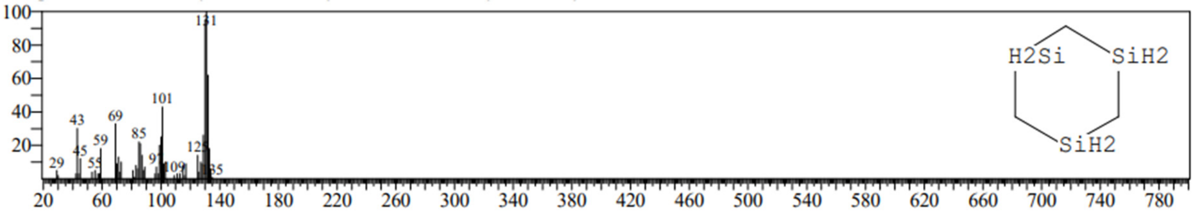

11. Compound name: 1,3-Dipalmitin, TMS derivative

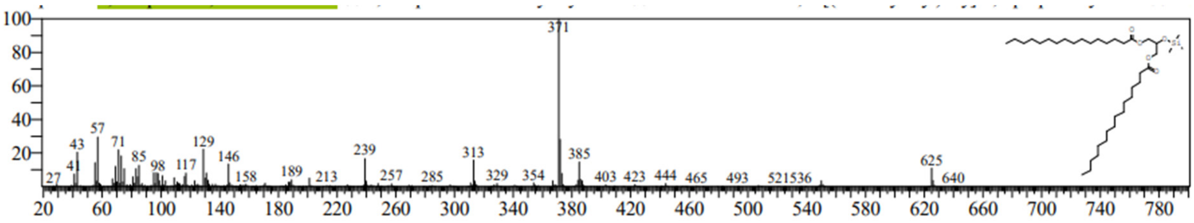

12. Compound name: Tridecanoic acid, 2-ethyl-2-methyl-, ethyl ester

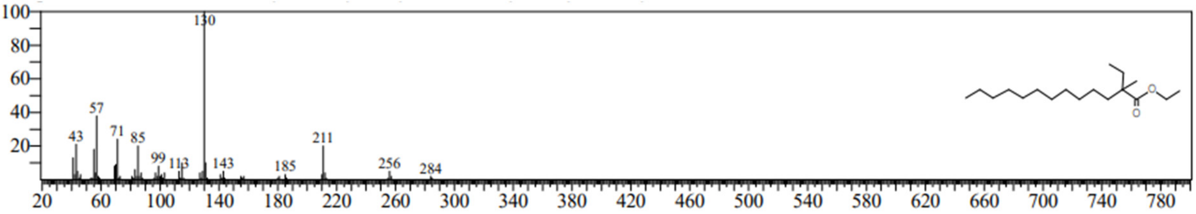

13. Compound name: Formamide, N-(4-[2-(1,1-dimethylethyl)-5-oxo-1,3-dioxolan-4-yl]butyl)

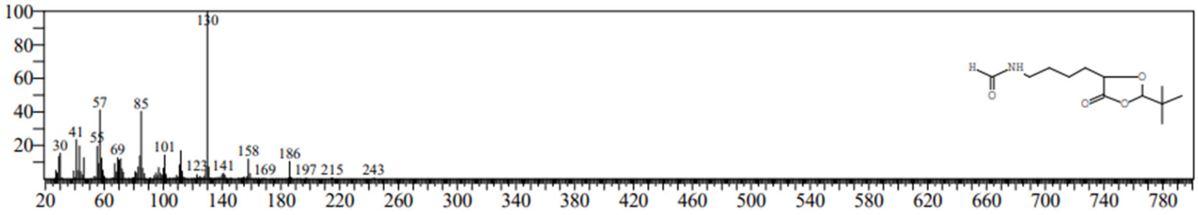

14. Compound name: d-Ribose, 2-deoxy-bis(thioheptyl)-dithioacetal

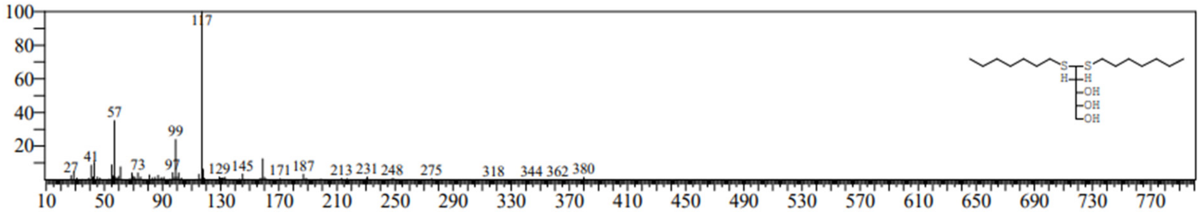

15. Compound name: Hexadecanoic acid, 2-hydroxy-1-(hydroxymethyl)ethyl ester

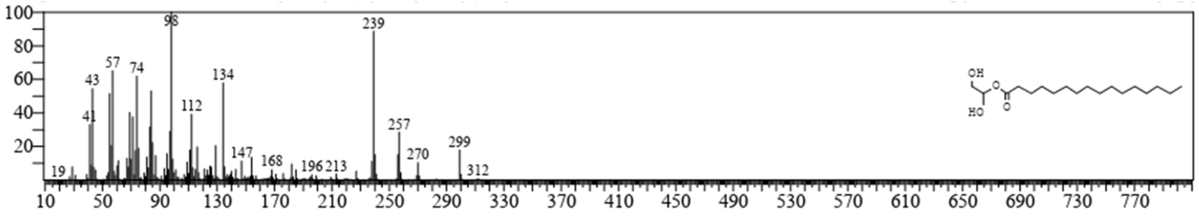

16. Compound name: 1H-Indene, 1-hexadecyl-2,3-dihydro

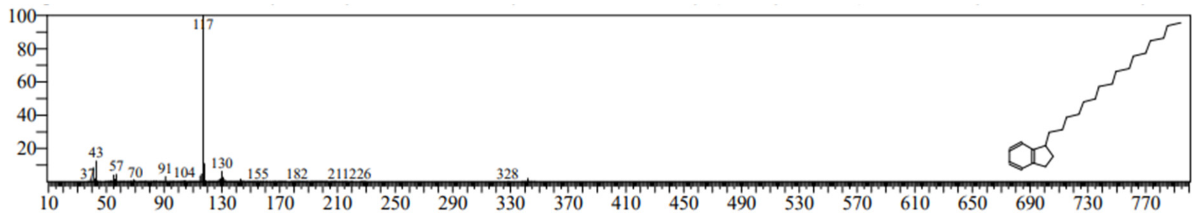

17. Compound name: Ethyl 3-hydroxytetracosanoate

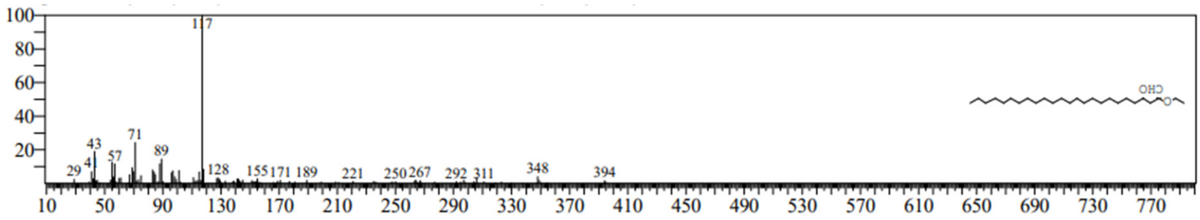

18. Compound name: Palmitic Acid, TMS derivative

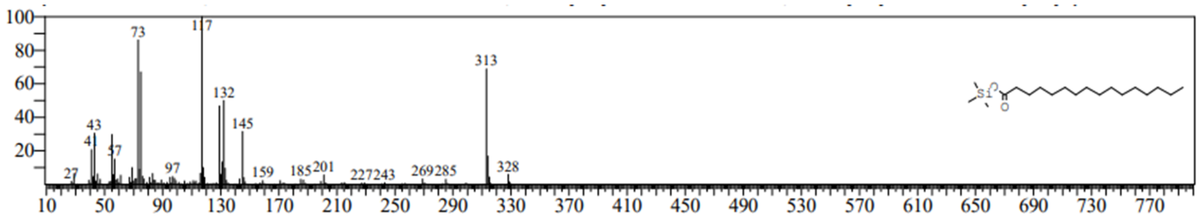

19. Compound name: Silane, diethylheptyloxyoctadecyloxy

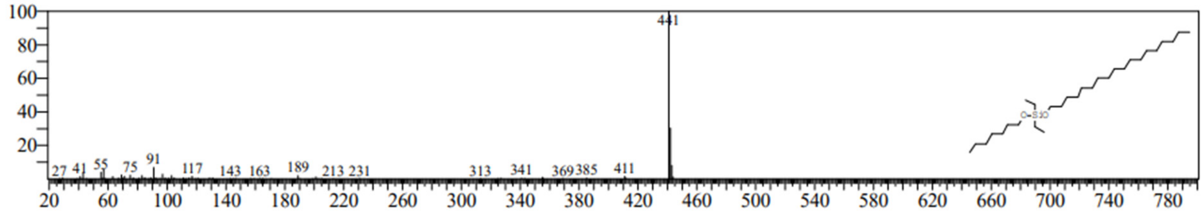

20. Compound name: Silane, dimethyl(docosyloxy)butoxy

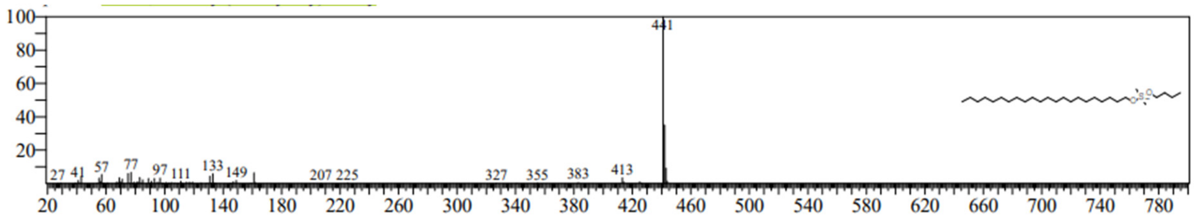

21. Compound name: Quercetin, 5TMS derivative

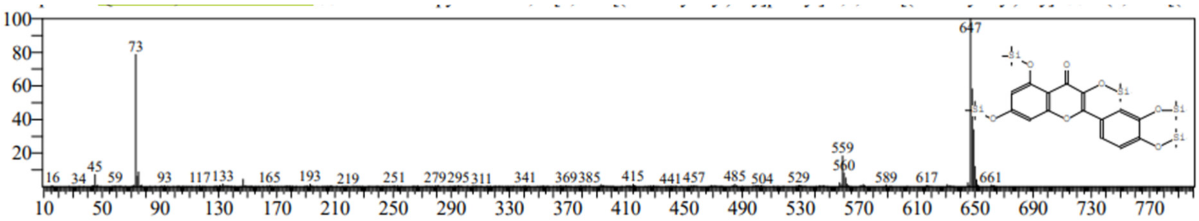

22. Compound name: Pentacyclo[19.3.1.1(3,7).1(9,13).1(15,19)]octacosa-1(25),3,5,7(28),9,11,13(27),15,17,19(26),21,23-dodecaene-25,26,27,28-tetrol, 5,11,17,23-te

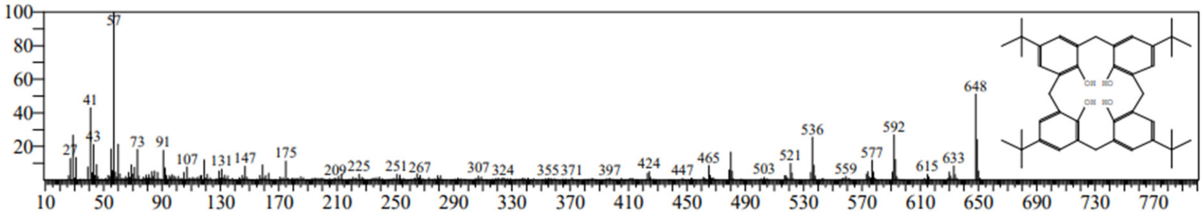

Table S1. Secondary metabolites production in *Serendipita indica* under normal conditions

S. No	Metabolites	Area %	Retention time	Molecular formula	Molecular weight	Functions
1	Olean-18-ene		-	$C_{30}H_{50}$	410	Antiviral activity [22]
2	l-Leucine			$C_{21}H_{39}NO_3$	353	L-Leucine can be assimilated by bacteria when sugars or other preferential carbon sources in the habitat are depleted [23]
3	L-Proline		-	$C_{27}H_{51}NO_3$	437	Antioxidant activity and protect plants under stress conditions [24]
**4**	l-(+)-Ascorbic acid 2,6-dihexadecanoate		-	$C_{38}H_{68}O_8$	652	Antimicrobial activity [25]
5	Heptadecyl trifluoroacetate	-	-	$C_{19}H_{35}F_3O_2$	352	Antioxidant activity [26]
6	5,5-Diethylpentadecane	-	-	$C_{19}H_{40}$	268	Antimicrobial activity [27]
7	Cyclo(L-prolyl-L-valine)	1.82	16.783	$C_{10}H_{16}N_2O_2$	196	It is a metabolite involved in the signaling process by releasing AHL. [28]
8	Heneicosane	0.13	14.893	$C_{21}H_{44}$	296	Antimicrobial activity against *Streptococcus*

						pneumoniae and *Aspergillus fumigatus* [29,44]
9	Ergotaman-3',6',18-trione			$C_{33}H_{37}N_5O_5$	583	Antimicrobial activity [30]
10	Glycine	-	-	$C_{10}H_{19}NO_4$	217	It is a proteinogenic amino acids which help in abiotic stress in plant [31]
11	Quinoline-4-carboxamide 2-phenyl-N-n.-octyl	-	-	$C_{24}H_{28}N_2O$	360	Antibacterial activity [32]
12	Phenol, 2,4-bis (1,1-dimethyl ethyl)-, phosphite	13.23	32.943	$C_{42}H_{63}O_3P$	646	Antioxidant and antifungal activity [33]
13	Lycopene	-	-	$C_{40}H_{66}$	546	Anticancer agent [38]
14	Methanone, (3,5-dimethyl-1-piperidyl)(2-phenyl-4-quinolinyl)-	-	-	$C_{23}H_{24}N_2O$	344	Antioxidant activity [79]<br>It generated NO and ROS during stress in plant [33,79]
15	Formamide, N-(4-[2-(1,1-dimethylethyl)-5-oxo-1,3-dioxolan-4-yl]butyl)			$C_{12}H_{21}NO_4$	243	Antimicrobial activity [41]
16	2-(4-Hydroxy-4-methyl-tetrahydro-pyran-3-ylamino)-3-(1H-indol-2-yl)-propionic acid	-	-	$C_{17}H_{22}N_2O_4$	318	Probiotic compound [42]
**17**	Nonadecane	0.38	14.028	$C_{19}H_{38}$	266	Antibacterial activity [44]
**18**	Eicosane	0.18	12.511	$C_{20}H_{42}$	282	Antifungal, antibacterial, larvicidal activity [45]
19	Dodecane,4,6-dimethyl	0.23	10.212	$C_{14}H_{30}$	198	Antimicrobial activity [46]
20	Dodecane, 2,6,11-trimethyl			$C_{15}H_{32}$	212	Antimicrobial activity [46]
21	Tetradecane	0.33	11.371	$C_{14}H_{28}$	196	Nematicidal activity [47]
22	2-Methylhexacosane	-	-	$C_{27}H_{56}$	380	antimicrobial activity [47]
23	2-Propenoic acid, pentadecyl ester	0.98	15.173	$C_{18}H_{34}O_2$	282	Not found [48]
**24**	1-Hexadecanol	0.62	13.938	$C_{16}H_{34}O$	242	Antifungal and

						antifeedant ability [48]
25	1,3-Benzenedicarboxylic acid, bis(2-ethylhexyl) ester	0.54	25.280	$C_{24}H_{38}O_4$	390	Antimicrobial activity [48]
**26**	2-Propenoic acid, pentadecyl ester	0.98	15.173	$C_{18}H_{34}O_2$	282	Not found [48]
**27**	Octadecane	0.27	16.396	$C_{18}H_{38}$	254	Antifungal activity [48,49]
**28**	Isopropyl myristate	0.27	16.656	$C_{17}H_{34}O_2$	270	Antifungal and antioxidant activity [50]
**29**	Trifluoro acetoxy hexadecane	1.00	17.328	$C_{18}H_{33}F_3O_2$	338	Antifungal activity [51]
**30**	Tetracosane	1.07	17.600	$C_{24}H_50$	338	Antimicrobial activity [51]
**31**	7,9-Di-tert-butyl-1-oxaspiro (4,5) deca-a-6,9-diene-2,8-dione	2.01	17.634	$C_{17}H_{24}O_3$	276	Antimicrobial and antioxidant activity [53]
**32**	Hexadecenoic acid, methyl ester	0.71	17.765	$C_{17}H_{34}O_2$	270	Antifungal activity [54]
**33**	Pyrrole[1,2-a] pyrazine-1,4-dione, hexahyd dro-3-(2-methyl propyl)	1.30	17.829	$C_{11}H_{18}N_2O_2$	210	Antimicrobial activity [55]
**34**	Hexacosyl nonyl ether	0.71	17.920	$C_{35}H_{72}O$	508	Antimicrobial activity [56]
**35**	Dibutyl phthalate	1.49	18.134	$C_{16}H_{22}O_4$	278	Antioxidant and antimicrobial activities [56]
**36**	l-(+)-Ascorbic acid 2,6-dihexadecanoate	1.03	18.255	$C_{38}H_{68}O_8$	652	Antiallergic, Antibacterial, Termiticide and Antiviral activity [57,58]
37	Phthalic acid, 5-methylhex-yl butyl ester	0.44	18.311	$C_{19}H_{28}O_4$	320	Contaminant- [59]
**38**	Isopropyl palmitate	0.60	18.765	$C_{19}H_{38}O_2$	298	It has antiproliferative activity [60]
**39**	1,2-Benzenedicarboxylic acid, butyl octyl ester	0.28	19.114	$C_{20}H_{30}O_4$	334	Antioxidant activity [61]
**40**	n-Tetracosanol-1	0.81	19.426	$C_{24}H_{50}O$	354	Antioxidant activity [62]
**41**	2-Methyltetracosane	0.14	19.500	$C_{25}H_{52}$	352	Free-radical scavenging activity [63]
**42**	Nonadecane nitrile	0.16	19.637	$C_{19}H_{37}N$	279	Antifungal activity [64]
43	Methyl stearate	0.76	19.804	$C_{19}H_{38}O_2$	298	Antifungal, free-radical

						scavenging activity [65]
44	Dodecane, 1,1-dimethoxy- Lauraldehyde	0.63	20.244	$C_{14}H_{30}O_2$	230	Antifungal, antimicrobial antibacterial activities [66]
45	Octadecanoic acid 3-oxo-, ethyl ester	0.19	22.945	$C_{20}H_{38}O_3$	326	Antifungal activity [67]
46	2-Ethylbutyric acid, eicosyl ester	2.16	23.131	$C_{26}H_{52}O_2$	396	Antioxidant, anti-inflammatory, Antioxidant activity, anticancer [68]
**47**	Hexadecanoic acid, 2-hydroxy-1- (hydro oxymethyl) ethyl ester	22.44	23.344	$C_{19}H_{38}O_4$	330	Antioxidant, nematicidal activity [69]
48	Octadecanoic acid, 2,3-dihydroxypropyl ester	7.31	25.087	$C_{21}H_{42}O_4$	358	Antimicrobial, nematocidal [69]
**49**	Bis(2-ethylhexyl) phthalate	2.11	23.457	$C_{24}H_{38}O_4$	390	Plasticizers [70]
**50**	Octocrylene 2-Propenoic acid,	0.16	24.490	$C_{24}H_{27}NO_2$	361	Antioxidant activities [70,71]
51	Squalene e 2,6,10,14,18,22-Tetracosahexaene,	2.19	25.953	$C_{30}H_50$	410	It is a precursor of various hormones and having antioxidant activities [72]
52	Tris (2,4-di-tert-butyl phenyl) phosphate	0.98	33.436	$C_{42}H_{63}O_4P$	662	Antioxidant activity [73]
53	2-methyl tetracosane	0.14	19.515	$C_{25}H_{52}$	352	Antifungal activity [74,75]
54	2,6,10-Trimethyltridecane			$C_{16}H_{34}$	226	Antimicrobial activity [76]
55	2-Isopropyl-5-methyl-1-heptanol			$C_{11}H_{24}O$	172	Antimicrobial activity [78]
56	Behenic alcohol	-	-	$C_{22}H_{46}O$	326	Fungicidal, larvicidal activity [79]
57	N-2-ethylhexyl acetone imine	-	-	$C_{11}H_{23}N$	169	Antimicrobial activity [80]
58	N(1)-(3-Methyl-1,2,4-oxadiazol-5-yl)-1-	-	-	$C_8H_{13}N_5O$	195	Antifungal activity [81]

	pyrrolidinecarboxamidine					
59	Propionitrile 3-(3,5-di-tert-butyl-4-hydroxyphenyl)thio-	-	-	$C_{17}H_{25}NOS$	291	Antimicrobial activity [81]
60	Octahydro-2H-pyrido(1,2-a)pyrimidin-2-one	-	-	$C_8H_{14}N_2O$	154	Antimicrobial activity [82]
61	2,4-Di-tert-butyl-phenol) phosphate	2.37	12.909	$C_{14}H_{22}O$	206	It has fungicidal activity against (Aspergillus niger, Fusarium oxysporum and Penicillium chrysogenum [86,87]
62	Heptacos-1-ene		-	$C_{27}H54$	378	Antimicrobial activity [88]
63	12,12-Dimethoxydodecanoic acid		-	$C_{15}H_{30}O_4$	274	Antimicrobial activity [89]
64	Nonyl tetradecyl ether	0.18	15.571	$C_{23}H_{48}O$	340	Antimicrobial activity [90]
65	1,2-Benzenedicarboxylic acid, bis (2-methyl propyl) ester	3.67	17.108	$C_{16}H_{22}O_4$	278	Antimicrobial activity [90]
66	Pentadecanoic acid, methyl ester	0.36	16.350	$C_{18}H_{34}O_2$	282	Antimicrobial activity [90]
67	Dodecane, 2,6,11-trimethyl			$C_{15}H_{32}$	212	Antimicrobial activity [91]
68	Docosanoic acid, ethyl ester	0.52	18.475	$C_{24}H_{48}O_2$	368	Antimicrobial activity [91]
69	Silane	-	-	$C_{29}H_{62}O_2Si$	470	Antimicrobial activity [93]

Table S2. Secondary metabolites production in *Serendipita indica* in the presence of arsenic stress

**S. No**	**Metabolites**	**Area(%)**	**Retention time**	**Molecular formula**	**Molecular weight**	**Function**
**1**	Olean-18-ene	-	-	$C_{30}H_{50}$	410	Antiviral activity [22]
**2**	l-Leucine	-	-	$C_{21}H_{39}NO_3$	353	L-Leucine can be assimilated by bacteria when sugars or other preferential carbon sources in the habitat

						are depleted [23]
**3**	L-Proline		-	$C_{27}H_{51}NO_3$	437	Antioxidant activity [24]
4	Cyclo(L-prolyl-L-valine)	2.01	16.788	$C_{10}H_{16}N_2O_2$	196	It is a metabolite involved in the signaling process by releasing AHL [28]
5	Ergotaman-3',6',18-trione,	-	-	$C_{33}H_{37}N_5O_5$	583	Antimicrobial activity [30]
6	Phenol, 2,4-bis (1,1-dimethyl ethyl)-, phosphite	0.83	32.914	$C_{42}H_{63}O_3P$	646	Antioxidant activity [33]
7	5,9,13,17-Tetramethyl 4,8,12,16-octadecatetraenoic acid	-	-	$C_{22}H_{36}O_2$	332	Antidiabetic and antiaging property [35]
8	Tetramethyl octadecatetraenoic acid	-	-	$C_{22}H_{36}O_2$	331	Antidiabetic and antiaging property [35]
9	Glycerol tri caprylate	0.26	27.393	$C_{27}H_{50}O_6$	470	Antifungal activity [36]
10	2-(4-Hydroxy-4-methyl-tetrahydro-pyran-3-ylamino)-3-(1H-indol-2-yl)-propionic acid	-	-	$C_{17}H_{22}N_2O_4$	318	Probiotic compound [42]
11	3-Indol-1-yl-propionic acid, methyl ester	-	-	$C_{17}H_{22}N_2O_4$	318	Probiotic compound [42]
12	Tridecanoic acid, 12-methyl-, methyl ester	0.38	16.370	$C_{15}H_{30}O_2$	242	Antimicrobial, insecticidal activities [43]
13	7,9-Di-tert-butyl-1-oxaspiro (4,5) Deca-6,9 9-diene-2,8-dione	2.04	17.633	$C_{17}H_{24}O_3$	276	Antimicrobial and antioxidant activites [53]
14	Hexadecanoic acid, methyl ester	1.33	17.772	$C_{17}H_{34}O_2$	270	Antifungal activity [53]
15	Hexadecanoic acid, 2-hydroxy-1- (hydro oxymethyl) ethyl ester Palmitin,	44.99	23.334	$C_{19}H_{38}O_4$	330	Antimicrobial and antioxidant activities [67]
16	2-Ethylbutyric acid, eicosyl ester	3.50	23.139	$C_{26}H_{52}O_2$	396	Antimicrobial, anti-

						inflammatory, activities [68]
17	Octadecanoic acid, 2,3-dihydroxypropyl ester Stearin	14.24	25.080	$C_{21}H_{42}O_4$	358	Antimicrobial, nematicidal activity [69]
18	Octocrylene 2-Propenoic acid,	0.59	24.490	$C_{24}H_{27}NO_2$	361	Antifungal activity [71]
19	Tris (2,4-di-tert-butyl phenyl) phosphate	2.07	35.431	$C_{42}H_{63}O_4P$	662	antioxidant activity [73]
20	n-Hexadecanoic acid	1.44	18.239	$C_{16}H_{32}O_2$	256	Nematicidal, antioxidant [81]
21	Octahydro-2H-pyrido(1,2-a)pyrimidin-2-one	-	-	$C_8H_{14}N_2O$	154	Antimicrobial activity [82,83]
22	2,4-Di-tert-butyl-phenol	9.52	12.910	$C_{14}H_{22}O$	206	Antimicrobial activity [84–86]
23	1,2-Benzenedicarboxylic acid, bis (2-methyl propyl) ester	0.67	17.125	$C_{16}H_{22}O_4$	278	Antimicrobial activity [90,92]
24	Pyrrole[1,2-a] pyrazine-1,4-dione, hexahydro-3-(2-methyl propyl)	1.30	17.829	$C_{11}H_{18}N_2O_2$	210	Antimicrobial activity [94]
25	Methyl stearate	1.69	19.803	$C_{19}H_{38}O_2$	298	Antifungal activity [95]
26	Tetratriacontyl heptafluorobutyrate	0.32	20.729	$C_{38}H_{69}F_7O_2$	690	Antimicrobial activity [96]
27	Myristic acid, glycidyl ester Tetradecanoic acid	0.59	21.404	$C_{17}H_{32}O_3$	284	Antifungal properties, Antioxidant, nematicidal activities [97]
28	2-Propenoic acid, 3-(4-methoxyphenyl)	0.70	21.702	$C_{18}H_{26}O_3$	290	Antimicrobial activity [98]
29	1,2,3,4-Tetrahydro-3- (phenyl acetamido) quinoline	0.11	22.935	$C_{17}H_{18}N_2O$	266	Antimicrobial activity [99]
30	13-Docosenamide, (Z)- Erucylamide	1.79	25.696	$C_{22}H_{43}NO$	337	Antimicrobial, antioxidant, anti-

						inflammatory [100,101]
31	Squalene e 2,6,10,14,18,22-Tetracosahexaene,	0.80	25.946	$C_{30}H_{5}0$	410	Antibacterial, antioxidant [102]
32	2-(Decanoyloxy) propane-1,3-diyl dioctanoate	0.93	30.051	$C_{29}H_{54}O_{6}$	498	Antioxidant activity [103]
33	Heptadecanoic acid, 16-methyl-, methyl ester	-	-	$C_{19}H_{38}O_{2}$	298	Used in anti-skin cancer drug [104]
34	Tetrapentacontane, 1,54-dibromo-	-	-	$C_{54}H_{108}Br_{2}$	914	Antifungal activity [105]
35	Triacontyl heptafluorobutyrate	-	-	$C_{34}H_{61}F_{7}O$ 2	634	Antimicrobial activity [106]
36	Hexatriacontyl trifluoroacetate	-	-	$C_{38}H_{73}F_{3}O_{2}$	618	Antifungal activity [107]
37	2-Ethylhexyl trans-4-methoxycinnamate	-	-	$C_{18}H_{26}O_{3}$	290	Antimicrobial activity [108]
38	9-Octadecenamide	-	-	$C_{18}H_{35}NO$	281	It is a compound that is used in the medical industry for mood and sleep disorder [109]
39	1,2,3,4-Tetrahydronaphthalen-1-yl 2,2,3,3,3-pentafluoropropanoate	-	-	$C_{13}H_{11}F_{5}O_{2}$	294	Antimicrobial activity [110]
40	L-Tryptophan,N-methyl-, methyl ester	-	-	$C_{13}H_{16}N_{2}O_{2}$	232	Antimicrobial activity [111]
41	2-Ethylbutyric acid, eicosyl ester	-	-	$C_{26}H_{52}O_{2}$	396	Antimicrobial activity [112]
42	Fumaric acid, 2,2,2-trichloroethyl tridecyl ester			$C_{19}H_{31}C_{13}O_{4}$	428	These are esters used for the treatment of

						psoriasis and sclerosis [113]
43	Cyclooctadecane	-	-	$C_{20}H_{40}$	280	Antifungal activity [114]
44	2,6,10,14,18-Pentamethyl-2,6,10,14,18-eicosapentaene	-	-	$C_{25}H_{42}$	342	Antimicrobial activity [115]
45	Lycopene	-	-	$C_{40}H_{66}$	546	Anticancer agent [73,116]
46	3-(Octanoyloxy)propane-1,2-diyl bis(decanoate)	-	-	$C_{31}H_{58}O_6$	526	Anti-inflammatory activity [117]
47	Pentacyclo[19.3.1.1(3,7).1(9,13).1(15,19)]octacosa-1(25),3,5,7(28),9,11,13(27),15,17,19(26),21,23-dodecaene-25,26,27,28-tetrol, 5,11,17,23-	-	-	$C_{44}H_{56}O_4$	648	Antimicrobial activity [118]

Table S3. Secondary metabolites production in *Serendipita indica* and Z. sp. ISTPL4 under normal conditions

S. No.	Metabolites	Area %	Retention time	Molecular formula	Molecular weight	Function
1	Butylamine, N,N-dipentyl	-	-	C14H31N	213	Antimicrobial activity[13]
2	Eicosyl nonyl ether	-	-	C29H60O	424	Antimicrobial activity[13]
3	Fumaric acid, 2-(diethylamino)ethyl tetradecyl ester	-	-	C24H45NO4	411	These are alkaloids having antibacterial activity[13]
4	Olean-18-ene	-	-	C30H50	410	Antimicrobial activity[22]
5	l-Leucine	-	-	$C_{21}H_{39}NO_3$	353	L-Leucine can be assimilated by bacteria when sugars or other preferential carbon sources in the habitat are depleted[23]
6	L-Proline	-	-	$C_{28}H_{53}NO_3$	451	In view of the abundance and different functions of L-proline, it is not surprising that bacteria employ various L-proline-specific transport

						systems and enzymes allowing the utilization of external L-proline. These7systems play important roles in the adaptation of single-cell organisms to steadily changing environmental conditions as they occur in soil, water and during interactions with eukaryotic hosts.[24]
**7**	l-(+)-Ascorbic acid 2,6-dihexadecanoate	-	-	C38H68O8	652	Antimicrobial activity[25]
**8**	Heneicosane, 10-methyl-	0.18	14.344	C22H46	310	Antimicrobial activity[[26]
**9**	Cyclo(L-prolyl-L-valine)	1.39	-	$C_{10}H_{16}N_2O_2$	196	Class of diketopiperazines (DKPs) the compound cyclo-(L-prolyl–L-valine) activated N-acyl homoserine lactone (AHL) bioreporters, indicating that Archaea may have the ability to interact with AHL-producing bacteria within mixed communities. [28]
**10**	Heneicosane	0.13	14.893	$C_{21}H_{44}$	296	Antimicrobial activity against *Streptococcus pneumoniae* and *Aspergillus fumigatus*[34]
**11**	Silane, trimethyl(3,5-xylyloxy)-	-	-	C11H18OSi	194	Antimicrobial activity[38][97]
**12**	Ergotaman-3',6',18-trione, 9,10-dihydro-12'-hydroxy-2'-methyl-5'-(phenylmethyl)-, (5'.alpha.,10.alpha.)-	-	-	$C_{33}H_{37}N_5O_5$	583	Antimicrobial, Anti-inflammatory, activity[44]
**13**	Eicosane	0.18	12.511	$C_{20}H_{42}$	282	Antifungal, antibacterial,

						larvicidal activity [45]
**14**	3-Eicosene, (E)-	-	-	C20H40	280	Antifungal, antibacterial, larvicidal activity [45]
**15**	Propionamide, N-propyl-N-decyl	-	-	C16H33NO	255	Antimicrobial activity[[45]
**16**	Octadecane	-	-	C18H38	254	Antifungal activity[48]
**17**	Tetracosane	1.07	17.600	$C_{24}H_{5}0$	338	Antimicrobial activity[[52]
**18**	Ergotaman-	-	-	$C_{33}H_{37}N_{5}O_{5}$	581	Antimicrobial, Anti-inflammatory, activity[44]
**19**	Tetracosane	0.49	20.146	C24H50	338	Antimicrobial activity[[52]
**20**	7,9-Di-tert-butyl-1-oxaspiro (4,5) deca-a-6,9-diene-2,8-dione	2.01	17.634	$C_{17}H_{24}O_{3}$	276	Antimicrobial and antioxidant activity[53]
**21**	Hexadecenoic acid, methyl ester	0.71	17.765	$C_{17}H_{34}O_{2}$	270	Antifungal activity[54]
**22**	n-Hexadecanoic acid methyl ester	2.99	18.257	C17H34O2	270	Antimicrobial activity[54]
**23**	I-Nonadecene	0.22	16.323	$C_{19}H_{38}$	266	Antibacterial activity[][62]
**24**	2-Methyltetracosane	0.28	11.578	C25H52	352	Free-radical scavenging activity [63]
**25**	octadecanoic acid, 3-oxo-, ethyl ester	0.59	22.945	C20H38O3	326	Antibacterial activity][67]
**26**	Hexadecanoic acid, 2-hydroxy-1-(hydroxymethyl)ethyl ester	46.06	23.354	C19H38O4	330	Antimicrobial activity **[69]**
**27**	2-Methylhexacosane	0.18	19.421	C27H56	380	Antimicrobial activity[77]
**28**	Octadecanoic acid, 2,3-dihydroxypropy ester	14.81	25.093	C21H42O4	358	Antimicrobial activity[[77]
**29**	Propionamide, N-propyl-N-decyl	0.54	20.558	C16H33NO	255	Antimicrobial activity[80]
**30**	Pyrrole[1,2-a] pyrazine-1,4-dione, hexahyd dro-3-(2-methyl propyl)	1.30	17.829	$C_{11}H_{18}N_{2}O_{2}$	210	Antimicrobial activity[81]

**31**	Diethylpent-4-enylamine	3.35	16.807	C9H19N	141	Antimicrobial activity [84]
**32**	3,6-Diisopropylpiperazin-2,5-dione	0.13	17.158	C10H18N2O2	198	Antimicrobial[[84]
**33**	2,4-Di-tert-butyl-phenol) phosphate	2.37	12.909	$C_{14}H_{22}O$	206	It has fungicidal activity against (Aspergillus niger, Fusarium oxysporum and Penicillium chrysogenum [86]
**34**	1,2-Benzenedicarboxylic acid, bis (2-methyl propyl) ester	3.67	17.108	$C_{16}H_{22}O_4$	278	Antimicrobial activity[92]
**35**	Methyl stearate	0.68	19.802	C19H38O2	298	Antifungal, free-radical scavenging activity[[96]
**36**	Hexadecane	-		$C_{16}H_{34}$	226	It is a long-chain volatile organic compound secreted by bacteria that confer induced resistance against both *Pectobacterium carotovorum* and *Pseudomonas syringae*. [119]
**37**	Tetradecane	0.33	11.371	$C_{14}H_{28}$	196	Nematicidal activity[120]
**38**	Heptadecane	0.22	12.505	C17H36	240	These are the mVOC that trigger plant growth promotion by activating the signaling cascade in plant[[121][122]
**39**	2-Methyltetracosane	-	-	C25H52	352	Free-radical scavenging activity[121][123]
**40**	Tetrapentacontane	0.11	23.465	C54H110	758	Antibacterial and antioxidant activity[124][127]
**41**	Hexanoic acid, heptadecyl ester	3.11	23.174	C23H46O2	354	Antifungal activity[125-127]
**42**	Tetradecyl trifluoroacetate	-	-	C16H29F3O2	310	Antimicrobial activity[128-130]
**43**	6,6-Diethylhoctadecane	0.12	17.690	C22H46	310	Antimicrobial activity[130]
**44**	Eicosane, 1-iodo	-	-	C20H41I	408	Antimicrobial

						activity[[131]
**45**	Docosane	-	-	C22H46	310	Antimicrobial activity[132]
**46**	Hexadecane, 2,6,10,14-tetramethyl	-	-	C20H42	282	Antimicrobial activity[133]
**47**	5,5-Diethylheptadecane	-	-	C21H44	296	Antimicrobial compound[134]
**48**	11-Methylpentacosane	-	-	C26H54	366	Antimicrobial compound[134]
**49**	Cyclopropanecarboxamide, N-(2-butyl)-N-propyl	-	-	C16H31NO	253	Antimicrobial activity[135]
**50**	3,6-Diisopropylpiperazin-2,5-dione	-	-	C10H18N2O2	198	Antimicrobial activity [136]
**51**	2-Diethylaminomethyl-3-hydroxy-6-hydroxymethyl-pyran-4-one	-	-	C11H17NO4	227	Antimicrobial activity[137][138]
**52**	7-Ethyl-4,6-heptadecandione	-	-	C19H36O2	296	Antimicrobial activity[139]
**53**	6,6 Diethylhoctadecane	-	-	C20H42	282	Antimicrobial activity[[140]
**54**	1-Heptacosanol	-	-	C27H56O	396	Antimicrobial activity[141]
**55**	1-Heneicosanol	-	-	C21H44O	312	Antibacterial activity[142]
**56**	Butylated Hydroxytoluene	-	-	C15H24O	220	Antimicrobial[[142]
**57**	2-Methylhexacosane	-	-	C27H56	380	Pharmaceutically important and anticancer activity [143]
**58**	Pentatriacontane	-	-	C35H72	492	Antifungal activity[144][145]
**59**	Hexanamide, N-propyl-N-decyl	-	-	C19H39NO	297	Antimicrobial activity, anti-inflammatory action[146]
**60**	Acetamide, N-propyl-N-decyl	-	-	C15H31NO	241	It is herbicide which controls weeds and grasses[147]
**61**	Octanamide, N-propyl-N-decyl	-	-	C21H43NO	325	Antifungal activity[148]
**62**	Benzene, (2,3-dimethyldecyl)-	-	-	C18H30	246	Antimicrobial activity[149]
**63**	Acridin-9-yl-[1,2,4]triazol-4-yl-amine	-	-	C15H11N5	261	Anti tuberculosis[[150]
**64**	Silane methyl	-	-	C11H18OSi	195	Antimicrobial activity [93]
**65**	1,1'-Biphenyl, 4,4'-	-	-	C14H12Br2	338	Antimicrobial

	bis(bromomethyl)-					activity[151]
**66**	1,3-Dipalmitin, TMS derivative	-	-	C38H76O5Si	640	Antioxidant activity[152]
**67**	Glycerol 1-palmitate	-	-	C19H38O4	330	Antimicrobial activity[153][154]

Table S4. Secondary metabolites production in *Serendipita indica* and Z. sp. ISTPL4 in the presence of arsenic stress

**S. No.**	**Metabolites**	**Area %**	**Retention time**	**Molecular formula**	**Molecular weight**	**Function**
1	L-Proline	-	-	C27H51NO3	437	Antioxidant activity [22]
**2**	5-Azacytosine, N,N,O-trimethyl	-	-	C6H10N4O	154	-Antimicrobial activity [32]
3	Olean-18-ene	-	-	C30H50	410	Antiviral activity [22]
4	5-Nitroso-2,4,6-triaminopyrimidine			C4H6N6O	154	Antimicrobial activities [32]
**5**	Phenol, 2,4-bis(1,1-dimethylethyl)-, phosphite	4.12	32.990	C42H63O3P	646	Antioxidant and antifungal activity [33]
6	Heneicosane	3.23	35.443	C21H44	296	Antimicrobial activity against *Streptococcus pneumoniae* and *Aspergillus fumigatus* [34]
**7**	Ethyl 3-hydroxytetracosa52noate	-	-	C26H52O3	412	Antioxidant activity [35]
**8**	Formamide, N-(4-[2-(1,1-dimethylethyl)-5-oxo-1,3-dioxolan-4-yl]butyl)	-	-	C12H21NO4	243	Antimicrobial activity [41]
9	d-Ribose, 2-deoxy-bis(thioheptyl)-dithioacetal	-	-	C19H40O3S2	380	Antimicrobial activity [41]
**10**	Quercetin, 5TMS derivative		-	C30H50O7Si5	662	Quercetin, a plant-derived polyphenolic flavonoid, has been linked with health benefits in both humans and animals. Quercetin, a natural plant-derived dietary polyphenol,

						possesses a high safety profile and extensive beneficial properties including potent antioxidant, anti-inflammatory, antiviral. [41]
**11**	Tridecanoic acid, 2-ethyl-2-methyl-, ethyl ester	-	-	C18H36O2	284	Antimicrobial, insecticidal activities [43]
**12**	Pyrrolo[1,2-a]pyrazine-1,4-dione, hexahydro-3-(2-methylpropyl)	-	-	C11H18N2O2	210	Antifungal activity [45]
**13**	Eicosane	0.23	12.573	C20H42	282	Antimicrobial activity [45]
**14**	Palmitic Acid, TMS derivative	-	-	C19H40O2Si	328	Antioxidant and antimicrobial activity [52]
**15**	7,9-Di-tert-butyl-1-oxaspiro(4,5)deca-6,9-diene-2,8-dione	1.39	17.138	C17H24O3	276	Antioxidants [53]
**16**	Hexadecanoic acid, methyl ester	0.80	17.780	C17H34O2	270	Antibacterial compound [54]
**17**	Pyrrolo[1,2-a]pyrazine-1,4-dione, hexa hydro-3-(2-methylpropyl)-	1.66	17.965	C11H18N2O2	210	Antifungal activity [55]
18	Methyl stearate	1.60	36.535	$C_{19}H_{38}O_2$	298	Antifungal, antibacterial, and antioxidant activities [63]
19	octadecanoic acid, 3-oxo-, ethyl ester	0.61	22.940	C20H38O3	326	Antifungal activity [67]
20	Hexadecanoic acid, 2-hydroxy-1-(hydroxymethyl)ethyl ester	60.66	23.530	C19H38O4	330	Antioxidant, nematicidal activity [69]
21	Octadecanoic acid, 2,3-dihydroxypropyl ester	20.28	25.081	C21H42O4	358	Antimicrobial activity [69]
22	d-Ribose, 2-deoxy-bis(thioheptyl)-dithioacetal	3.22	23.125	C19H40O3S2	380	Antifungal activity [73]

23	Glycerol 1-palmitate	-	-	C19H38O4	330	Antimicrobial activity [78]
**24**	1,3-Dipalmitin, TMS derivative	-	-	C38H76O5 Si	640	Acylglycerides [81]
**25**	2,4-Di-tert-butylphenol	0.25	12.952	C14H22O	206	It has herbicidal and allelochemical activity against (*Asystasia gangetica, Eleusine indica, Leptochloa chinensis,* and *Oldenlandia verticillate* [85]
26	Cyclo(L-prolyl-L-valine)	1.31	16.813	C10H16N2 O2	196	Class of diketopiperazines (DKPs) the compound cyclo-(L-prolyl–L-valine) activated N-acyl homoserine lactone (AHL) bioreporters, indicating that Archaea may have the ability to interact with AHL-producing bacteria within mixed communities. [92]
27	1,2-Benzenedicarboxylic acid, bis(2-methylpropyl) ester	0.64	17.015	C16H22O4	278	Antimicrobial activity[92]
28	Silane, dimethyl(docosyloxy)butoxy		-	C28H60O2 Si	456	Anti-inflammatory compound [93]
29	Silane, dimethyl(docosyloxy)butoxy		-	C28H60O2 Si	456	Anti-inflammatory compound [93]
30	Heptadecanoic acid, 16-methyl-, methyl ester	-	-	C19H38O2	298	Used in anti-skin cancer

						drug [104]
31	Heptadecane, 8-methyl	-		C18H38	254	Antimicrobial activity [106]
32	Phenol, 2,5-bis(1,1-dimethylethyl)	-	-	$C_{42}H_{63}O_4P$	662	Antioxidant [139]
33	2,6,10-Trimethyltridecane	-		C16H34	226	Antimicrobial activity [140]
34	Butylated Hydroxytoluene	-		C15H24O	220	Antimicrobial [140]
**35**	1,3,5-Trisilacyclohexane	-	-	C3H12Si3	132	Antimicrobial activity [143]
36	1H-Indene, 1-hexadecyl-2,3-dihydro-	-		C25H42	342	Anticholinestrase [145]
37	Pentacyclo[19.3.1.1(3,7).1(9,13).1(15,19)]octacosa-1(25),3,5,7(28),9,11,13(27),15,17,19(26),21,23-dodecaene-25,26,27,28-tetrol, 5,11,17,23-te		-	C44H56O4	648	Antioxidant compound [154]
